# Preparation of prolinamide with adamantane for aldol reaction catalysis in brine and separation using a poly(AN-MA-β-CD) nanofibrous film *via* host–guest interaction†

**DOI:** 10.1039/c8ra04802a

**Published:** 2018-08-07

**Authors:** Rui Wang, Enjie Xu, Zhenming Su, Haifeng Duan, Jinjin Wang, Longqi Xue, Yingjie Lin, Yaoxian Li, Zhonglin Wei, Qingbiao Yang

**Affiliations:** College of Chemistry, Jilin University 2699 Qianjin Street Changchun 130012 P. R. China yangqb@jlu.edu.cn; Security Check Jilin Province 566B Guigu Street Changchun 130012 P. R. China

## Abstract

Prolinamides with double-H potential were prepared and employed as organocatalysts in asymmetric aldol reactions. The catalyst with adamantane showed improved catalytic activity, which was further enhanced by using brine as the solvent. A series of aldol reactions in brine at 0 °C provided good yields (up to 98%) with high diastereoselectivities (>99 : 1) and enantioselectivities (>99%). The prepared catalyst was adsorbed by a nanofibrous film of poly(AN-MA-β-CD) *via* host–guest interaction in the reaction system. The catalyst was separated from the film by applying ultrasound, with a total recovery of 96.2%. The catalyst was reused up to five times without a significant change in diastereoselectivity and enantioselectivity.

## Abstract 2

Prolinamide with adamantane catalyzed the aldol reaction. The reaction of cyclohexanone with *m*-nitrobenzaldehyde assessed recyclability of catalyst. After run, the catalyst was adsorbed with nanofibrous of polymer *via* host–guest interaction.

## Graphical abstract

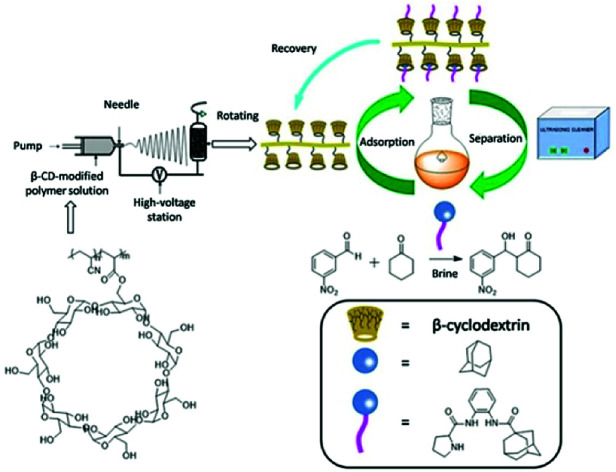

## Introduction

Asymmetric aldol reactions play an important role in carbon–carbon bond forming reactions. The design and synthesis of small molecule organocatalysts based on l-proline have been given considerable attention after List and co-workers reported the l-proline-catalyzed direct aldol reaction.^1^ In the l-proline-catalyzed reaction, the necessity of using the carboxylic acid of proline remains unclear. Since Tang and co-workers reported prolinamide-catalyzed aldol reactions of 4-nitrobenzaldehyde with acetone,^2^ effective organocatalysts have been developed based on proline to afford facile atom economic access to optically pure compounds.^3^ In 2010, Moorthy demonstrated the use of double-H potential catalysts based on l-proline and *o*-phenylenediamine to catalyze the aldol reaction of *p*-nitrobenzaldehyde with cyclohexanone in DMF in the presence of TFA with desirable yield (94%), stereoselectivity (>98%), and diastereoselectivity (97 : 3).^4^ Yang *et al.* used benzyl chloride instead of nitrobenzene sulfonic acid to synthesize prolinamide catalysts, which resulted in faster reactions with good diastereoselectivity in brine at room temperature.^3^ Nonetheless, the asymmetric reactions are usually conducted in organic solvents, such as DMF, DMSO, PhMe, and MeCN. To promote the use of environmentally friendly solvents for catalytic reaction, inorganic systems are desired as solvents.^5^ Switching organic solvents to water offers low cost, safety, and environmentally benign nature.^6^ Since Breslow reported that the acceleration of Diels–Alder reaction in water, there is a surge of interest in using water as reaction medium.^7^

**Scheme 1:**
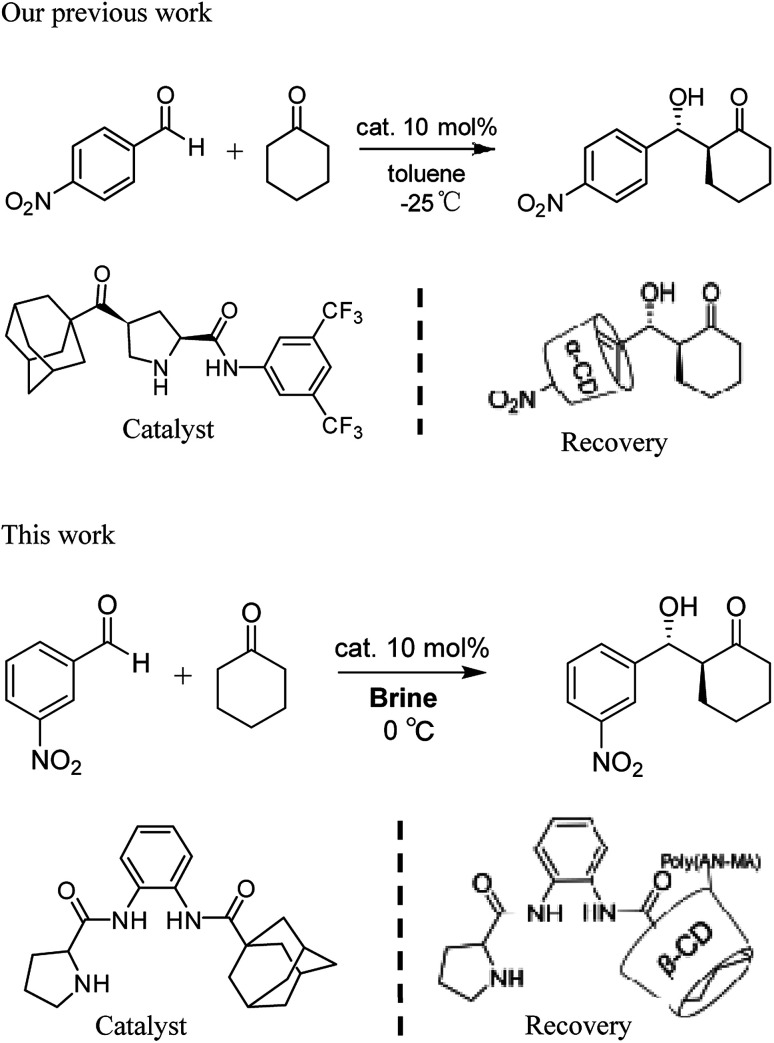
Adamantane-modified organocatalysts for asymmetric aldol reactions and recovery of the catalysts.

Recycling catalysts decreases the consumption of auxiliary substances in obtaining pure catalyst, leading to significant economic and environmental benefits.^8^ To realize recyclability of catalyst, researchers have focused on immobilizing catalysts on diverse solid supports.^9^ So far, nanoparticles and polymers have been used support, from which catalysts can be recovered and reused after centrifugation, magnetic decantation or filtration.^10^ However, a general and conventional method for immobilizing different types of organocatalysts considering stability, reactivity, and recyclability of the obtained heterogeneous materials has not been established.^11^ Moreover, supported catalysts for heterogeneous catalysis generate poor yield and stereoselectivity. Compared with supported heterogeneous catalysts, homogeneous ones provide many advantages in catalytic reaction. Nevertheless, recycling catalysts has obvious shortcomings, such as large consumption, difficult separation, and high cost. Hence, the concept wherein reaction is conducted in homogeneous medium and catalyst is recovered in heterogeneous system is highly beneficial to the development of environmentally friendly catalytic reactions. The design of self-assembled aggregates that employ host–guest interactions choosing geometrically complementary host and guest molecules is an important method of physical adsorption.^12^ Previously, we reported that assembly and separation of small organic molecules were driven by adamantane and β-cyclodextrin (β-CD) *via* host–guest interaction.^13^ The recovery of organocatalysts *via* host–guest interactions could solve a traditional problem regarding heterogeneity of solid-supported catalyst.^14^ We reported the aldol product of *p*-nitrobenzaldehyde with cyclohexanone in toluene at –20 °C was separated by α-CD from the reaction system in Scheme 1.^15^ However, α-CD being easy to dissolve in water is to the disadvantage of the overall recycling of the product. Therefore, the developing the water insoluble α-CD or β-CD is of the essence.

**Fig. 1:**
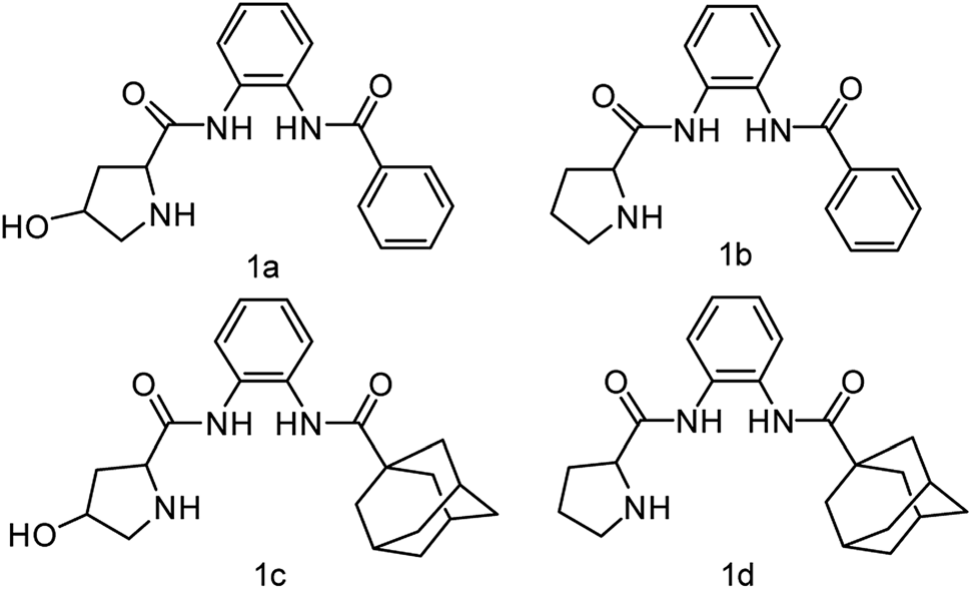
Adamantane-modified prolinamide catalysts.

**Scheme 2:**
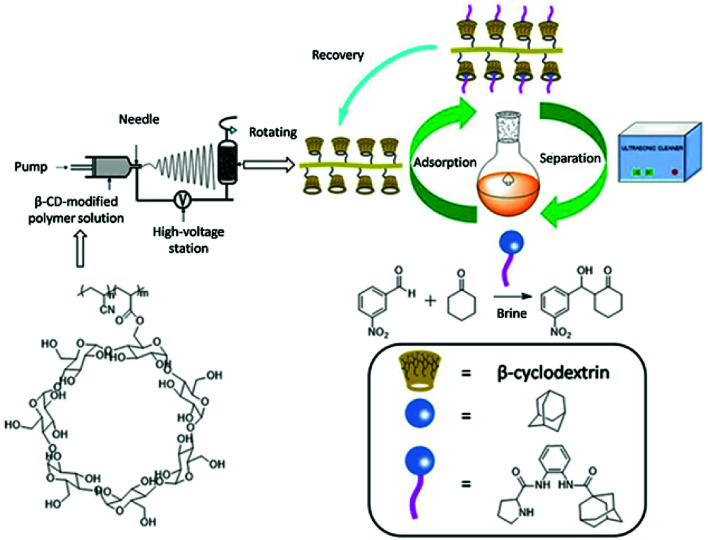
Illustration for recyclability of catalyst.

Herein, we synthesize adamantine-modified catalysts separated by supported β-CD *via* host–guest interaction. *O*-phenylenediamine catalysts with double hydrogen potential based on l-proline or *trans*-4-hydroxy-l-proline were synthesized to catalyze aldol reactions of ketone and aldehyde (Fig. 1). Prolinamides with 1-adamantane carboxylic acid could improve the catalytic activity by replacing benzoic acid. Improved results were obtained by switching the solvent to an inorganic system. The brine was used as medium at 0 °C for catalyzing the aldol reaction of aldehyde and ketone, resulting in excellent yields with good stereoselectivity and diastereoselectivity. β-CD-modified polymer (poly(AN-MA-β-CD)) was synthesized by using co-polymer (poly(AN-MA)) and β-CD, and then nanofibrous film was prepared by electrospinning. A flowchart of catalyst separation from the film is described in Scheme 2. After each run, the catalyst could be adsorbed by the film *via* host–guest interaction in the reaction system and separated by washing with MeOH under ultrasound from the film.

## Results and discussion

### Asymmetric aldol reaction

Initially, we employed chiral prolinamide catalysts 1a–1d to screen the direct aldol reaction of *p*-nitrobenzaldehyde with cyclohexanone at room temperature (Scheme 2). A series of experiments were then performed under different reaction conditions (different catalysts, loading, and solvents). The results are summarized in Table 1. In the beginning of the experiment (Table 1, entries 1–4), the reactions were catalyzed by different prolinamides (10 mol%) at room temperature for 36 h in DCM. Interestingly, all catalysts provided the desired aldol products with good yields. The aldol reactions in the presence of prolinamides 1a and 1b generated 69% and 77% of enantioselectivity (Table 1, entries 1 and 2). When used to catalyze the aldol reaction of 4-nitrobenzaldehyde and cyclohexanone, compounds 1c and 1d afforded good yields with higher diastereoselectivity and enantioselectivity than 1a and 1b. This result may be due to replacement of benzoic acid with the large steric hindrance of adamantane. Compared with compound 1c, the desired product was formed in 91% yield with 89% stereoselectivity when catalyst 1d was employed (Table 1, entry 4). Compared to results reported respectively by Moorthy' and Yang' research groups, the catalyst 1d improves the catalytic activity. Our group have reported that the adamantine provided by 4-substitution position based on l-proline played an important role in diastereoselectivity and enantioselectivity.^15^ In addition, catalysts with adamantyl group were employed in other asymmetrical reactions provided good diastereoselectivity or enantioselectivity.^14,16^ Although catalyst loading was varied from 10 mol% to 30 mol%, no further improvement in diastereoselectivity and enantioselectivity was observed (Table 1 entries 4–6). Poor diastereoselectivity was obtained at low catalyst loading of 5 mol% or 2 mol% (Table 1, entries 7–8). Considering that the catalyst 1d of 10 mol% afforded a good conversion with desirable diastereoselectivity and enantioselectivity, entry 4 was selected as an optimal reaction. Compound 1d of 10 mol% was employed as catalyst in nine solvents with different polarities at room temperature for 36 h of reaction under vigorous stirring. The aldol reactions being conducted in nine solvents investigated showed no significant change in yield (from 90% to 96%) and diastereoselectivity (from 96 : 4 to 99 : 1). However, the stereoselectivity was significantly influenced on ranging from 82% (MeOH) to 95% (brine). Among the seven other organic solvents, high stereoselectivity (91%) was obtained when THF was used as solvent (Table 1, entry 9). Compared with organic solvents, the inorganic medium was found to be superior in terms of stereoselectivity. For example, brine provided the best stereoselectivity of the aldol product (95%) to proved itself be the best (Table 1, entry 16). Therefore, brine was employed as solvent in further study.

**Table 1:** Screening catalysts and solvents^a^

Entry	Catalyst	Mol%	Solvent	Yield^b^ (%)	dr^c^ (*anti*/*syn*)	ee^c^ (%)
1	1a	10	DCM	89	91 : 9	69
2	1b	10	DCM	96	88 : 12	77
3	1c	10	DCM	93	97 : 3	82
4	1d	10	DCM	91	97 : 3	89
5	1d	20	DCM	93	97 : 3	89
6	1d	30	DCM	96	97 : 3	89
7	1d	5	DCM	90	95 : 5	88
8	1d	2	DCM	85	95 : 5	88
9	1d	10	THF	95	97 : 3	91
10	1d	10	HEX^d^	94	99 : 1	86
11	1d	10	EtOAc	96	99 : 1	87
12	1d	10	MeCN	90	96 : 4	86
13	1d	10	MeOH	92	99 : 1	82
14	1d	10	DMSO	92	98 : 2	86
15	1d	10	H_2_O	91	97 : 3	90
16	1d	10	Brine^e^	95	98 : 2	95

a Reaction conditions: *p*-nitrobenzaldehyde (0.5 mmol), cyclohexanone (10 equiv.), solvent (1.0 mL), room temperature, 36 hours with vigorous stirring.

b Combined yields of isolated.

c Determined by HPLC with a chiral AD-H column.

d *n*-hexane.

e Solubility of 20%.

To optimize the reaction conditions, we determined the effect of acid additive^17^ and reaction temperature.^18^ The most relevant results were detailed in Table 2. As indicated by entries 1–5 in Table 2, 10 mol% organic acid (such as 4-nitrobenzoic acid, benzoic acid, trifluoroacetic acid, acetic acid and methanolic acid) was added to the reaction in brine at room temperature for 36 h. When the reactions were carried out in the presence of an organic acid, the yields were slightly improved, while diastereoselectivity and stereoselectivity showed no significant differences. A high efficiency was obtained when no additive was added to the reaction mixture (Table 2, entry 6). In an effort to improve the catalytic activity, the reaction temperature further was investigated. By lowering the reaction temperature to 10 °C, the reaction went smoothly to form the desired aldol product without significant change in diastereoselectivity and stereoselectivity. A further reduced of the reaction temperature to 0 °C, a slight increase of stereoselectivity was observed. Lengthening reaction time to 48 h drove the reaction towards completion (yield 98%). Entry 9 was performed under the optimized reaction conditions.

**Table 2:** Screening additive and temperature^a^

Entry	Additive	*T* (°C)	Yield^b^ (%)	dr^c^ (*anti*/*syn*)	ee^c^ (%)
1	4-NO_2_PhCOOH	rt	98	97 : 3	94
2	PhCOOH	rt	98	96 : 4	95
3	CF_3_OOH	rt	96	96 : 4	93
4	CH_3_OOH	rt	95	95 : 5	94
5	HCOOH	rt	96	95 : 5	93
6	—	rt	95	98 : 2	95
7	—	10	95	98 : 2	95
8	—	0	93	98 : 2	96
9^d^	—	0	98	98 : 2	96

a Reaction conditions: *p*-nitrobenzaldehyde (0.5 mmol), cyclohexanone (10 equiv.), catalyst 1d (0.05 mmol, 10 mol%), solvent (1.0 mL), room temperature, 36 hours with vigorous stirring.

b Combined yields of isolated.

c Determined by HPLC with a chiral AD-H column.

d Vigorous stirring for 48 hours.

After determining the optimal reaction conditions, we further explored the substrate scope of aldol reactions; the results are summarized in Table 3. In the initial experiment, aldol reactions of cyclohexanone with different aldehydes were investigated at 0 °C. Satisfactorily, reactions gave the desired products in good yields with high diastereoselectivity and enantioselectivity. Benzaldehydes substituents with different electronic characters were studied, including electronic-withdrawing (NO_2_, CN, CF_3_, Cl, Br and F), electronic-neutral (H) and electronic-donating (OCH_3_ and CH_3_) groups. Aromatic aldehydes with *ortho*-, *meta*-, and *para*-substituents generated aldol products with similar yields. Aromatic aldehydes with strong electronic-withdrawing group underwent aldol reactions to provide the corresponding products in excellent yields with high diastereoselectivity and enantioselectivity (Table 3, entries 1–7). Moderate conversions were obtained by benzaldehydes with weak electronic-withdrawing and electronic-neutral groups (Table 3, entries 8–16). Compared to benzaldehyde, 2-naphthaldehyde provides poor diastereoselectivity and enantioselectivity. Aldehydes with electronic-donating were found to have very low reactivity and generated aldol products in <10% yield (Table 3, entries 17–19). Ketone with different substituents were then investigated. In contrast to the aldehyde substrates, poor diastereoselectivity was produced by cyclopentanone (Table 3, entry 20). When 2-butanone as reagent led to the formation of regioisomers, three pairs of enantiomers were obtained with poor enantioselectivities (Table 3, entry 21). Influenced by water hydrolyzing and steric hinderance, 2-butanone was easily transformed into stable carbanion. Therefore, the aldol product with one pair of enantiomer was dominant of 3-pentone. The aldol reaction in the presence of acetone also provided moderate yield with poor enantioselectivity (Table 3, entry 22). Enantioselectivities of large-scale asymmetric aldol reactions were reduced because a lot of aldehyde did not dissolve in brine under unevenly stringing.

**Table 3:** Scope for aldol reaction of ketones with aldehydes employing catalyst 1d^a^

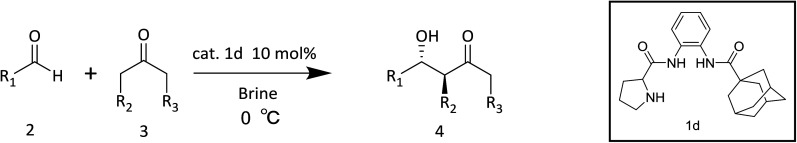						
Entry	Aldehyde (2) R_1_	Ketone (3) R_2_, R_3_	Product (4)	Yield^b^ (%)	dr^c^ (*anti*/*syn*)	ee^c^ (%)
1	*p*-NO_2_Ph	–(CH_2_)_3_–	4a	98	98 : 2	96
2	*m*-NO_2_Ph	–(CH_2_)_3_–	4b	97	99 : 1	98
3	*o*-NO_2_Ph	–(CH_2_)_3_–	4c	97	>99 : 1	97
4	*p*-CNPh	–(CH_2_)_3_–	4d	93	99 : 1	97
5	*m*-CNPh	–(CH_2_)_3_–	4e	90	97 : 3	97
6	*o*-CNPh	–(CH_2_)_3_–	4f	91	94 : 6	97
7	*p*-CF_3_Ph	–(CH_2_)_3_–	4g	94	98 : 2	97
8	2,4-Cl_2_Ph	–(CH_2_)_3_–	4h	90	99 : 1	98
9	*p*-ClPh	–(CH_2_)_3_–	4i	57	99 : 1	98
10	*m*-ClPh	–(CH_2_)_3_–	4j	54	99 : 1	98
11	*o*-ClPh	–(CH_2_)_3_–	4k	59	99 : 1	99
12	*p*-BrPh	–(CH_2_)_3_–	4l	50	>99 : 1	98
13	*o*-BrPh	–(CH_2_)_3_–	4m	55	99 : 1	97
14	*p*-FPh	–(CH_2_)_3_–	4n	46	98 : 2	95
15	Ph	–(CH_2_)_3_–	4o	49	99 : 1	97
16	2-Naphthyl	–(CH_2_)_3_–	4p	36	88 : 12	89
17	Furan	–(CH_2_)_3_–	4q	<10		
18	*p*-OCH_3_Ph	–(CH_2_)_3_–	4r	<10		
19	*p*-CH_3_Ph	–(CH_2_)_3_–	4s	<10		
20	*p*-NO_2_Ph	–(CH_2_)_2_–	4t	90	67 : 33	64
21	*p*-NO_2_Ph	H, CH_3_	4u	88	—	75
22	*p*-NO_2_Ph	H, H	4v	95	—	22

a Reaction conditions: aldehyde (0.5 mmol), ketone (10 equiv.), catalyst 1d (0.05 mmol, 10 mol%), solvent (1.0 mL), vigorous stirring with correspond time.

b Combined yields of isolated.

c Determined by HPLC with chiral AD-H column, chiral OD-H column and chiral AS-H column.

### FTIR analysis

**Fig. 2:**
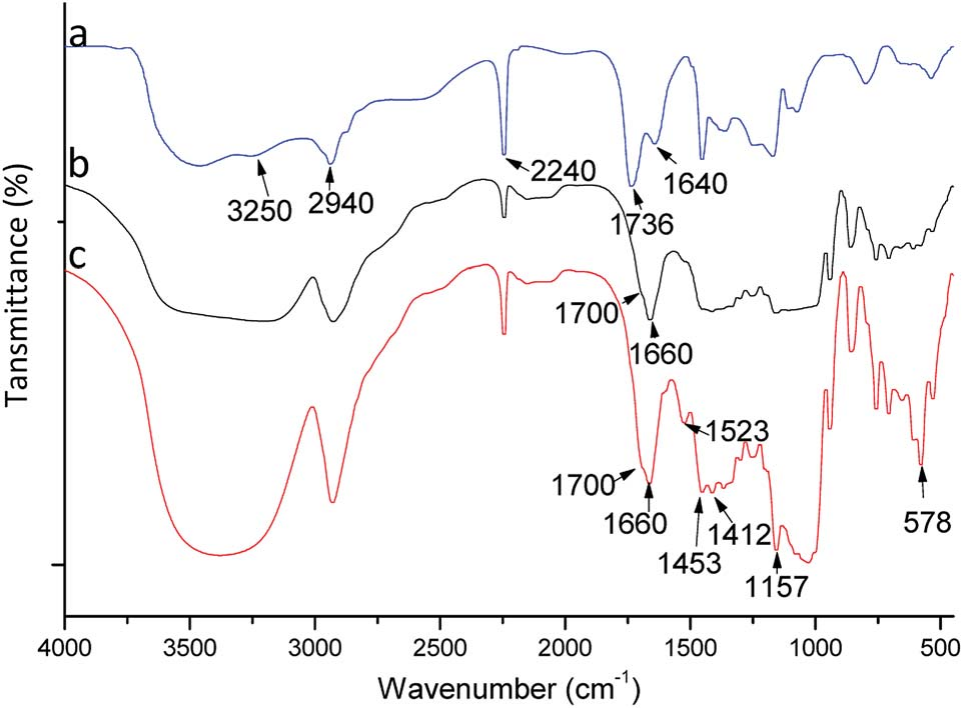
FTIR spectra of poly(AN-MA) (a), poly(AN-MA-β-CD) (b) and catalyst into poly(AN-MA-β-CD) (c).

To confirm the composition and grafting, polymers were studied by FTIR spectroscopy (Fig. 2). A FTIR spectrum for poly(AN-MA) is shown in Fig. 2(a). Fig. 2(a) exhibits peaks at 2940 and 2240 cm^−1^, which were attributed to asymmetric C–H stretching from (–CH_2_–)_*n*_ and asymmetric C–N stretching from cyano group. The characteristic peak of cyano group could be observed in Fig. 2(b) and (c). The broad peak at 3340 cm^−1^ was the symmetric –OH stretching from β-CD molecule in Fig. 2(b), which could prove that β-CD was grafted on the polymer surface. In Fig. 2(c), the characteristic peaks of benzene framework vibration peaks can be observed at 1523, 1453, and 1412 cm^−1^. These bands, however, are not present in Fig. 2(a) and (b). Thus, the catalyst could successfully bind into poly(AN-MA-β-CD).

### SEM analysis

**Fig. 3:**
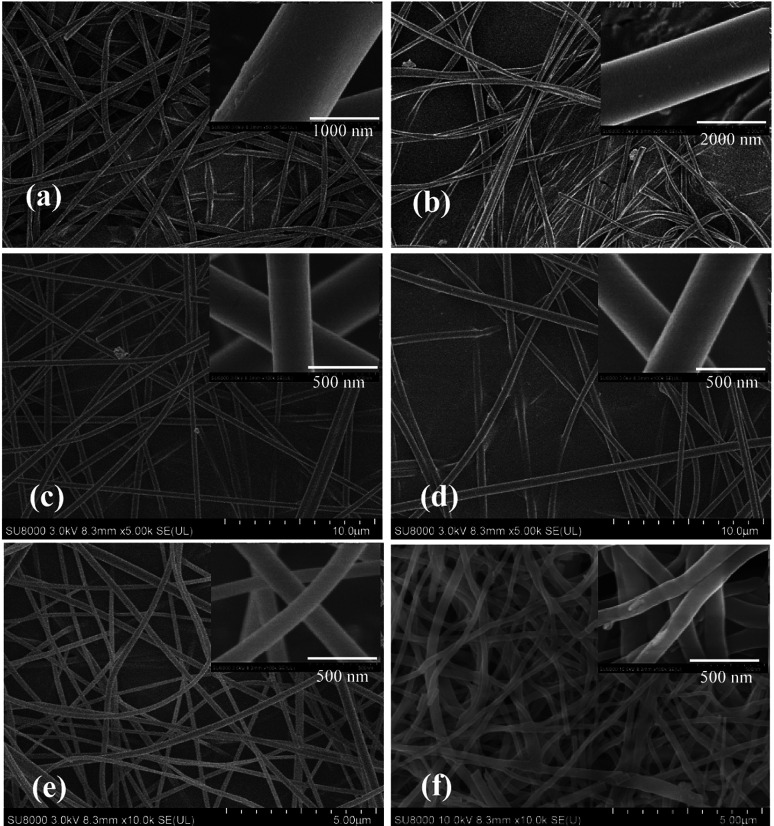
SEM images of different mass fraction with poly(AN-MA-β-CD) in DMF: (a) 36%, (b) 33%, (c) 30%, (d) 25%, (e) 20%, and (f) catalyst bind to poly(AN-MA-β-CD).

Typical SEM images of nanofibrous films of poly(AN-MA-β-CD) are shown in Fig. 3. It could be clearly seen that the membranes were composed of numerous and randomly oriented nanofibers. When the mass fraction of poly(AN-MA-β-CD) in DMF was <20%, a fibrous membrane was not formed. At mass fraction of >36%, poly(AN-MA-β-CD) did not dissolve in DMF in a short time. The SEM images showed that this membrane feature was affected by the mass fraction from 20% to 36% (Fig. 3). Fig. 3(a) and (b) showed that the nanofibrous film have an average diameter of exceed 1 μm. With further reduction in the mass fraction, the diameter of the resulting fibrous membranes (Fig. 3 (c)–(e)) had decreased. In particular, Fig. 3(c) and (d) respectively show an average diameter of about 440 nm and 430 nm with smooth surfaces. At mass fraction of 20%, the average diameter of the film was approximately 160 nm with smooth surface (Fig. 3(e)). In general, 20% poly(AN-MA-β-CD) formed plentiful fibrous membranes. Compared with Fig. 3(e), the result in Fig. 3(f) showed coarser surfaces and larger diameter.

### Recyclability of catalyst

In the next moment, we further evaluated the recyclability of the catalyst in the direct aldol reactions. According to related reports, benzene and adamantane can be recovered by CD *via* host–guest interaction.^12^ The reaction of cyclohexanone with *m*-nitrobenzaldehyde was selected as a model for assessing the recyclability of catalyst because *m*-nitrobenzaldehyde with steric hindrance did not easily turn into the β-CD cavity and adamantine-modified catalyst was recovered by fibrous membrane *via* host–guest interaction from the reaction medium. The recycling process of catalyst is shown in Scheme 2. The adsorption rate of catalyst in mixture of MeOH/H_2_O 98.5%, and the separation rate from the fibrous membrane was 97.7%. Therefore, the total recovery rate was 96.2%. The relevant results about recyclability of catalyst are summarized in Table 4. In the first three recycles, conversions were above 95% with excellent diastereoselectivities and enantioselectivities under the same reaction time. Prolonging the reaction time could also provide good yields with desirable diastereoselectivities from the third cycle. In the sixth run, the yield was 92% with ee value of 92%. As shown in Table 4, the catalyst can also be reused for up to five times without showing significant change in diastereoselectivity and enantioselectivity.

**Table 4:** Recycling experiments^a^

Entry	Time (h)	Yield^b^ (%)	dr^c^ (*syn*/*anti*)	*ee* c (%)
1	48	97	99 : 1	98
2	48	98	>99 : 1	99
3	48	95	98 : 2	97
4	60	95	99 : 1	97
5	60	90	98 : 2	96
6	72	92	94 : 6	92

a Reaction conditions: *m*-nitrobenzaldehyde (0.5 mmol), cyclohexanone (10 equiv.), catalyst 1d (0.05 mmol, 10 mol%) only being added at first run, solvent (1.0 mL), 0 °C.

b Combined yields of isolated.

c Determined by HPLC with a chiral AD-H column.

## Conclusions

In summary, organocatalysts based on l-proline or *trans*-4-hydroxy-l-proline with double-H potential were synthesized. Catalyst 1d (10 mol%) demonstrated very efficient performance for catalyzing direct aldol reactions. The use of brine as solvent afforded aldol products in excellent yields (up to 98% yield) with high diastereoselectivity (>99 : 1) and enantioselectivity (>99%). A nanofibrous film of 20% poly(AN-MA-β-CD) was formed by electrospinning and characterized by FTIR and SEM analyses. After each run, the catalyst adsorbed on the film *via* host–guest interaction in the reaction system and then separated from the film using ultrasound. The total recovery rate of the catalyst was 96.2%, and the catalyst could be reused for up to five times without exhibiting significant change in diastereoselectivity and enantioselectivity.

## Experimental

### General procedure

All chemicals were used as received unless otherwise noted. All reagents were purchased from J&K or Sigma-Aldrich Chemical Co. and were used without any further purification. Solvents were dried according to standard procedures. Reactions were monitored by thin-layer chromatography (TLC) carried out on silica gel 60F254 plates. Flash column chromatography was performed using silica gel (200–300 mesh, Qingdao Haiyang Chemical Co.). ^1^H and ^13^C NMR spectra were taken on a Bruker AV-400 MHz spectrometer. All chemical shifts (*δ*) were given in ppm. Chemical shifts (*δ* ppm) are relative to the resonance of the deuterated solvent as the internal standard (CDCl_3_, *δ* 7.26 ppm for proton NMR, *δ* 77.36 ppm for carbon NMR; DMSO-d6, *δ* 2.54 ppm for proton NMR, *δ* 40.45 ppm for carbon NMR). Data are presented as follows: chemical shift, integration, multiplicity (br = broad, s = singlet, d = doublet, t = triplet, q = quartet, m = multiplet) and coupling constant in hertz (Hz). Mass spectra were recorded on the Bruker Agilent 1290 MicrOTOF Q II. Melting points were measured on a melting point apparatus and were uncorrected. The ee values determination was carried out using chiral HPLC (Waters) with Chiracel AD-H column, Chiracel OD-H column and Chiracel AS-H column. Optical rotations were measured on a Shanghai Shen Guang SGW-2 Polarimeter at *λ* = 589 nm. Optical rotations are reported as follows: [*α*]^25^_D_ (*c* = g/100 mL, solvent). IR spectra were recorded on a Bruker Vector-22 spectrometer. UV-Vis spectra were recorded on a Hitachi U-3010 UV-Vis spectrophotometer. High voltage power (0–50 kV) DW-P503-1AC (Tianjin, China), and scanning electron microscope (SEM) were also used.

### Synthesis of catalysts: the catalysts were prepared according to previous works^3,4^

To a flask containing *o*-phenylenediamine (1.08 g, 10.0 mmol, 1.0 equiv.) dissolved in dry CH_2_Cl_2_ (30 mL) at 0 °C, and a solution of acid (10.0 mmol, 1.0 equiv.), DMAP (0.98 g, 8.0 mmol, 0.8 equiv.), and EDCI (2.30 g, 12.0 mmol, 1.2 equiv.) in CH_2_Cl_2_ (20 mL) was added into solvent after 5 min with vigorous stirring. The resulting reaction mixture was stirred at room temperature for overnight. The solvent was evaporated. The aqueous layer was extracted with ethyl acetate. The combined organic layer was dried (MgSO_4_) and then evaporated under reduced pressure to the crude products which was further purified *via* silica gel column chromatography (EtOAc : PE = 1 : 5) to afford compound.

Boc-l-hydroxyproline or Boc-l-proline (6.0 mmol, 1.2 equiv.) was dissolved in dry CH_2_Cl_2_ (20 mL) and cooled to 0 °C. And DMAP (0.49 g, 4.0 mmol, 0.8 equiv.) and EDCI (1.15 g, 6.0 mmol, 1.2 equiv.) were added. A solution of phenylamine 1 (1.06 g, 5.0 mmol) in dry CH_2_Cl_2_ (20 mL) was then added dropwise after 15 min with vigorous stirring. The resulting reaction mixture was stirred at room temperature and monitored by TLC. After completion of the reaction, the mixture was partitioned between EtOAc and water. The organic layer was washed with saturated brine. The combined organic layer was dried (MgSO_4_) and then evaporated under reduced pressure to crude product.

The crude product was then dissolved in CH_2_Cl_2_ (20 mL) and cooled to 0 °C. Trifluoroacetic acid (10.0 equiv.) was added dropwise to mixture solution and further stirred at room temperature for another 4 h. After the reaction was completed, mixture was quenched with saturated NaHCO_3_ aqua. The aqueous layer was extracted with ethyl acetate, and the organic layer was successively washed with water and another portion of saturated brine, dried (MgSO_4_) and evaporated under reduced pressure. The oily residue was purified by silica gel chromatography (ethyl acetate/methanol (3% Et_3_N) = 10 : 1) to give compound.

#### (*S*)-*N*-(2-Benzamidophenyl)-4-hydroxyprolinamide (1a)

White solid (1.12 g, yield 69%). Mp = 164–165 °C, [*α*]^25^_D_ = +11.6 (*c* = 0.5, CH_3_OH). ^1^H NMR (400 MHz, DMSO) *δ* = 10.22 (d, *J* = 15.4 Hz, 1H), 8.05 (dd, *J* = 12.8, 7.9 Hz, 2H), 7.65 (t, *J* = 7.2 Hz, 1H), 7.59 (t, *J* = 7.4 Hz, 1H), 7.40 (d, *J* = 7.8 Hz, 1H), 7.32 (t, *J* = 7.7 Hz, 1H), 7.19 (t, *J* = 7.6 Hz, 1H), 4.70 (d, *J* = 2.6 Hz, 1H), 4.15 (s, 1H), 3.91 (t, *J* = 8.2 Hz, 1H), 3.40 (s, 1H), 2.64 (dt, *J* = 11.5, 7.4 Hz, 1H), 2.10–2.00 (m, 1H), 1.83–1.73 (m, 1H) ppm. ^13^C NMR (101 MHz, DMSO) *δ* = 174.34, 166.77, 134.99, 134.46, 132.72, 129.41, 129.20, 128.59, 128.15, 127.59, 124.83, 122.56, 72.33, 60.90, 55.65, 40.54 ppm. HRMS (ESI): calcd for C_18_H_20_N_3_O_3_^+^ [M + H]^+^ 326.1499; found 326.1500.

#### (*S*)-*N*-(2-Benzamidophenyl)prolinamide (1b)

White solid (0.93 g, yield 60%). Mp = 159–160 °C, [*α*]^25^_D_ = −6.0 (*c* = 0.5, CH_3_OH). ^1^H NMR (400 MHz, DMSO): *δ* = 10.24 (d, *J* = 13.2 Hz, 1H), 8.07 (dd, *J* = 16.6, 7.8 Hz, 2H), 7.62 (dt, *J* = 14.7, 7.1 Hz, 2H), 7.43–7.28 (m, 1H), 7.19 (t, *J* = 7.5 Hz, 1H), 3.73 (dd, *J* = 9.1, 4.6 Hz, 1H), 3.20 (s, 1H), 2.78 (dd, *J* = 11.7, 4.7 Hz, 1H), 2.65–2.56 (m, 1H), 2.09–1.96 (m, 1H), 1.80 (dd, *J* = 12.0, 5.6 Hz, 1H), 1.57 (dt, *J* = 13.3, 6.6 Hz, 1H) ppm. ^13^C NMR (101 MHz, DMSO): *δ* = 174.50, 166.73 (s), 134.93, 134.62, 132.73, 129.42, 129.14, 128.56, 128.18, 127.63, 61.58, 47.45, 31.41, 26.75 ppm. HRMS (ESI): calcd for C_18_H_20_N_3_O_2_^+^ [M + H]^+^ 310.1550; found 310.1550.

#### (*S*)-*N*-(2-(Adamantane-1-carboxamidophenyl))-4-hydroxyprolinam-ide (1c)

White solid (1.36 g, yield 71%). Mp = 209–210 °C, [*α*]^25^_D_ = −11.2 (*c* = 0.5, CH_3_OH). ^1^H NMR (400 MHz, DMSO): *δ* = 10.02 (s, 1H), 9.11 (s, 1H), 8.01 (d, *J* = 8.2 Hz, 1H), 7.24 (dd, *J* = 12.1, 4.5 Hz, 3H), 7.15–7.09 (m, 1H), 4.81 (s, 1H), 4.25 (s, 1H), 3.95 (t, *J* = 8.3 Hz, 1H), 3.04 (q, *J* = 7.2 Hz, 2H), 2.91–2.81 (m, 3H), 2.08 (d, *J* = 9.7 Hz, 4H), 2.02–1.92 (m, 8H), 1.80–1.72 (m, 8H), 1.18 (t, *J* = 7.3 Hz, 3H). ^13^C NMR (101 MHz, DMSO): *δ* = 177.40, 174.08, 134.18, 129.54, 128.15, 127.07, 124.67, 122.26, 72.25, 60.88, 55.87, 46.61, 41.46, 39.57, 37.06, 28.64 ppm. HRMS (ESI): calcd for C_22_H_30_N_3_O_3_^+^ [M + H]^+^ 384.2282; found 384.2282.

#### (*S*)-*N*-(2-(Adamantane-1-carboxamidophenyl))prolinamide (1d)

White solid (1.23 g, yield 67%). Mp = 213–214 °C, [*α*]^25^_D_ = −24.4 (*c* = 0.5, CH_3_OH). ^1^H NMR (400 MHz, DMSO): *δ* = 10.08 (s, 1H), 9.01 (s, 1H), 7.82 (d, *J* = 7.9 Hz, 1H), 7.36–7.32 (m, 1H), 7.27–7.22 (m, 1H), 7.18 (dd, *J* = 10.8, 4.4 Hz, 1H), 4.05 (dd, *J* = 8.7, 6.0 Hz, 1H), 3.17–2.97 (m, 3H), 2.22 (dt, *J* = 15.8, 8.0 Hz, 1H), 2.06 (s, 3H), 1.96 (d, *J* = 7.1 Hz, 6H), 1.84–1.68 (m, 10H), 1.21 (t, *J* = 7.3 Hz, 1H) ppm. ^13^C NMR (101 MHz, DMSO): *δ* = 177.19, 171.96, 132.98, 130.61, 127.78, 126.74, 125.49, 123.75, 61.15, 47.35, 41.52, 39.53, 37.02, 31.04, 28.61, 25.95 ppm. HRMS (ESI): calcd for C_22_H_30_N_3_O_2_^+^ [M + H]^+^ 368.2333; found 368.2333.

### The preparation of poly(AN-MA-β-CD)

The poly(AN-MA-β-CD) was prepared according to previous works in our group.^19^ AN (30 g, 0.57 mol) and MA (7.21 g, 0.1 mol) were dissolved in dry DMF (149 g) at 0 °C for vigorous string 15 min. And then PVP (0.037 g) and AIBN (0.372 g) was added into solvent. The resulting reaction mixture was stirred at 70 °C for overnight. After reaction, a large of distilled water was added and precipitate was observed. The solid was washed with distilled water another three times and dried at vacuum oven for overnight to give the poly(AN-MA) 18.23 g (yield: 49%).

The polymer (5 g) dissolved in dry DMF (80 mL) at 0 °C, and then DMAP (19.17 g, 0.1 mol) and EDCI (1.22 g, 0.01 mol) was added into solvent after vigorous stirring with 5 min. β-CD in dry DMF (500 mL) was added dropwise under 0 °C. The resulting reaction mixture was stirred at room temperature for overnight. The solvent was evaporated after complete reaction. A large of distilled water was added and solid was precipitated. The solid was washed with distilled water another three times and dried at vacuum oven for overnight to give poly(AN-MA-β-CD) 11.03 g.

### The preparation of fibrous film

The poly(AN-MA-β-CD) dissolved in DMF was changed into fibrous membrane by electrospinning, and the preparation flowchart of fibrous membrane was shown in Scheme 2.^20^ The electrospinning solution was moved into the 5.0 mL injector with a stainless-steel needle of inner diameter about 1 mm, and the distance from the needle to the receiving plate was adjusted to 15–18 cm. Fibrous membrane was collected on aluminium foil with a liquid flow rate of 1.0 mL h^−1^ and a voltage of 15.4 kV. After 4 h of continuous electrospinning, aluminium foil was placed into the oven at 40 °C for 6 h.

### General procedure for asymmetric aldol reaction

The following procedure for the reaction of cyclohexanone with *p*-nitrobenzaldehyde in brine using catalyst 1d was representative. To a mixture of catalyst 1d (18.4 mg, 0.05 mmol) and *p*-nitrobenzaldehyde (75 mg, 0.5 mmol) in brine (1 mL) was added under air in a closed system. The reaction mixture was stirred at 0 °C for 15 min, and then cyclohexanone (10 equiv.) was added. After the reaction mixtures were stirred for 48 h, the mixtures were quenched with 2 M ammonium chloride solution and extracted with ethyl acetate. The organic layer was dried over Na_2_SO_4_, filtered, and concentrated to give pure aldol product after flash column chromatography (silica gel, petroleum ether/ethyl acetate = 3/1). The product was a light yellow or white solid. The absolute configuration of aldol products was extrapolated by comparison of the HPLC-data with known literatures.^15,21^

#### (2*S*,1′*R*)-2-(Hydroxy-(4-nitrophenyl)methyl)cyclohexan-1-one (4a)

Reaction time: 48 h; yield: 98%; light yellow solid. Enantiomeric excess: 96%, determined by HPLC with a Chiral-pack AD-H column (90 : 10 hexane : 2-propanol), 1 mL min^−1^; 254 nm, 25 °C; *t*_R_ (major) = 31.2 min, *t*_R_ (minor) = 23.7 min. ^1^H NMR (400 MHz, DMSO): *δ* = 8.20 (t, *J* = 7.6 Hz, 2H), 7.63 (t, *J* = 8.9 Hz, 2H), 5.54 (d, *J* = 27.0 Hz, 1H), 5.12 (d, *J* = 7.0 Hz, 1H), 2.71 (ddd, *J* = 20.8, 10.9, 5.5 Hz, 1H), 2.37 (s, 2H), 1.58 (s, 5H), 1.20 (td, *J* = 19.3, 9.1 Hz, 1H) ppm. ^13^C NMR (101 MHz, DMSO): *δ* = 211.39, 153.81, 147.23, 128.81, 128.10, 123.72, 69.31, 57.15, 42.25, 30.20, 28.12, 24.31 ppm.

#### (2*S*,1′*R*)-2-(Hydroxy-(3-nitrophenyl)methyl)cyclohexan-1-one (4b)

Reaction time: 48 h; yield: 97%; light yellow solid. Enantiomeric excess: 98%, determined by HPLC with a Chiralpack AD-H column (95 : 5 hexane : 2-propanol), 1 mL min^−1^; 254 nm, 25 °C; *t*_R_ (major) = 34.8 min, *t*_R_ (minor) = 44.5 min. ^1^H NMR (400 MHz, DMSO): *δ* = 8.17 (s, 1H), 8.08 (d, *J* = 8.3 Hz, 1H), 7.76 (d, *J* = 7.1 Hz, 1H), 7.58 (t, *J* = 7.9 Hz, 1H), 5.52 (d, *J* = 4.4 Hz, 1H), 5.13–5.03 (m, 1H), 2.70 (d, *J* = 4.7 Hz, 1H), 2.30 (s, 2H), 1.78 (d, *J* = 25.2 Hz, 2H), 1.55 (s, 3H), 1.15 (d, *J* = 11.8 Hz, 1H) ppm. ^13^C NMR (101 MHz, DMSO): *δ* = 211.75, 148.51, 146.96, 134.61, 130.25, 122.83, 122.35, 71.20, 58.14, 42.21, 30.31, 28.30, 24.15 ppm.

#### (2*S*,1′*R*)-2-(Hydroxy-(2-nitrophenyl)methyl)cyclohexan-1-one (4c)

Reaction time: 48 h; yield: 97%; light yellow solid. Enantiomeric excess: 97%, determined by HPLC with a Chiralpack AD-H column (95 : 5 hexane : 2-propanol), 1 mL min^−1^; 254 nm, 25 °C; *t*_R_ (major) = 16.8 min, *t*_R_ (minor) = 19.1 min. ^1^H NMR (400 MHz, DMSO): *δ* = 7.81 (d, *J* = 8.1 Hz, 1H), 7.69 (d, *J* = 4.2 Hz, 2H), 7.50 (dt, *J* = 8.3, 4.3 Hz, 1H), 5.76 (s, 1H), 5.60 (d, *J* = 4.5 Hz, 1H), 2.89–2.69 (m, 1H), 2.46–2.35 (m, 1H), 2.28 (dd, *J* = 12.8, 4.4 Hz, 1H), 1.90 (s, 1H), 1.75–1.66 (m, 1H), 1.63–1.49 (m, 2H), 1.44 (d, *J* = 13.2 Hz, 1H), 1.37–1.21 (m, 1H) ppm. ^13^C NMR (101 MHz, DMSO): *δ* = 211.13, 149.60, 138.36, 133.34, 130.33, 129.04, 124.09, 67.28, 57.85, 42.59, 31.28, 28.57, 24.68 ppm.

#### (2*S*,1′*R*)-2-(Hydroxy-(4-cyanophenyl)methyl)cyclohexan-1-one (4d)

Reaction time: 48 h; yield: 93%; white solid. Enantiomeric excess: 97%, determined by HPLC with a Chiralpack OD-H column (95 : 5 hexane : 2-propanol), 1 mL min^−1^; 220 nm, 25 °C; *t*_R_ (major) = 25.7 min, *t*_R_ (minor) = 37.3 min. ^1^H NMR (400 MHz, DMSO): *δ* = 7.78 (d, *J* = 8.0 Hz, 2H), 7.53 (d, *J* = 8.0 Hz, 2H), 5.46 (s, 1H), 5.03 (d, *J* = 7.2 Hz, 1H), 2.67 (dd, *J* = 13.2, 8.9 Hz, 1H), 2.34 (t, *J* = 6.2 Hz, 2H), 1.92–1.69 (m, 2H), 1.68–1.45 (m, 3H), 1.15 (dd, *J* = 20.6, 10.6 Hz, 1H) ppm. ^13^C NMR (101 MHz, DMSO): *δ* = 211.70, 150.31, 132.75, 128.79, 119.86, 110.61, 71.62, 58.18, 42.13, 30.44, 28.37, 24.05 ppm.

#### (2*S*,1′*R*)-2-(Hydroxy-(3-cyanophenyl)methyl)cyclohexan-1-one (4e)

Reaction time: 48 h; yield: 90%; white solid. Enantiomeric excess: 97%, determined by HPLC with a Chiralpack OD-H column (95 : 5 hexane : 2-propanol), 1 mL min^−1^; 220 nm, 25 °C; *t*_R_ (major) = 24.8 min, *t*_R_ (minor) = 29.3 min. ^1^H NMR (400 MHz, DMSO): *δ* = 7.76 (s, 1H), 7.70 (dd, *J* = 12.9, 7.8 Hz, 2H), 7.54 (t, *J* = 7.7 Hz, 1H), 5.43 (s, 1H), 5.00 (d, *J* = 7.2 Hz, 1H), 2.70 (t, *J* = 10.9 Hz, 1H), 2.33 (d, *J* = 5.8 Hz, 2H), 1.93–1.69 (m, 2H), 1.69–1.43 (m, 3H), 1.22–1.08 (m, 1H) ppm. ^13^C NMR (101 MHz, DMSO): *δ* = 211.40, 145.74, 132.32, 131.27, 130.94, 129.61, 119.44, 111.30, 70.93, 57.63, 41.72, 30.03, 27.95, 23.62 ppm.

#### (2*S*,1′*R*)-2-(Hydroxy-(2-cyanophenyl)methyl)cyclohexan-1-one (4f)

Reaction time: 48 h; yield: 91%; white solid. Enantiomeric excess: 97%, determined by HPLC with a Chiralpack OD-H column (95 : 5 hexane : 2-propanol), 1 mL min^−1^; 220 nm, 25 °C; *t*_R_ (major) = 16.3 min, *t*_R_ (minor) = 18.6 min. ^1^H NMR (400 MHz, DMSO): *δ* = 7.78 (d, *J* = 7.7 Hz, 1H), 7.72 (t, *J* = 7.6 Hz, 1H), 7.63 (d, *J* = 7.9 Hz, 1H), 7.47 (t, *J* = 7.5 Hz, 1H), 5.67 (d, *J* = 4.1 Hz, 1H), 5.17 (dd, *J* = 8.8, 4.0 Hz, 1H), 2.76 (td, *J* = 9.5, 5.4 Hz, 1H), 2.38 (ddd, *J* = 17.6, 11.4, 6.3 Hz, 2H), 1.96–1.81 (m, 1H), 1.69 (ddd, *J* = 19.2, 18.5, 13.2 Hz, 2H), 1.56 (dd, *J* = 16.1, 6.6 Hz, 1H), 1.40–1.30 (m, 1H), 1.23 (dd, *J* = 19.8, 10.0 Hz, 1H) ppm. ^13^C NMR (101 MHz, DMSO): *δ* = 211.41, 148.19, 134.31, 133.40, 129.08, 129.03, 118.77, 111.23, 70.52, 58.02, 42.48, 31.27, 28.93, 24.42 ppm.

#### (2*S*,1′*R*)-2-(Hydroxy-(4-trifluoromethylphenyl)methyl)cycloh-exan-1-one (4g)

Reaction time: 48 h; yield: 94%; white solid. Enantiomeric excess: 97%, determined by HPLC with a Chiralpack AD-H column (90 : 10 hexane : 2-propanol), 1 mL min^−1^; 220 nm, 25 °C; *t*_R_ (major) = 12.8 min, *t*_R_ (minor) = 10.4 min. ^1^H NMR (400 MHz, DMSO): *δ* = 7.68 (d, *J* = 8.1 Hz, 2H), 7.56 (d, *J* = 8.1 Hz, 2H), 5.42 (d, *J* = 4.5 Hz, 1H), 5.05 (dd, *J* = 7.2, 4.6 Hz, 1H), 2.72–2.64 (m, 1H), 2.36 (t, *J* = 6.5 Hz, 2H), 1.91–1.70 (m, 2H), 1.70–1.47 (m, 3H), 1.16 (ddd, *J* = 13.2, 10.4, 6.9 Hz, 1H) ppm. ^13^C NMR (101 MHz, DMSO): *δ* = 211.67, 149.11, 128.49, 128.39, 125.45, 71.44, 58.12, 41.89, 30.28, 28.22, 23.80 ppm.

#### (2*S*,1′*R*)-2-(Hydroxy-(2,4-dichlorophenyl)methyl)cyclohexan-1-one (4h)

Reaction time: 48 h; yield: 90%; white solid. Enantiomeric excess: 98%, determined by HPLC with a Chiralpack OD-H column (95 : 5 hexane : 2-propanol), 1 mL min^−1^; 220 nm, 25 °C; *t*_R_ (major) = 9.5 min, *t*_R_ (minor) = 11.7 min. ^1^H NMR (400 MHz, DMSO): *δ* = 7.54 (d, *J* = 8.4 Hz, 2H), 7.44 (dd, *J* = 8.5, 1.4 Hz, 1H), 5.48 (d, *J* = 4.6 Hz, 1H), 5.25 (dd, *J* = 8.4, 4.6 Hz, 1H), 2.71–2.61 (m, 1H), 2.42–2.30 (m, 2H), 1.92–1.32 (m, 6H) ppm. ^13^C NMR (101 MHz, DMSO): *δ* = 211.07, 132.91, 132.65, 130.93, 128.52, 128.09, 67.89, 57.67, 42.13, 30.70, 28.37, 24.11 ppm.

#### (2*S*,1′*R*)-2-(Hydroxy-(4-chlorophenyl)methyl)cyclohexan-1-one (4i)

Reaction time: 48 h; yield: 57%; white solid. Enantiomeric excess: 98%, determined by HPLC with a Chiralpack OD-H column (95 : 5 hexane : 2-propanol), 1 mL min^−1^; 220 nm, 25 °C; *t*_R_ (major) = 12.0 min, *t*_R_ (minor) = 16.3 min. ^1^H NMR (400 MHz, DMSO): *δ* = 7.41–7.31 (m, 4H), 5.29 (d, *J* = 4.4 Hz, 1H), 4.96 (dd, *J* = 7.7, 4.4 Hz, 1H), 2.61 (td, *J* = 8.9, 5.3 Hz, 1H), 2.41–2.28 (m, 2H), 1.89–1.70 (m, 2H), 1.64 (ddd, *J* = 12.4, 5.8, 3.4 Hz, 1H), 1.57–1.45 (m, 2H), 1.21–1.08 (m, 1H) ppm. ^13^C NMR (101 MHz, DMSO): *δ* = 212.13, 143.47, 132.40, 129.67, 128.76, 71.58, 58.49, 41.99, 30.49, 28.50, 23.86 ppm.

#### (2*S*,1′*R*)-2-(Hydroxy-(3-chlorophenyl)methyl)cyclohexan-1-one (4j)

Reaction time: 48 h; yield: 54%; white solid. Enantiomeric excess: 98%, determined by HPLC with a Chiralpack AD-H column (95 : 5 hexane : 2-propanol), 1 mL min^−1^; 220 nm, 25 °C; *t*_R_ (major) = 17.1 min, *t*_R_ (minor) = 15.1 min. ^1^H NMR (400 MHz, DMSO): *δ* = 7.37–7.23 (m, 4H), 5.31 (d, *J* = 4.5 Hz, 1H), 4.92 (dd, *J* = 7.6, 4.5 Hz, 1H), 2.69–2.55 (m, 1H), 2.37–2.27 (m, 2H), 1.85–1.68 (m, 2H), 1.66–1.56 (m, 1H), 1.55–1.42 (m, 2H), 1.20–1.07 (m, 1H) ppm. ^13^C NMR (101 MHz, DMSO): *δ* = 211.80, 146.95, 133.41, 130.47, 127.65, 127.39, 126.34, 71.45, 58.14, 41.86, 30.36, 28.29, 23.74 ppm.

#### (2*S* 1′*R*)-2-(Hydroxy-(2-chlorophenyl)methyl)cyclohexan-1-one (4k)

Reaction time: 48 h; yield: 59%; white solid. Enantiomeric excess: 99%, determined by HPLC with a Chiralpack OD-H column (95 : 5 hexane : 2-propanol), 1 mL min^−1^; 220 nm, 25 °C; *t*_R_ (major) = 9.7 min, *t*_R_ (minor) = 6.0 min. ^1^H NMR (400 MHz, DMSO): *δ* = 7.54 (d, *J* = 7.6 Hz, 1H), 7.37 (t, *J* = 8.7 Hz, 2H), 7.27 (t, *J* = 7.6 Hz, 1H), 5.38 (d, *J* = 3.8 Hz, 1H), 5.32 (d, *J* = 8.7 Hz, 1H), 2.75–2.60 (m, 1H), 2.37 (t, *J* = 6.5 Hz, 2H), 1.85 (dd, *J* = 11.2, 5.7 Hz, 1H), 1.71 (dt, *J* = 12.3, 9.9 Hz, 2H), 1.53 (dd, *J* = 9.0, 3.6 Hz, 1H), 1.45–1.27 (m, 2H) ppm. ^13^C NMR (101 MHz, DMSO): *δ* = 211.62, 142.03, 132.44, 129.69, 129.43, 129.41, 128.14, 68.42, 58.31, 42.30, 30.93, 28.73, 24.30 ppm.

#### (2*S*,1′*R*)-2-(Hydroxy-(4-bromophenyl)methyl)cyclohexan-1-one (4l)

Reaction time: 48 h; yield: 50%; white solid. Enantiomeric excess: 99%, determined by HPLC with a Chiralpack AD-H column (90 : 10 hexane : 2-propanol), 1 mL min^−1^; 220 nm, 25 °C; *t*_R_ (major) = 16.7 min, *t*_R_ (minor) = 14.4 min. ^1^H NMR (400 MHz, DMSO): *δ* = 7.47 (d, *J* = 7.8 Hz, 2H), 7.26 (d, *J* = 7.7 Hz, 2H), 5.24 (s, 1H), 4.90 (d, *J* = 7.7 Hz, 1H), 3.35 (s, 1H), 2.39–2.22 (m, 2H), 1.74 (dd, *J* = 31.9, 5.4 Hz, 2H), 1.61 (s, 1H), 1.48 (d, *J* = 9.3 Hz, 2H), 1.12 (dd, *J* = 12.1, 6.9 Hz, 1H) ppm. ^13^C NMR (101 MHz, DMSO): *δ* = 211.90, 143.70, 131.47, 129.85, 120.73, 71.42, 58.24, 41.79, 30.28, 28.28, 23.67 ppm.

#### (2*S*,1′*R*)-2-(Hydroxy-(2-bromophenyl)methyl)cyclohexan-1-one (4m)

Reaction time: 48 h; yield: 55%; white solid. Enantiomeric excess: 97%, determined by HPLC with a Chiralpack AD-H column (90 : 10 hexane : 2-propanol), 1 mL min^−1^; 220 nm, 25 °C; *t*_R_ (major) = 11.0 min, *t*_R_ (minor) = 12.8 min. ^1^H NMR (400 MHz, DMSO): *δ* = 7.53 (ddd, *J* = 11.6, 7.9, 1.3 Hz, 2H), 7.40 (dd, *J* = 11.0, 3.9 Hz, 1H), 7.19 (td, *J* = 8.0, 1.7 Hz, 1H), 5.41 (s, 1H), 5.27 (d, *J* = 8.7 Hz, 1H), 2.68 (dd, *J* = 15.7, 8.1 Hz, 1H), 2.37 (dd, *J* = 10.6, 5.4 Hz, 2H), 1.86 (dd, *J* = 11.5, 5.3 Hz, 1H), 1.80–1.61 (m, 2H), 1.60–1.45 (m, 1H), 1.41–1.30 (m, 2H) ppm. ^13^C NMR (101 MHz, DMSO): *δ* = 211.74, 143.84, 132.88, 130.14, 129.98, 128.88, 123.37, 71.07, 58.58, 42.61, 31.29, 28.93, 24.65 ppm.

#### (2*S*,1′*R*)-2-(Hydroxy-(4-fluorophenyl)methyl)cyclohexan-1-one (4n)

Reaction time: 48 h; yield: 46%; white solid. Enantiomeric excess: 95%, determined by HPLC with a Chiralpack OD-H column (95 : 5 hexane : 2-propanol), 1 mL min^−1^; 220 nm, 25 °C; *t*_R_ (major) = 13.7 min, *t*_R_ (minor) = 12.6 min. ^1^H NMR (400 MHz, DMSO) *δ* = 7.39 (ddd, *J* = 11.4, 8.5, 5.8 Hz, 1H), 7.20–7.10 (m, 1H), 5.24 (d, *J* = 19.1 Hz, 1H), 5.06 (dd, *J* = 54.9, 6.3 Hz, 1H), 2.61 (ddd, *J* = 15.6, 9.5, 5.2 Hz, 1H), 2.47–2.26 (m, 1H), 1.96–1.49 (m, 3H) ppm. ^13^C NMR (101 MHz, DMSO) *δ* = 212.33, 163.39, 140.65, 129.71, 129.63, 115.63, 115.23, 71.68, 57.85, 41.93, 30.56, 28.57, 24.37, 23.76 ppm.

#### (2*S*,1′*R*)-2-(Hydroxy(phenyl)methyl)cyclohexan-1-one (4o)

Reaction time: 48 h; yield: 49%; white solid. Enantiomeric excess: 97%, determined by HPLC with a Chiralpack OD-H column (95 : 5 hexane : 2-propanol), 1 mL min^−1^; 220 nm, 25 °C; *t*_R_ (major) = 11.4 min, *t*_R_ (minor) = 14.7 min. ^1^H NMR (400 MHz, DMSO): *δ* = 7.35–7.22 (m, 5H), 5.19 (d, *J* = 4.3 Hz, 1H), 4.93 (dd, *J* = 8.3, 4.3 Hz, 1H), 2.60 (td, *J* = 8.4, 5.4 Hz, 1H), 2.45–2.23 (m, 2H), 1.83–1.64 (m, 3H), 1.56–1.43 (m, 2H), 1.18 (ddd, *J* = 12.5, 8.8, 4.0 Hz, 1H) ppm. ^13^C NMR (101 MHz, DMSO): *δ* = 212.49, 144.45, 128.86, 128.03, 127.82, 72.50, 58.73, 41.87, 30.70, 28.66, 23.68 ppm.

#### (2*S*,1′*R*)-2-(Hydroxy(phenyl)methyl)cyclohexan-1-one (4p)

Reaction time: 48 h; yield: 36%; white solid. Enantiomeric excess: 97%, determined by HPLC with a Chiralpack AD-H column (90 : 10 hexane : 2-propanol), 1 mL min^−1^; 220 nm, 25 °C; *t*_R_ (major) = 14.7 min, *t*_R_ (minor) = 17.0 min. ^1^H NMR (400 MHz, DMSO): *δ* = 7.92–7.86 (m, 1H), 7.83 (s, 1H), 7.55–7.46 (m, 1H), 5.11 (d, *J* = 8.2 Hz, 1H), 2.72 (td, *J* = 8.6, 5.4 Hz, 1H), 2.48–2.30 (m, 1H), 1.84–1.71 (m, 2H), 1.65 (ddd, *J* = 6.8, 4.9, 2.6 Hz, 1H), 1.50 (ddd, *J* = 13.4, 7.7, 4.4 Hz, 1H) ppm. ^13^C NMR (101 MHz, DMSO): *δ* = 212.45, 141.98, 133.59, 133.35, 128.69, 128.48, 128.43, 126.97, 126.61, 126.45, 126.09, 124.69, 72.62, 58.57, 42.28, 30.85, 28.64, 27.40, 25.25, 23.81 ppm.

#### (2*S*,1*′R*)-2-(Hydroxy-(4-nitro-phenyl)methyl)cyclopentan-1-one (4t)

Reaction time: 48 h; yield: 83%; light yellow solid. Enantiomeric excess: 64%, determined by HPLC with a Chiralpack AS-H column (70 : 30 hexane : 2-propanol), 1 mL min^−1^; 254 nm, 25 °C; *t*_R_ (major) = 11.2 min, *t*_R_ (minor) = 23.2 min. ^1^H NMR (400 MHz, DMSO) *δ* = 8.19 (t, *J* = 6.9 Hz, 2H), 7.58 (dd, *J* = 37.9, 8.2 Hz, 2H), 5.72 (s, 1H), 5.19 (s, 1H), 2.45 (s, 1H), 2.30–2.19 (m, 1H), 2.14–2.00 (m, 1H), 1.91 (dd, *J* = 12.7, 8.2 Hz, 1H), 1.80–1.51 (m, 3H) ppm. ^13^C NMR (101 MHz, DMSO) *δ* = 218.70, 153.93, 147.27, 127.77, 124.15, 72.01, 55.96, 26.24, 22.86, 21.08 ppm.

#### (2*S*,1′*R*)-4-Hydroxy-4-(4-nitrophenyl)pentan-3-one (4u)

Reaction time: 48 h; yield: 88%; light yellow solid. Enantiomeric excess: 75%, determined by HPLC with a Chiralpack AS-H column (70 : 30 hexane : 2-propanol), 1 mL min^−1^; 254 nm, 25 °C; *t*_R_ (major) = 10.4 min, *t*_R_ (minor) = 11.0 min. ^1^H NMR (400 MHz, DMSO) *δ* = 8.21 (dd, *J* = 11.3, 5.0 Hz, 1H), 7.64 (dd, *J* = 6.4, 2.3 Hz, 1H), 5.85–5.69 (m, 1H), 5.25–4.73 (m, 1H), 2.83–2.72 (m, 1H), 2.49 (dd, *J* = 14.5, 6.0 Hz, 1H), 2.19 (d, *J* = 15.3 Hz, 1H), 0.92 (t, *J* = 7.2 Hz, 1H), 0.83 (d, *J* = 6.9 Hz, 1H), 0.72 (d, *J* = 7.0 Hz, 1H) ppm. ^13^C NMR (101 MHz, DMSO) *δ* = 209.51, 152.37, 147.69, 127.91, 124.19, 72.52, 52.17, 36.74, 8.34 ppm.

#### (2*S*,1′*R*)-4-Hydroxy-4-(4-nitrophenyl)butan-2-one (4v)

Reaction time: 48 h; yield: 95%; light yellow solid. Enantiomeric excess: 22%, determined by HPLC with a Chiralpack AS-H column (70 : 30 hexane : 2-propanol), 1 mL min^−1^; 254 nm, 25 °C; *t*_R_ (major) = 12.8 min, *t*_R_ (minor) = 15.5 min. ^1^H NMR (400 MHz, DMSO): *δ* = 8.22 (t, *J* = 14.5 Hz, 2H), 7.65 (d, *J* = 8.2 Hz, 2H), 5.72 (d, *J* = 4.7 Hz, 1H), 5.16 (dd, *J* = 11.6, 5.9 Hz, 1H), 2.76 (d, *J* = 6.5 Hz, 2H), 2.15 (s, 3H) ppm. ^13^C NMR (101 MHz, DMSO): *δ* = 206.77, 153.70, 146.98, 127.49, 123.83, 68.57, 52.84, 30.87 ppm.

## Conflicts of interest

There are no conflicts to declare.

## Supplementary Material

RA-008-C8RA04802A-s001
