# Supplementary material for: Preparation of prolinamide with adamantane for aldol reaction catalysis in brine and separation using a poly(AN-MA-β-CD) nanofibrous film via host–guest interaction
Source: RSC Adv. 2018 Aug 7;8(50):28376–85. doi: 10.1039/c8ra04802a (PMC9084239; doi:10.1039/c8ra04802a)
Supplement: RA-008-C8RA04802A-s001 [file RA-008-C8RA04802A-s001.pdf]

Electronic Supplementary Material (ESI) for RSC Advances.
This journal is © The Royal Society of Chemistry 2018

Electronic Supplementary Information (ESI)

Preparation of prolinamide with adamantane for aldol reaction catalysis in brine and separation by poly(AN-MA-β-CD) nanofibrous film via host–guest interaction

Rui Wang,[a] Enjie Xu,[a] Zhenming Su,[b] Haifeng Duan,[a] Jinjin Wang,[a] Longqi Xue,[a] Yingjie Lin,[a] Yaoxian Li,[a] Zhonglin Wei,*[,a] and Qingbiao Yang*[,a]

a. College of Chemistry, Jilin University, 2699 Qianjin Street, Changchun 130012, P. R. China.

b. Security Check, Jilin Province, 566B Guigu Street, Changchun 130012, P. R. China.

Corresponding authors e-mails: yangqb@jlu.edu.cn (Qingbiao Yang), zlwei@jlu.edu.cn (Zhonglin Wei)

Supporting Information

Table of Contents

1. Experimental section S2

2. The ^{1}H NMR, ^{13}C NMR and HRMS spectra of catalyst S4

3. The ^{1}H NMR and ^{13}C NMR spectra of products S12

4. The HPLC of the aldol products S31

5. References S50

1. Experimental section

1.1 Larger-scale reactions between cyclohexanone and aldehydes

A larger-scale asymmetric aldol reactions were performed with 4-nitrobenzaldehyde and cyclohexanone in brine at 0 ℃ with the catalyst loading of 10 mol % being used. The larger-scale experiments proceeded smoothly using the same procedure as for the experimental scale reactions. As can be seen from the results summarized in Table S1, the enantioselectivities of large-scale asymmetric aldol reactions were reduced because a lot of 4-nitrobenzaldehyde were not easy to dissolve in brine under unevenly stringing. Enantioselectivity may remain unchanged when large mixing slurry was used in industry.

Table S1. Large-scale asymmetric aldol reactions of 4-nitrobenzaldehyde and cyclohexanone.

Entry	4-nitrobenzaldehyde (g)	Yield (%)	dr (anti/syn)	*ee* (%)
1	0.5	97	99:1	84
2	1.0	97	98:2	84

1.2 UV analysis

The standard curve of catalyst in different rations of MeOH and distilled water, and pure MeOH were shown in Figure S1. The absorption intensity at 278 nm was significantly under mixture solvent. But linear R^2 of 1/3 was only 0.9996 because catalyst did not completely dissolved in this mixture solvent. However, wavelength in pure MeOH was shifted to 243 nm due to without water.

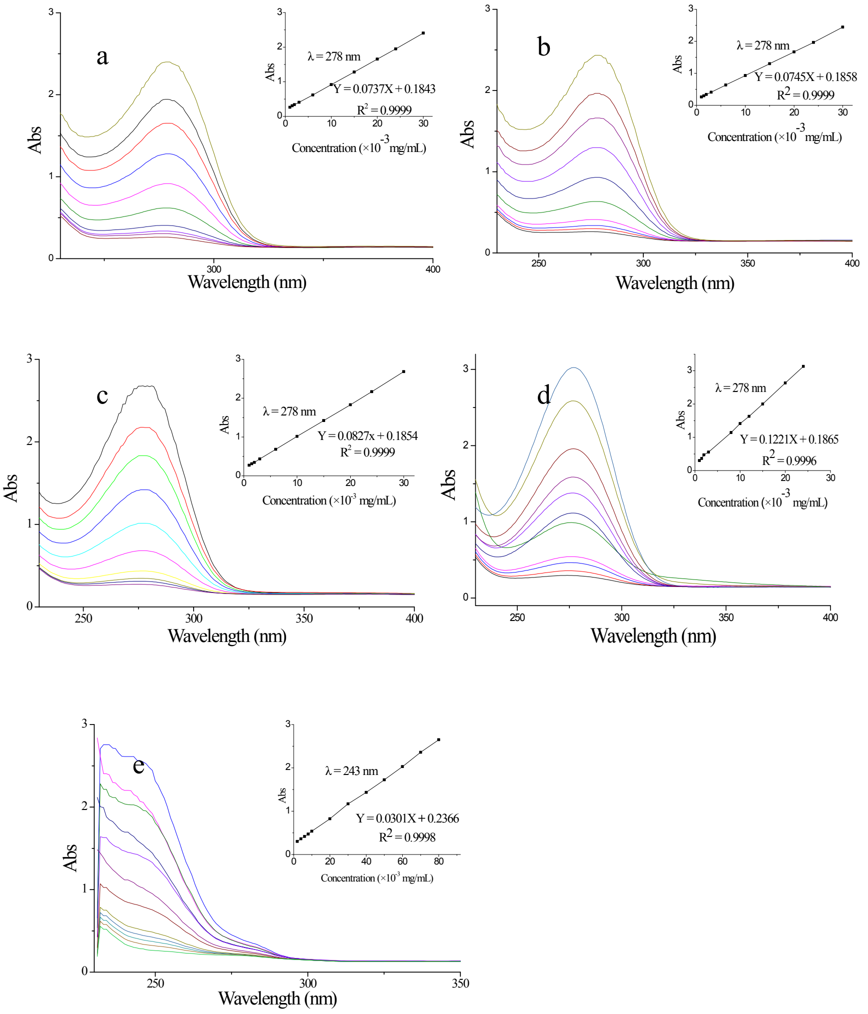

Figure S1 Standard curve of catalyst in solvent. Ratio between MeOH and H_2O 2:1 (a), 1:1 (b), 1:2 (c), 1:3 (d), and pure MeOH (e).

1.3 Optimizing solvent and number of adsorption

The catalyst (0.05 mmol) was dissolved in brine (1.0 mL) with 0.5 mL of cyclohexanone to simulate the system of aldol reaction. A mixture (15 mL) of MeOH and distilled water, and 0.2 g of the nanofibrous membrane were then added to the system. The catalyst was compelled into β-CD cavity under ultrasound due to its insolubility in water. After the membrane was filtered, 0.2 g of new membrane was added and then ultrasound was continued for 10 min; this process was repeated for several times.

The adsorption rate of catalyst provided by different ratios of MeOH and H_2O are shown in Table S2. The adsorption rate increased with the increase in volume of H_2O in the mixture solvent. When the ratio of MeOH and H_2O was beyond 1:2, the adsorption rate was not obviously improved. The optimal MeOH/H_2O ratio was found to be 1:2 (98.5% adsorption rate). The effect of duration on adsorption rate is shown in Table S3. Compared with the fifth cycle, the adsorption rate at the sixth cycle was not further increased. The catalyst was then washed with methanol under ultrasound, and the process was repeated for another three times. The separation rate of catalyst from the fibrous membrane was 97.7%, and the total recovery rate was 96.2%.

Table S2. The effect ratios of MeOH and H_2O on adsorption rate[a]

Entry	MeOH : H_2O[b] (v:v)	Adsorption rate (%)
1	2:1	97.2
2	1:1	97.6
3	1:2	98.5
4	1:3	98.6

[a] Total volume of MeOH and H_2O was 15 mL; [b]H_2O : distilled water.

Table S3. The effect times on adsorption rate

Entry	Adsorption rate (%)
1	62.0
2	79.4
3	90.8
4	97.2
5	98.5
6	98.5

2. The ^{1}H NMR, ^{13}C NMR and HRMS spectra of catalyst

^{1}H NMR spectra of 1a

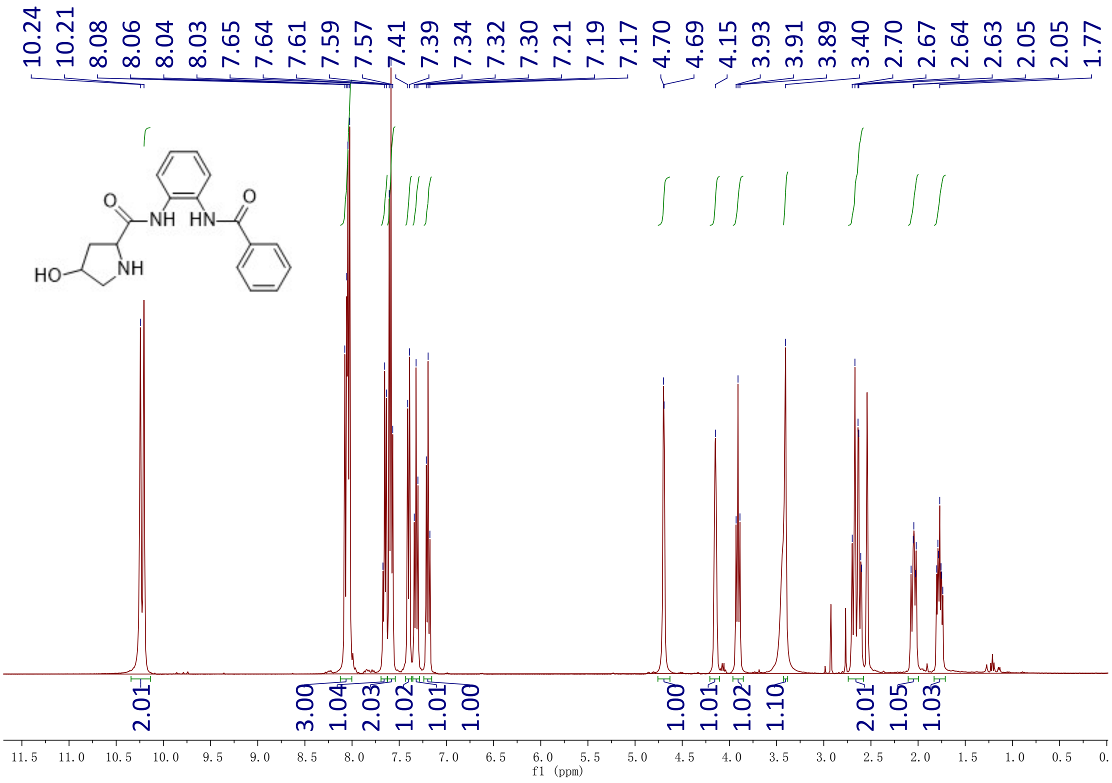

^{13}C NMR spectra of 1a

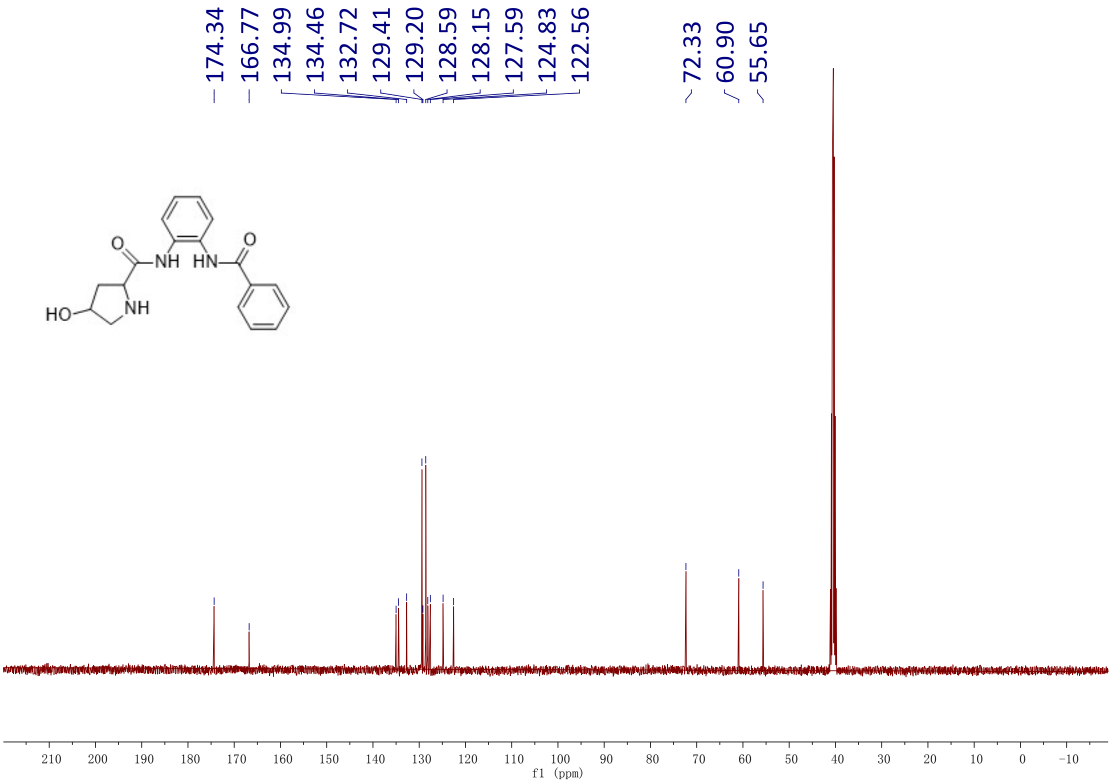

HRMS spectra of 1a

Mass Spectrum SmartFormula Report

Analysis Info

Analysis Name	C:\Users\lenovo\Desktop\ÐÂ½¨ÎÄ¼þ¼Ð (2)\yqb 1_P1-A-1_01_3835.d	Acquisition Date	10/26 ÐÇÆÚËÄ 16:42:10
Method	lc-ms-hr-low.m	Operator	zlwei
Sample Name	yqb 1	Instrument / Ser#	micrOTOF-Q II 10351
Comment			

Acquisition Parameter

Source Type	ESI	Ion Polarity	Positive	Set Nebulizer	0.4 Bar
Focus	Active	Set Capillary	4500 V	Set Dry Heater	200 °C
Scan Begin	50 m/z	Set End Plate Offset	-500 V	Set Dry Gas	5.0 l/min
Scan End	1200 m/z	Set Collision Cell RF	100.0 Vpp	Set Divert Valve	Waste

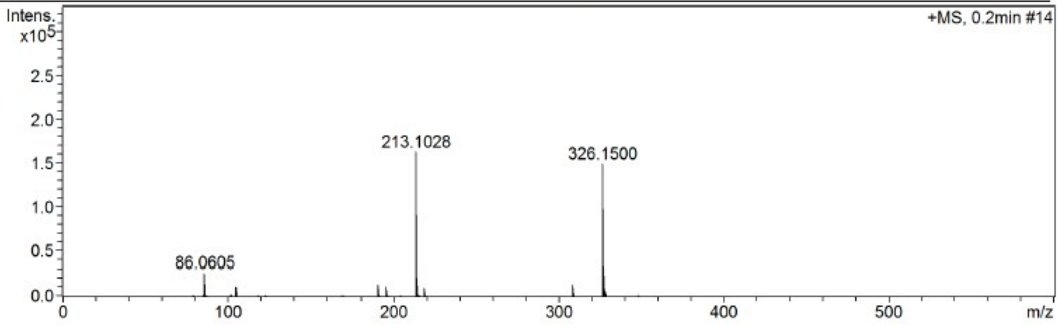

Meas. m/z	Formula	m/z	err [ppm]	Mean err [ppm]	mSigma	rdb	e¡¥Conf	N-Rule
326.1500								
	C 18 H 20 N 3 O 3	326.1499	-0.3	-0.3	11.33	10.5	even	ok

^{1}H NMR spectra of 1b

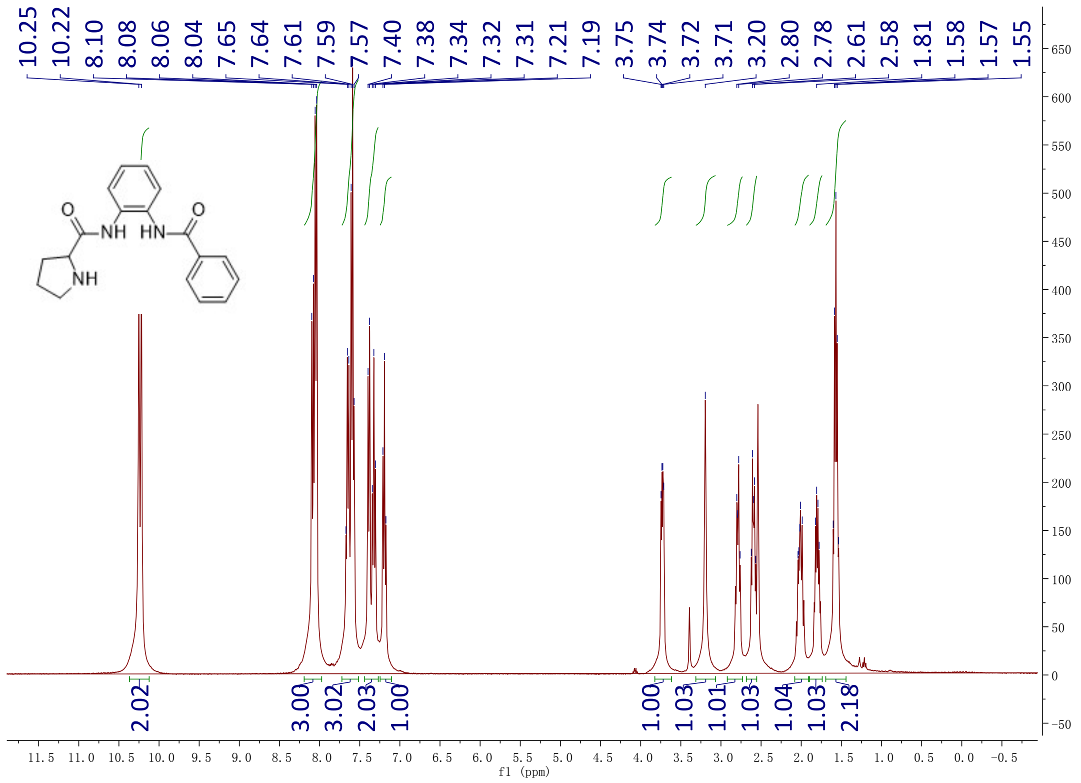

^{13}C NMR spectra of 1b

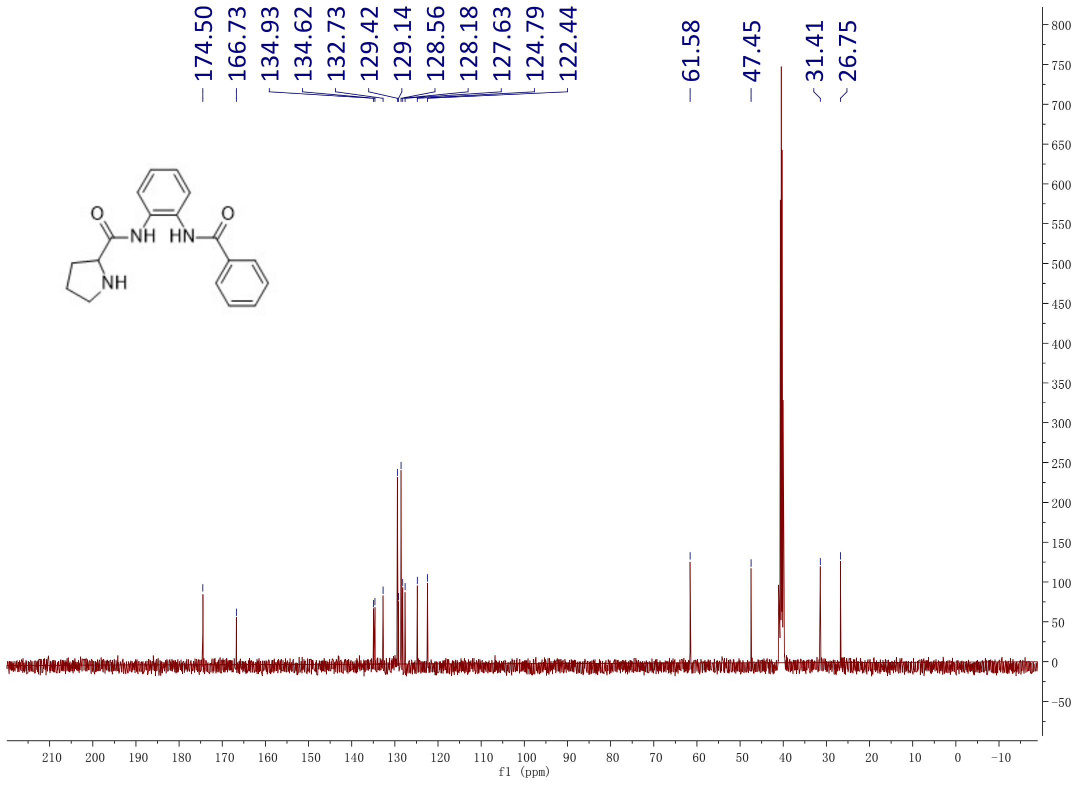

HRMS spectra of 1b

Mass Spectrum SmartFormula Report

Analysis Info

Analysis Name	C:\Users\lenovo\Desktop\ĐÂ½¨ÎÄ¼þ¼Đ (2)\yqb 2_P1-A-2_01_3836.d	Acquisition Date	10/26 ĐÇÆÚËÄ 16:48:35
Method	lc-ms-hr-low.m	Operator	zlwei
Sample Name	yqb 2	Instrument / Ser#	micrOTOF-Q II 10351
Comment			

Acquisition Parameter

Source Type	ESI	Ion Polarity	Positive	Set Nebulizer	0.4 Bar
Focus	Active	Set Capillary	4500 V	Set Dry Heater	200 °C
Scan Begin	50 m/z	Set End Plate Offset	-500 V	Set Dry Gas	5.0 l/min
Scan End	1200 m/z	Set Collision Cell RF	100.0 Vpp	Set Divert Valve	Waste

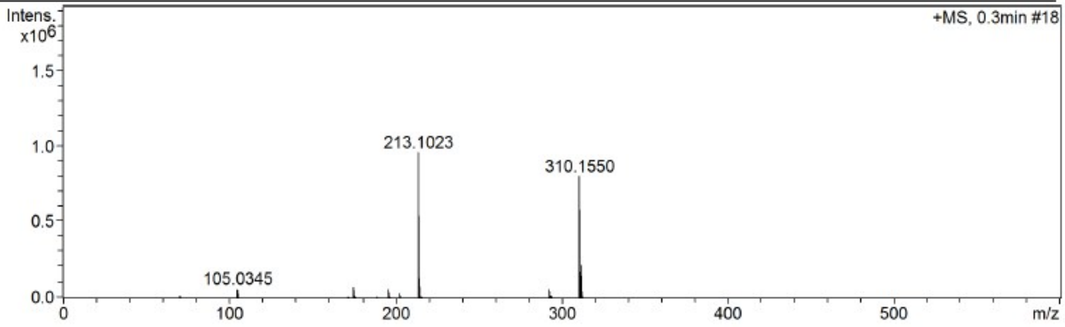

Meas. m/z	Formula	m/z	err [ppm]	Mean err [ppm]	mSigma	rdb	e¡¥Conf	N-Rule
310.1550								
	C 18 H 20 N 3 O 2	310.1550	0.2	0.5	32.08	10.5	even	ok

^{1}H NMR spectra of 1c

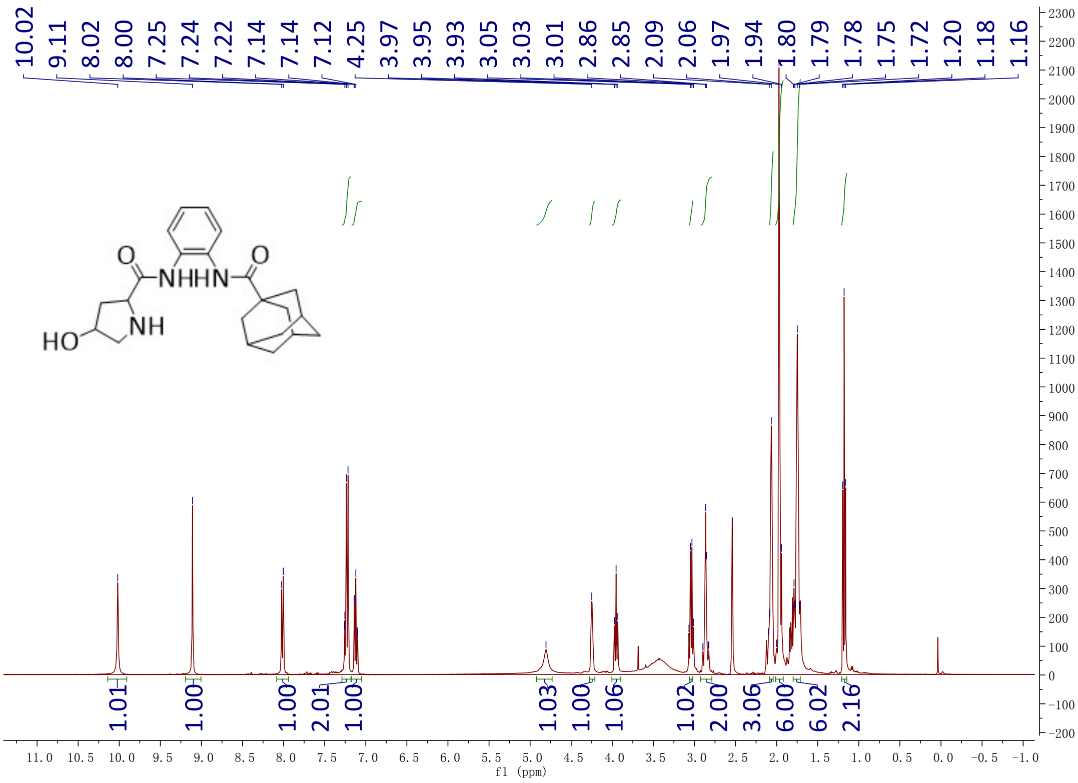

^{13}C NMR spectra of 1c

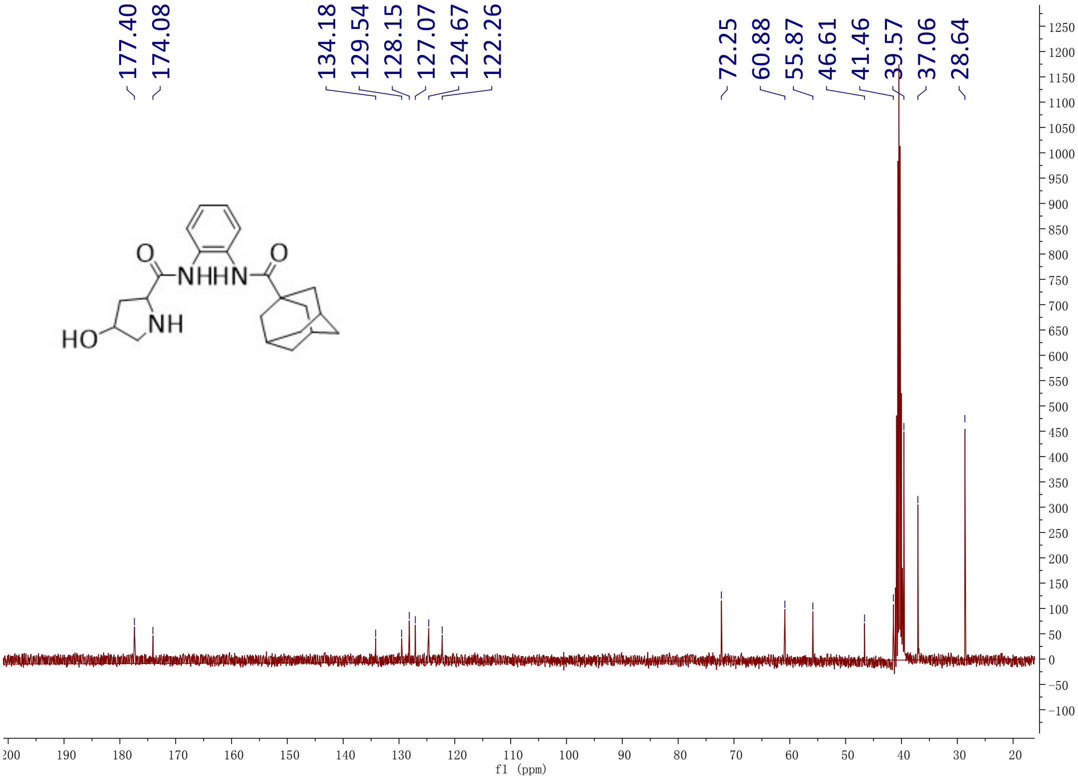

HRMS spectra of 1c

Mass Spectrum SmartFormula Report

Analysis Info

Analysis Name	C:\Users\lenovo\Desktop\ĐÂ½¨ÎÄ¼þ¼Ð (2)\yqb 3_P1-A-3_01_3837.d	Acquisition Date	10/26 ĐÇÆÚËÄ 16:55:01
Method	lc-ms-hr-low.m	Operator	zlwei
Sample Name	yqb 3	Instrument / Ser#	micrOTOF-Q II 10351
Comment			

Acquisition Parameter

Source Type	ESI	Ion Polarity	Positive	Set Nebulizer	0.4 Bar
Focus	Active	Set Capillary	4500 V	Set Dry Heater	200 °C
Scan Begin	50 m/z	Set End Plate Offset	-500 V	Set Dry Gas	5.0 l/min
Scan End	1200 m/z	Set Collision Cell RF	100.0 Vpp	Set Divert Valve	Waste

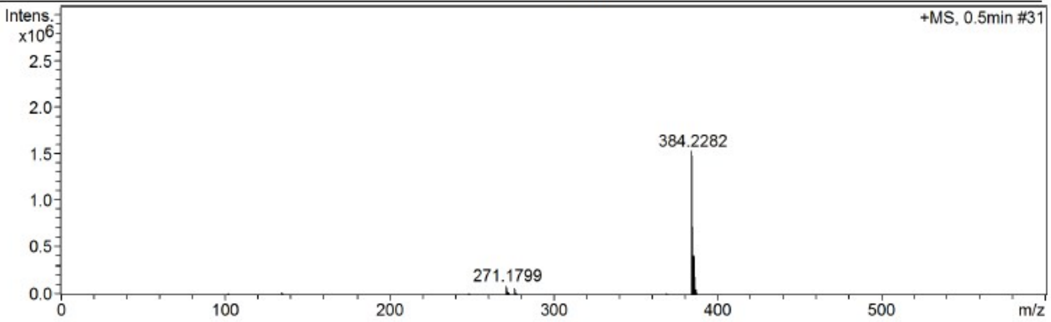

Meas. m/z	Formula	m/z	err [ppm]	Mean err [ppm]	mSigma	rdb	e¡¥Conf	N-Rule
384.2282								
	C 22 H 30 N 3 O 3	384.2282	-0.1	1.7	9.21	9.5	even	ok

^{1}H NMR spectra of 1d

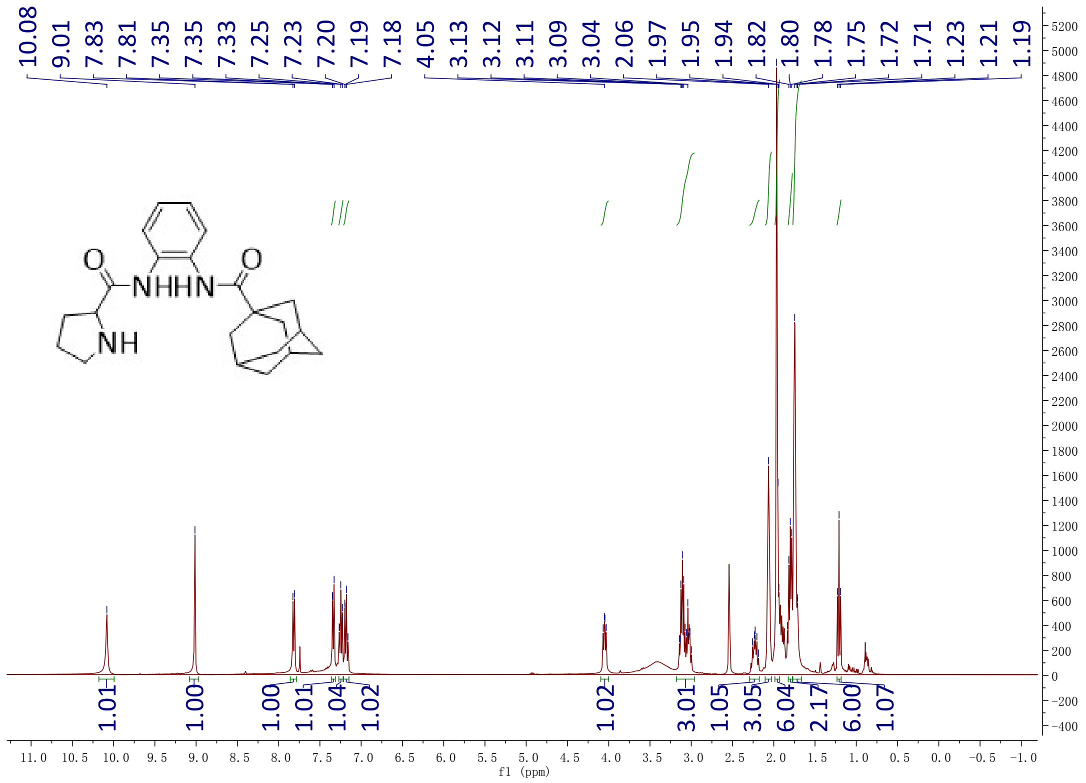

^{13}C NMR spectra of 1d

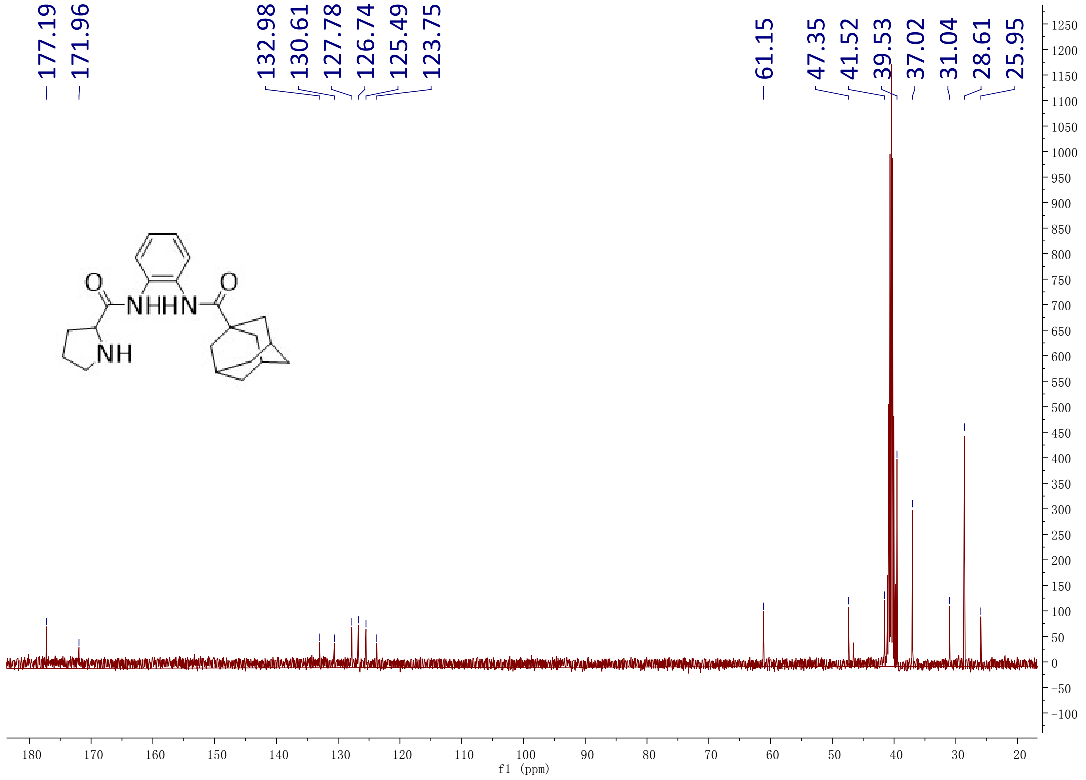

HRMS spectra of 1d

Mass Spectrum SmartFormula Report

Analysis Info

Analysis Name	C:\Users\lenovo\Desktop\ĐÂ½¨ÎÄ¼þ¼Đ (2)\yqb 4_P1-A-4_01_3838.d	Acquisition Date	10/26 ĐÇÆÚËÄ 17:01:27
Method	lc-ms-hr-low.m	Operator	zlwei
Sample Name	yqb 4	Instrument / Ser#	micrOTOF-Q II 10351
Comment			

Acquisition Parameter

Source Type	ESI	Ion Polarity	Positive	Set Nebulizer	0.4 Bar
Focus	Active	Set Capillary	4500 V	Set Dry Heater	200 °C
Scan Begin	50 m/z	Set End Plate Offset	-500 V	Set Dry Gas	5.0 l/min
Scan End	1200 m/z	Set Collision Cell RF	100.0 Vpp	Set Divert Valve	Waste

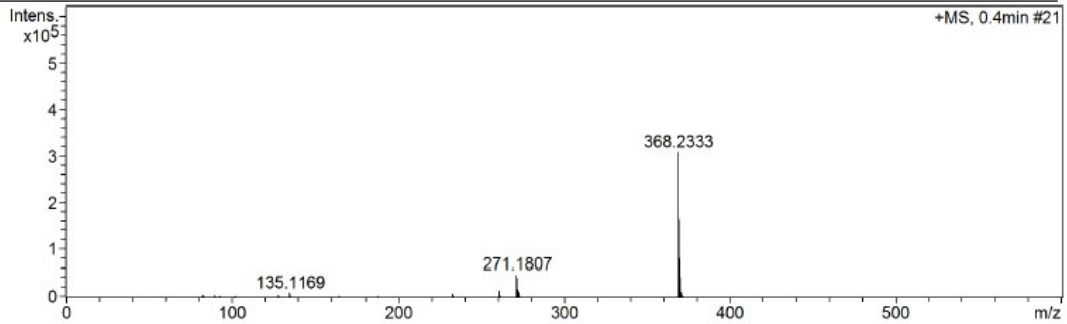

Meas. m/z	Formula	m/z	err [ppm]	Mean err [ppm]	mSigma	rdb	e¡¥Conf	N-Rule
368.2333								
	C 22 H 30 N 3 O 2	368.2333	-0.1	0.5	10.20	9.5	even	ok

3. The ^{1}H NMR and ^{13}C NMR spectra of products[1-6]

^{1}H NMR spectra of 4a

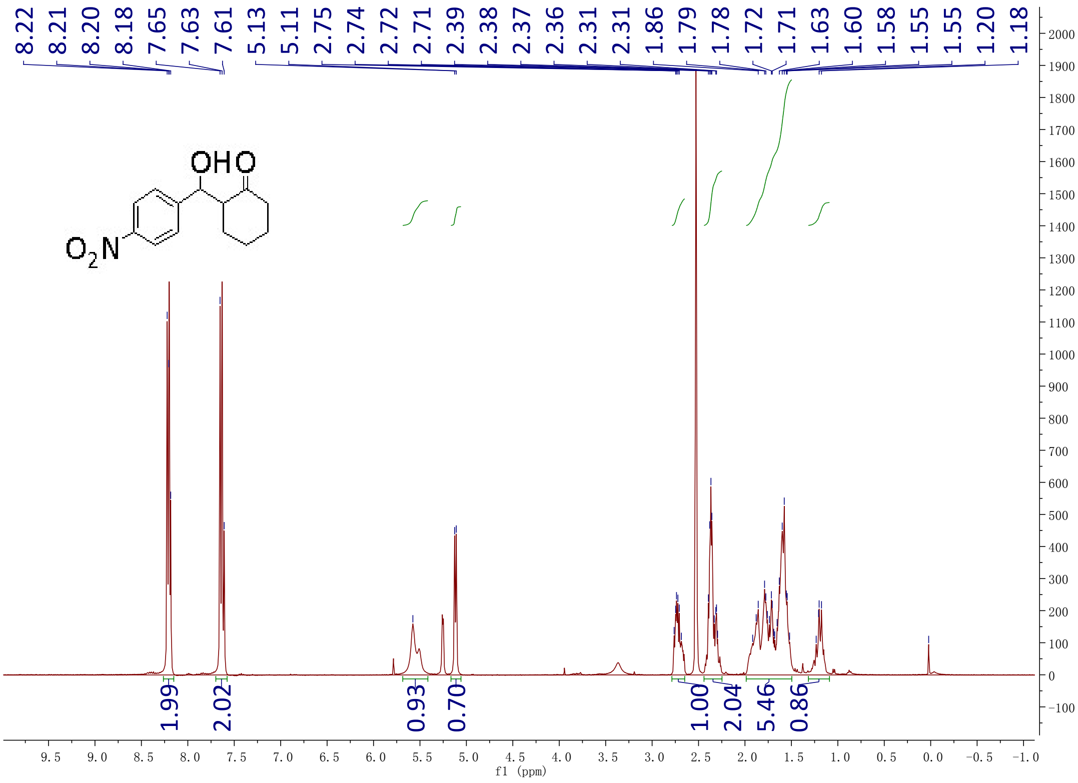

^{13}C NMR spectra of 4a

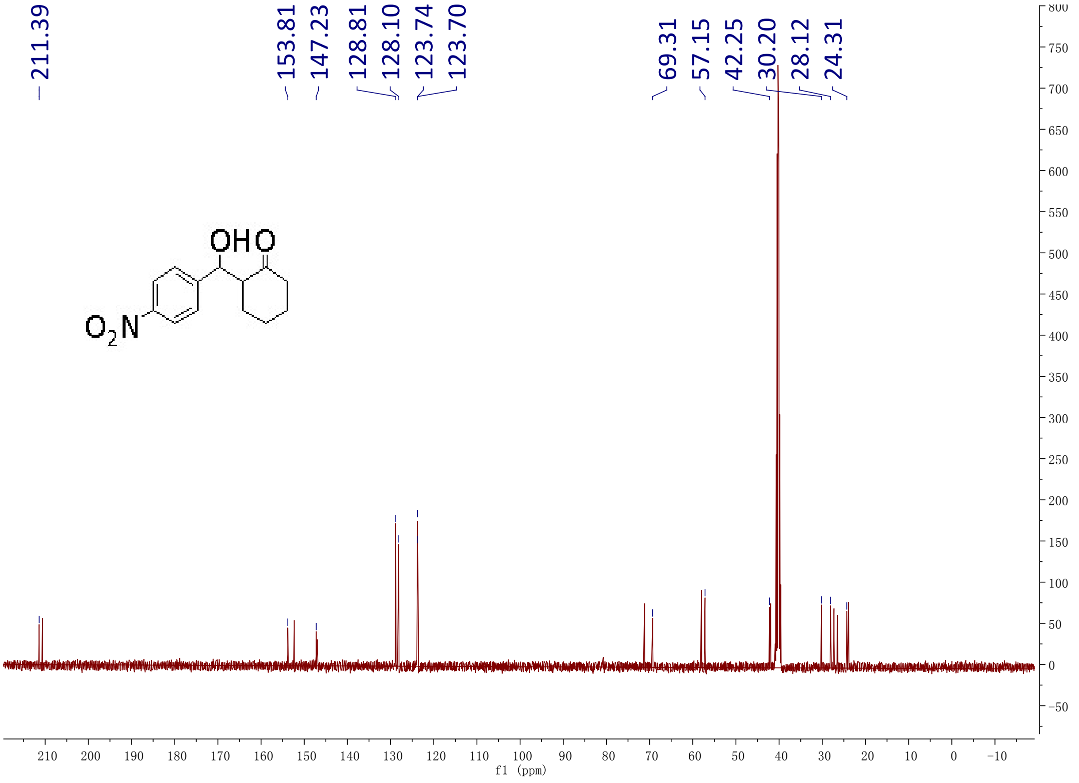

^{1}H NMR spectra of 4b

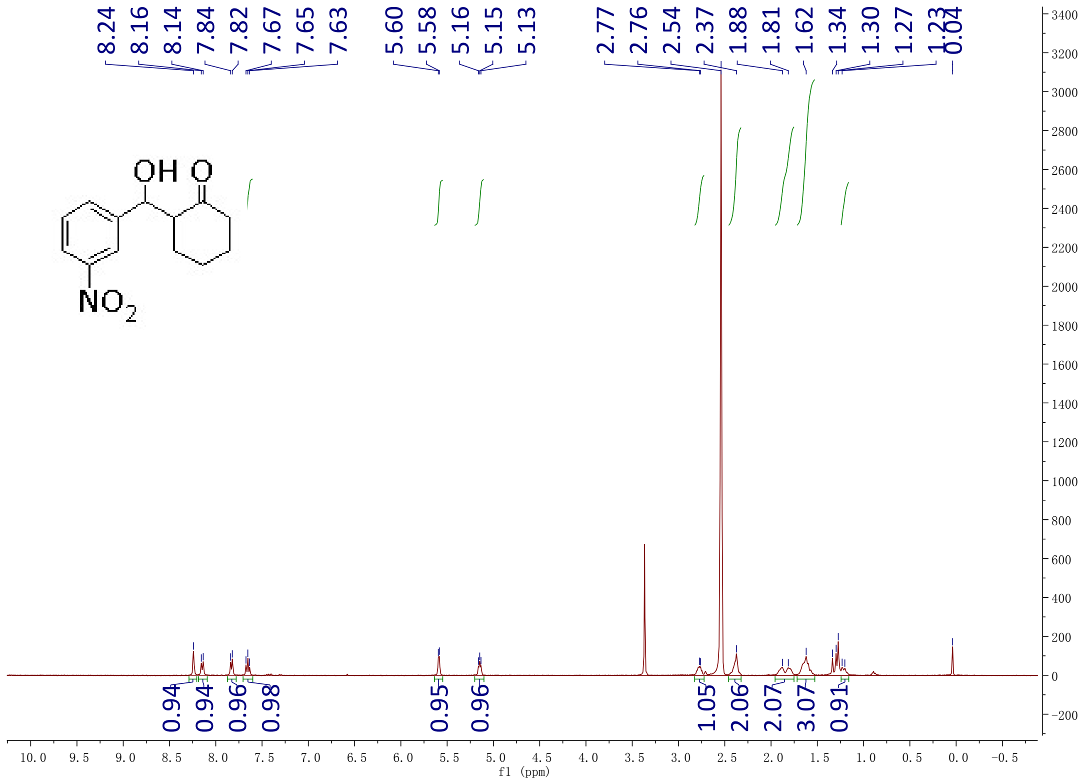

^{13}C NMR spectra of 4b

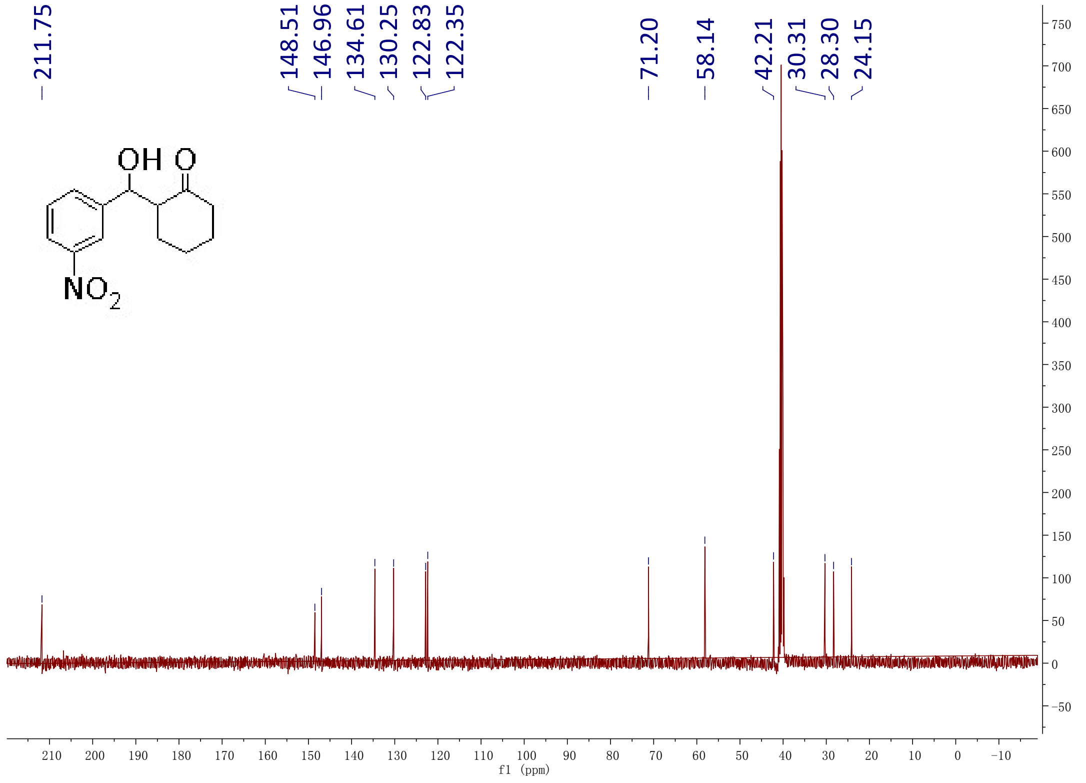

^{1}H NMR spectra of 4c

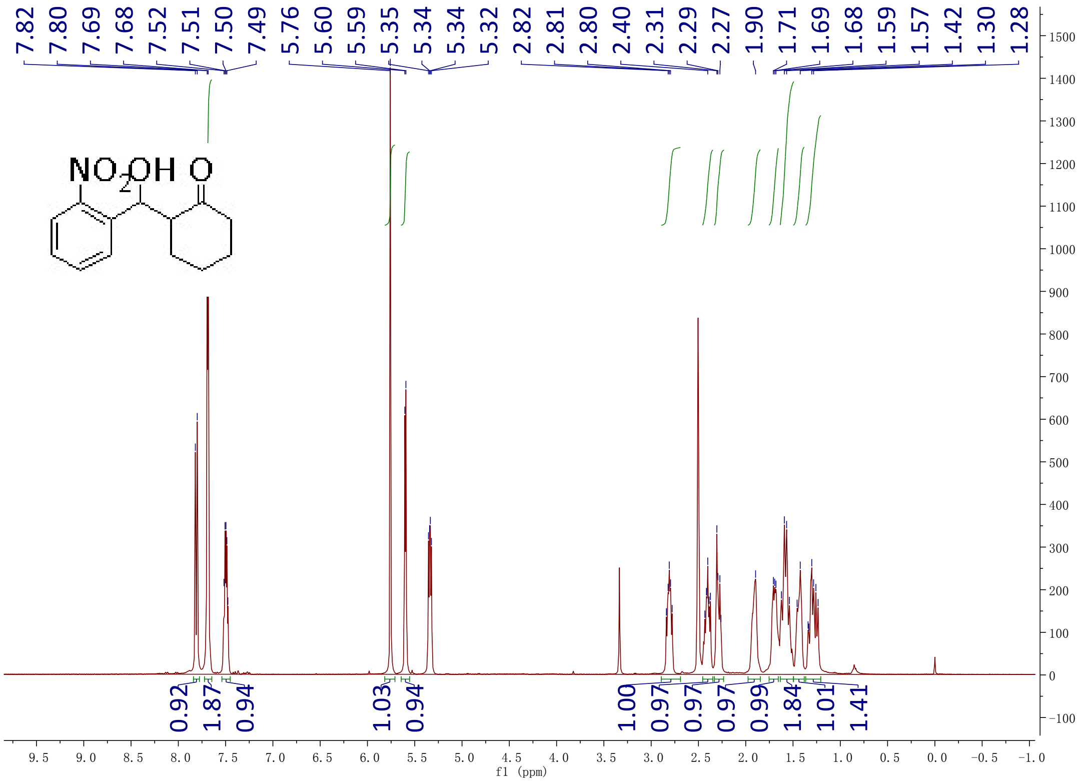

^{13}C NMR spectra of 4c

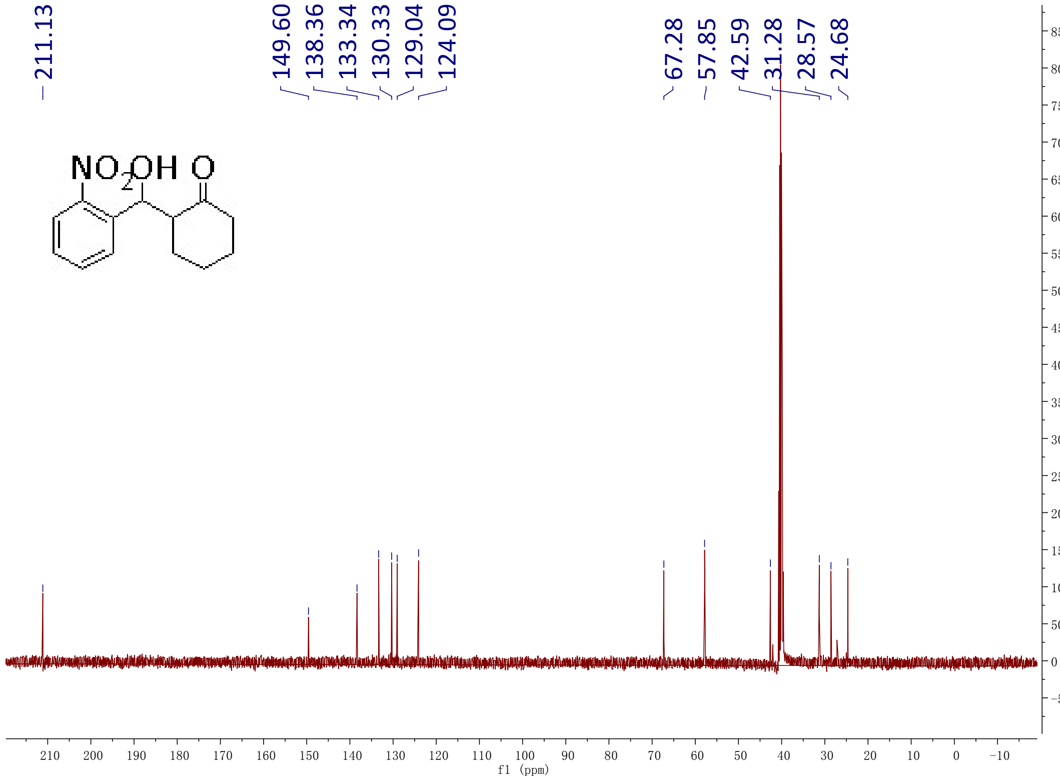

^{1}H NMR spectra of 4d

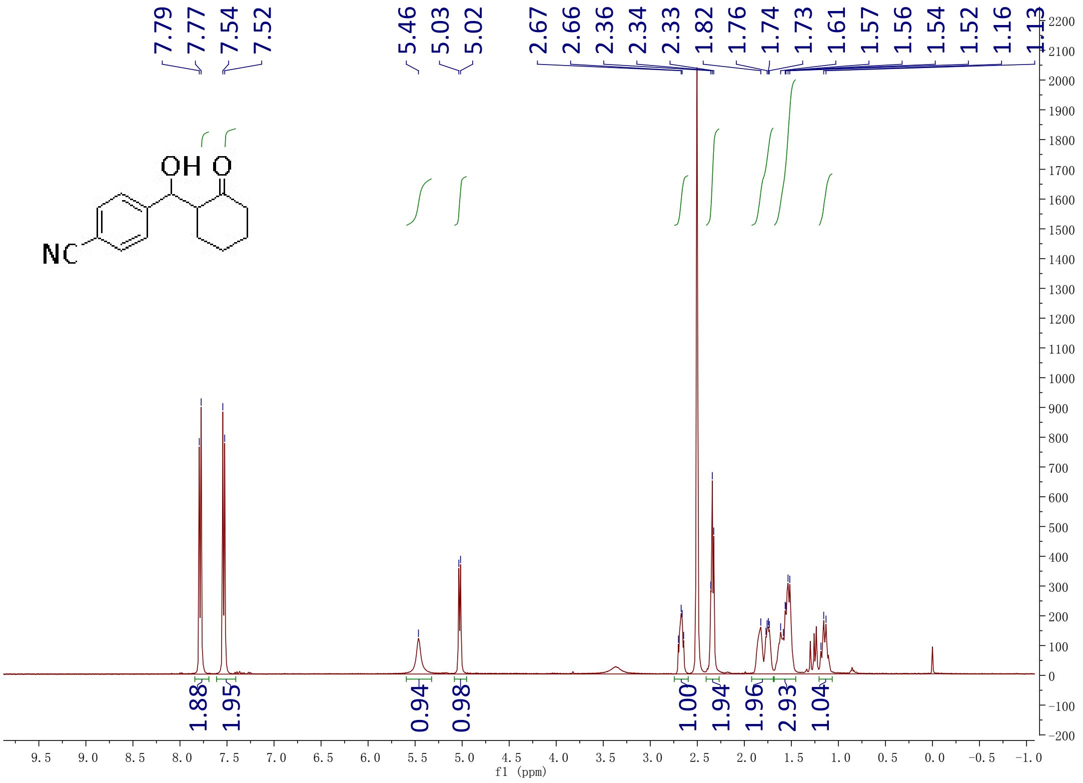

^{13}C NMR spectra of 4d

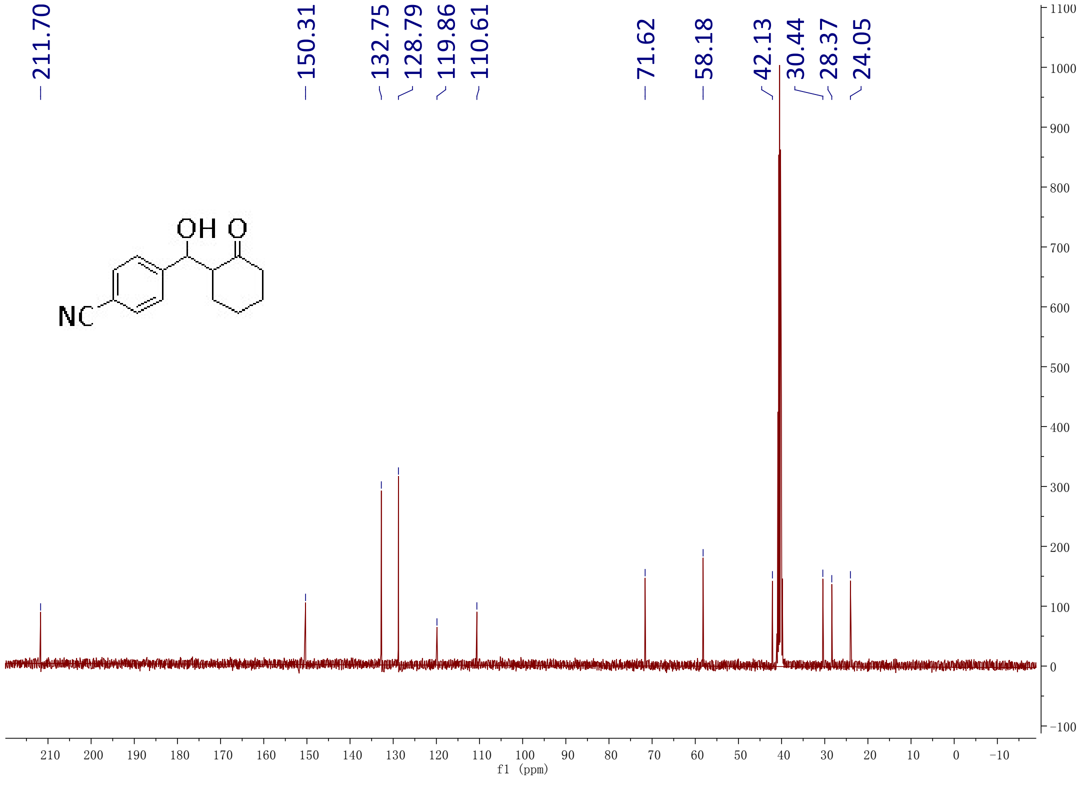

^{1}H NMR spectra of 4e

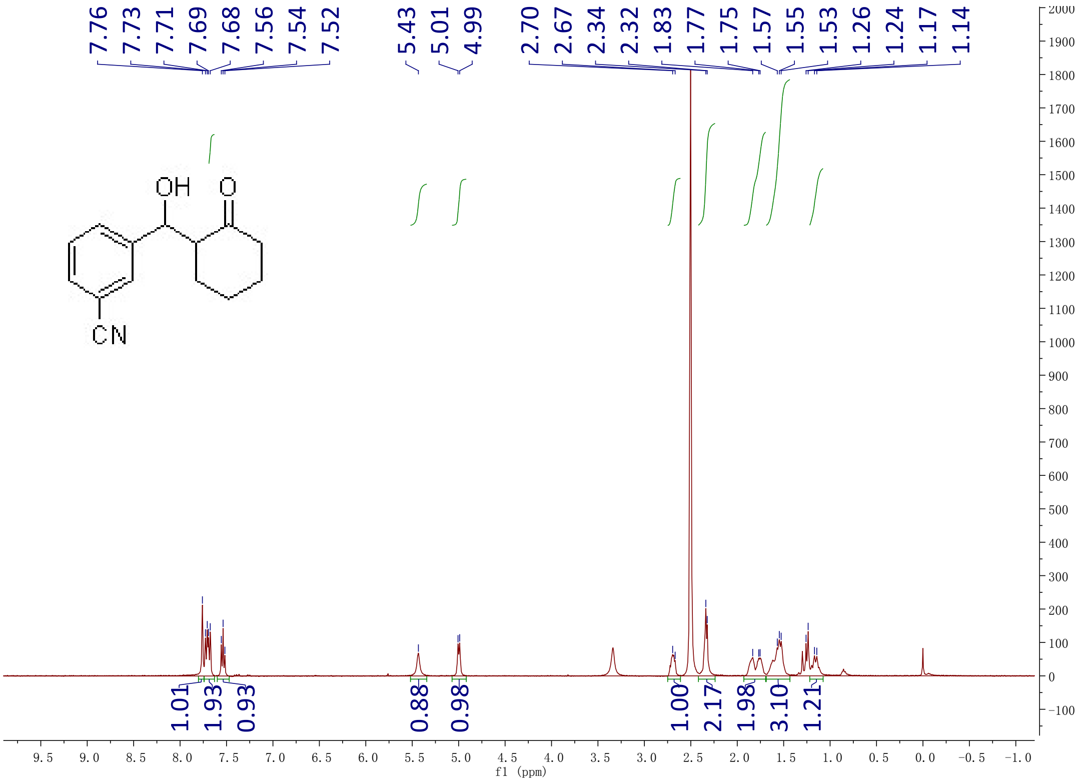

^{13}C NMR spectra of 4e

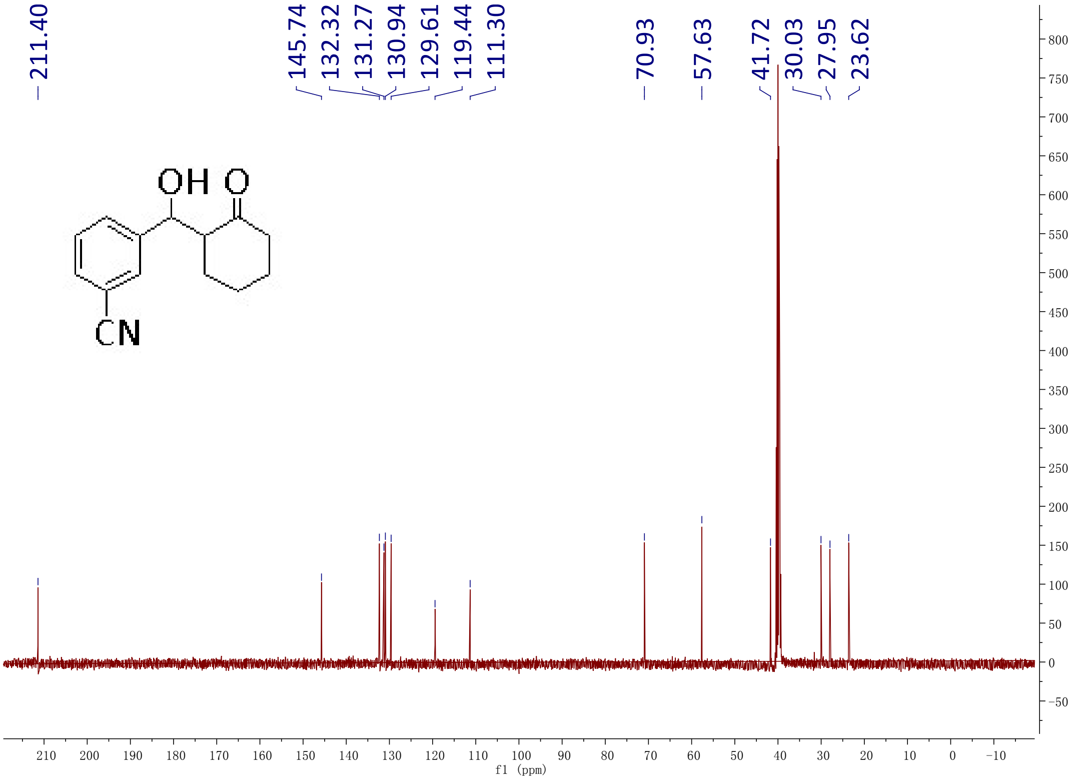

^{1}H NMR spectra of 4f

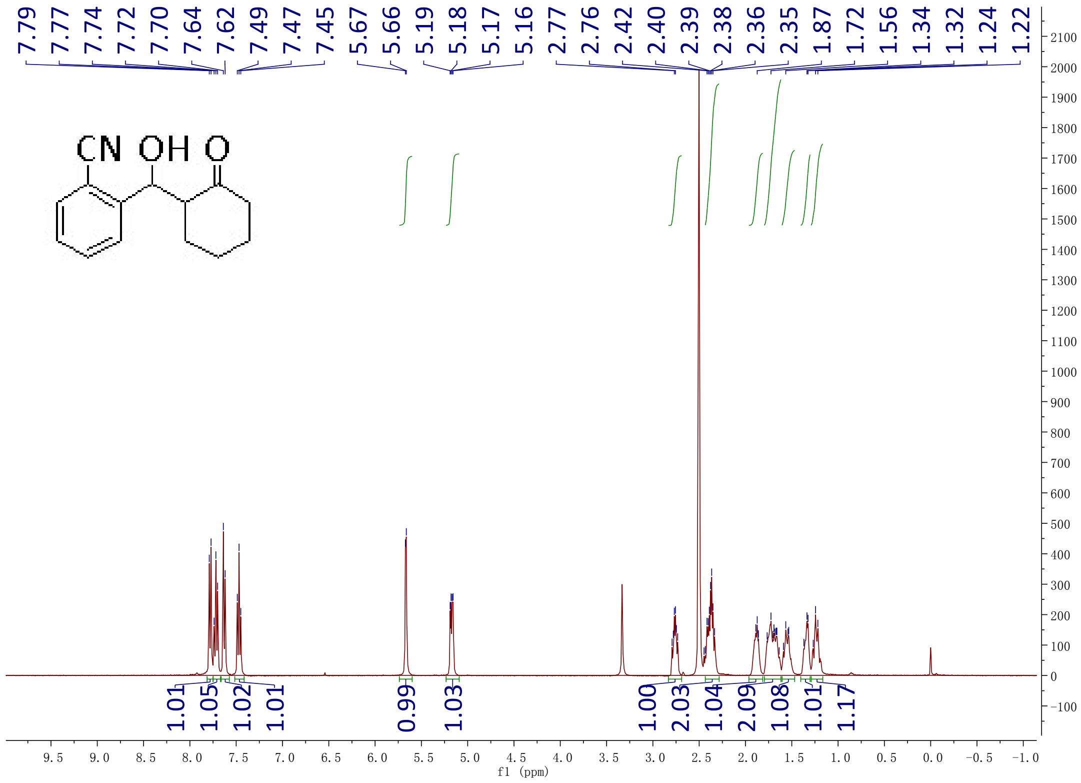

^{13}C NMR spectra of 4f

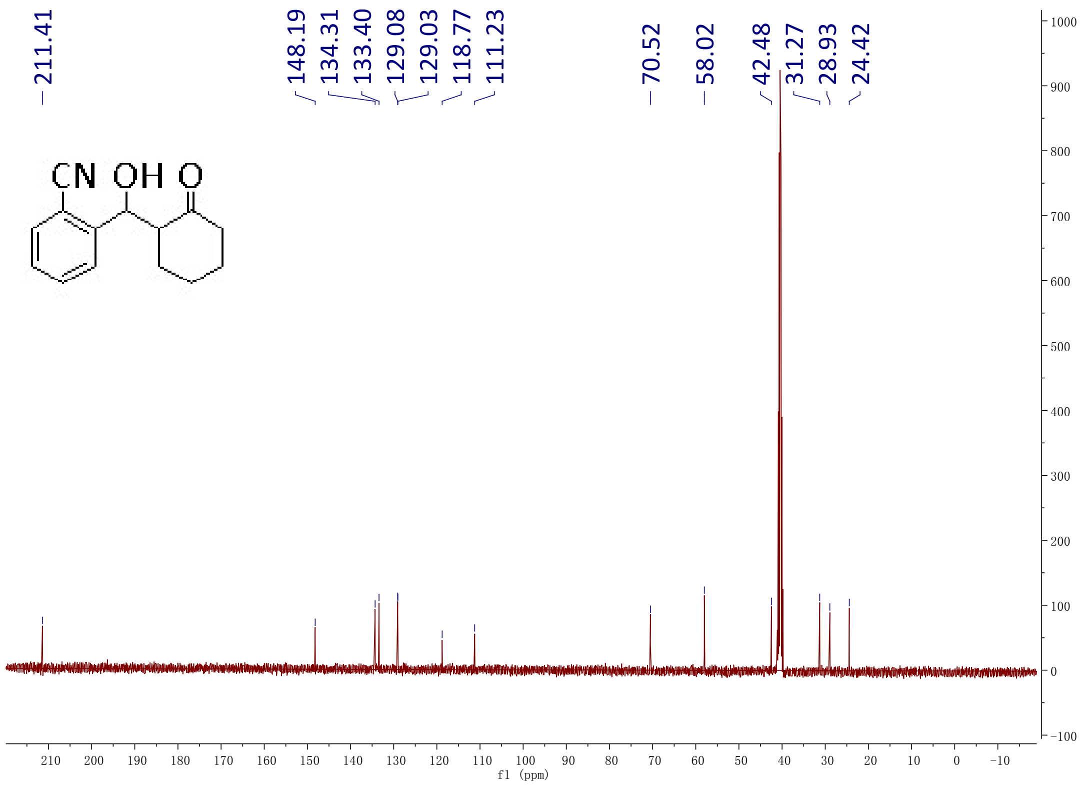

^{1}H NMR spectra of 4g

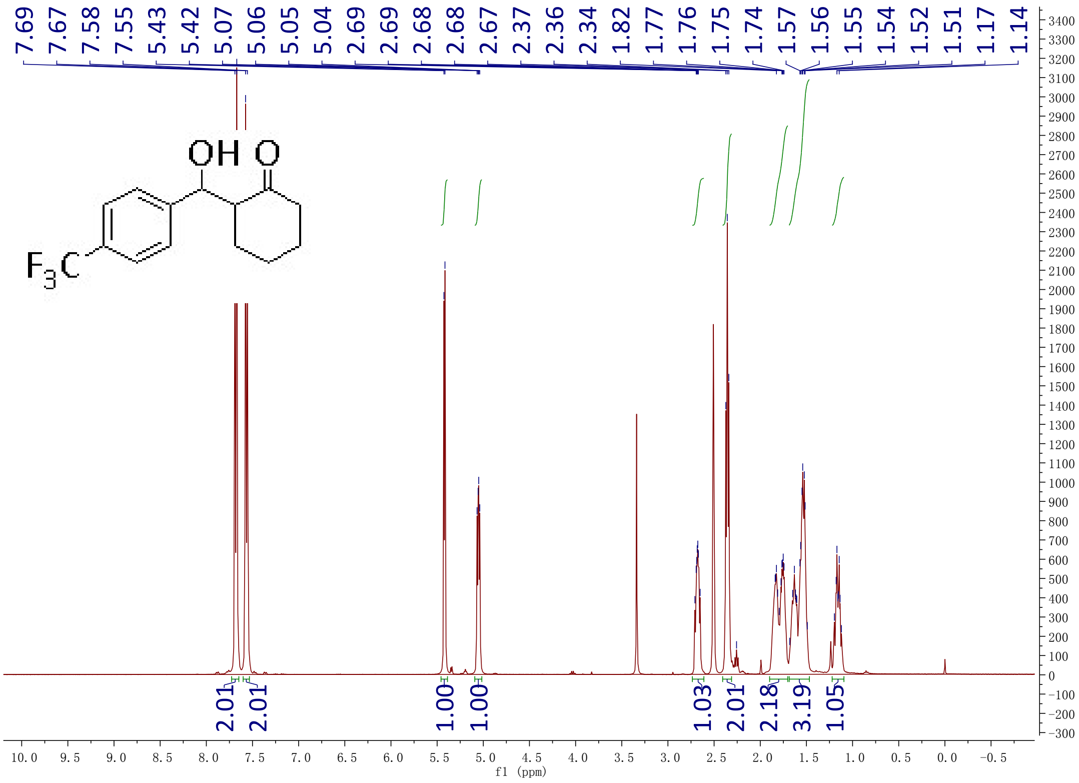

^{13}C NMR spectra of 4g

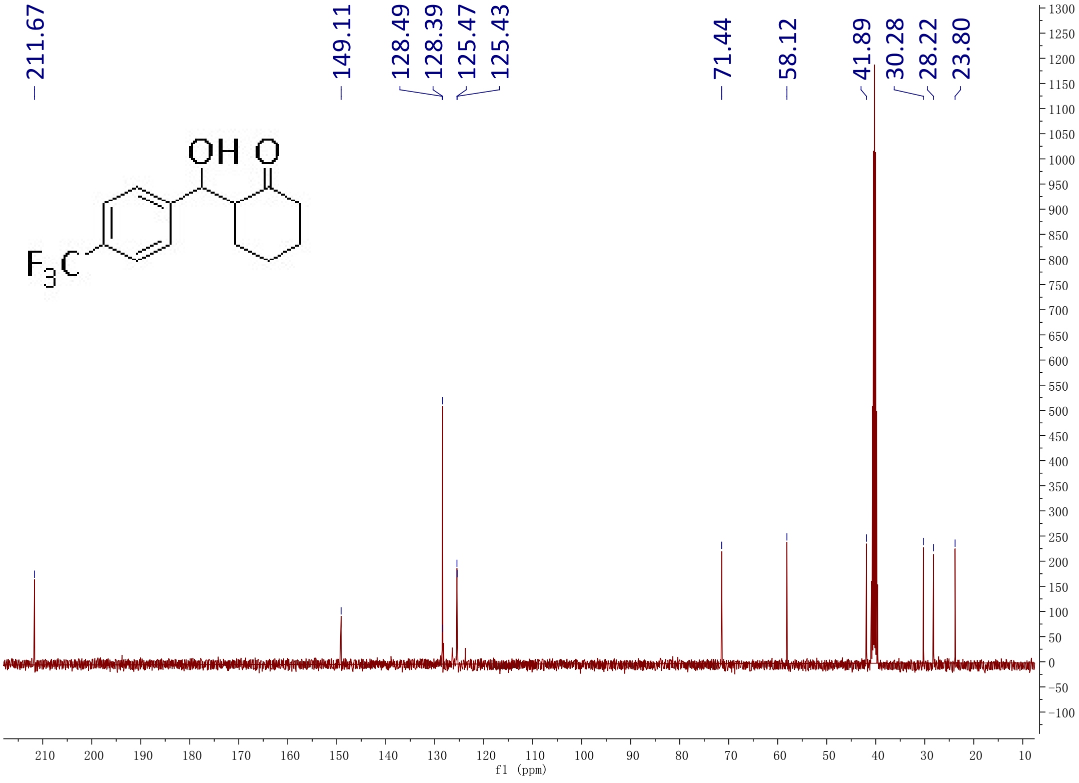

^{1}H NMR spectra of 4h

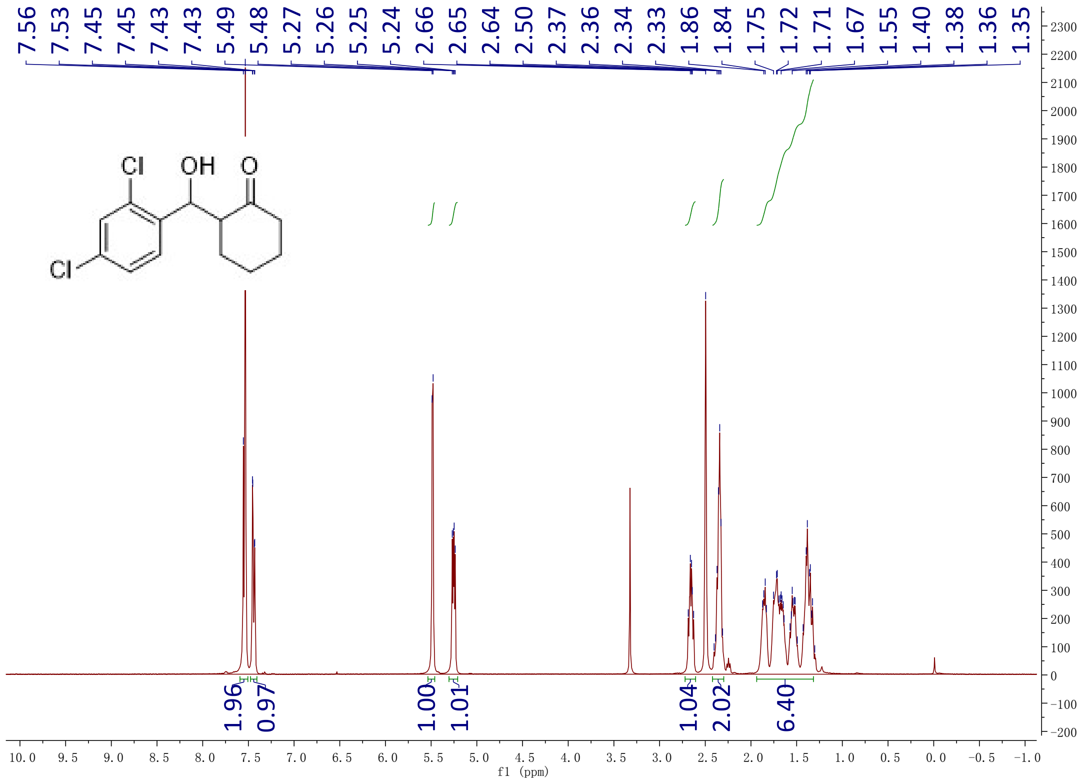

^{13}C NMR spectra of 4h

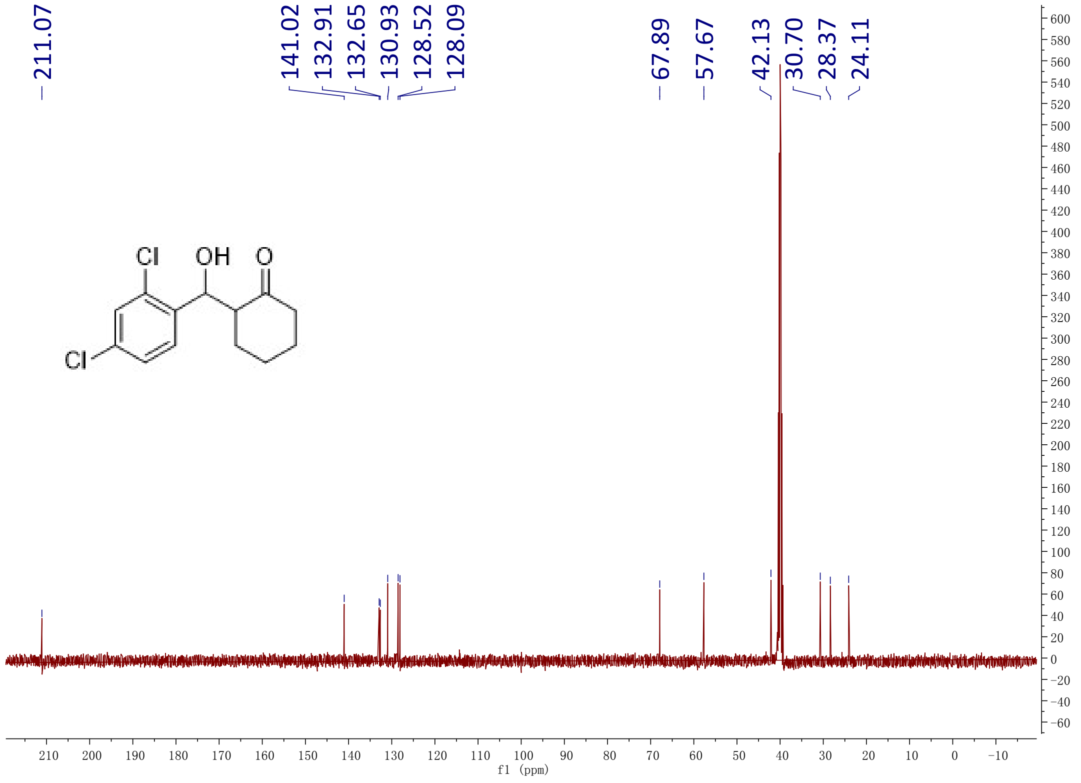

^{1}H NMR spectra of 4i

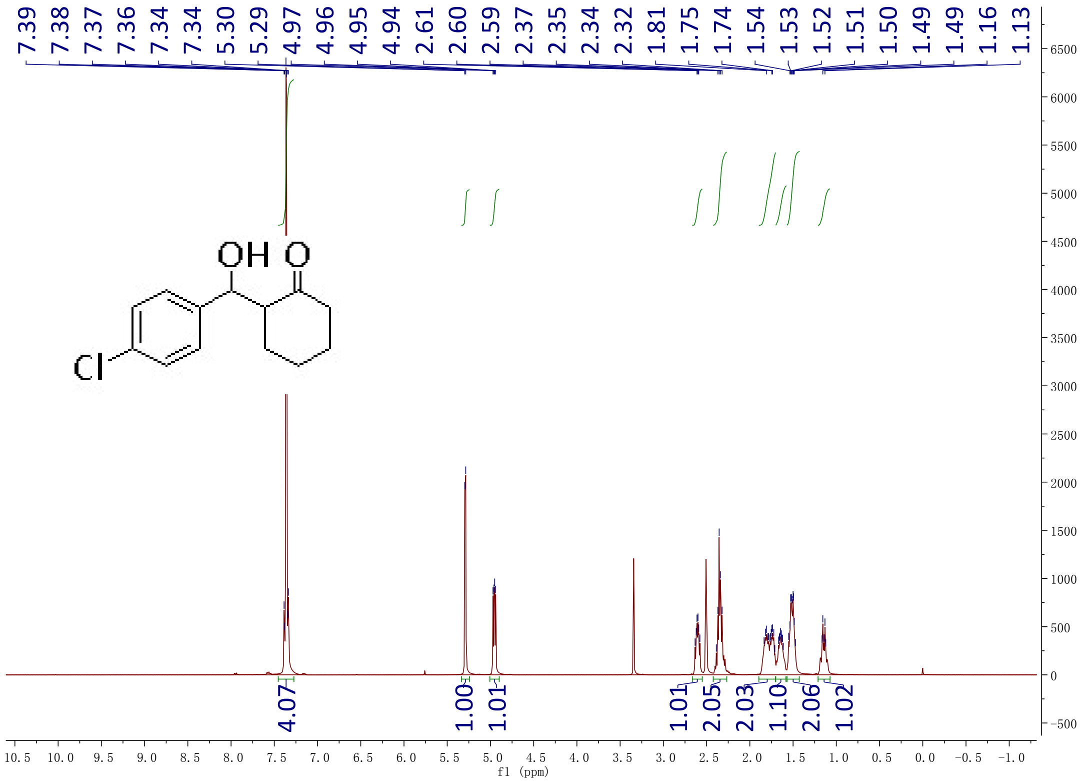

^{13}C NMR spectra of 4i

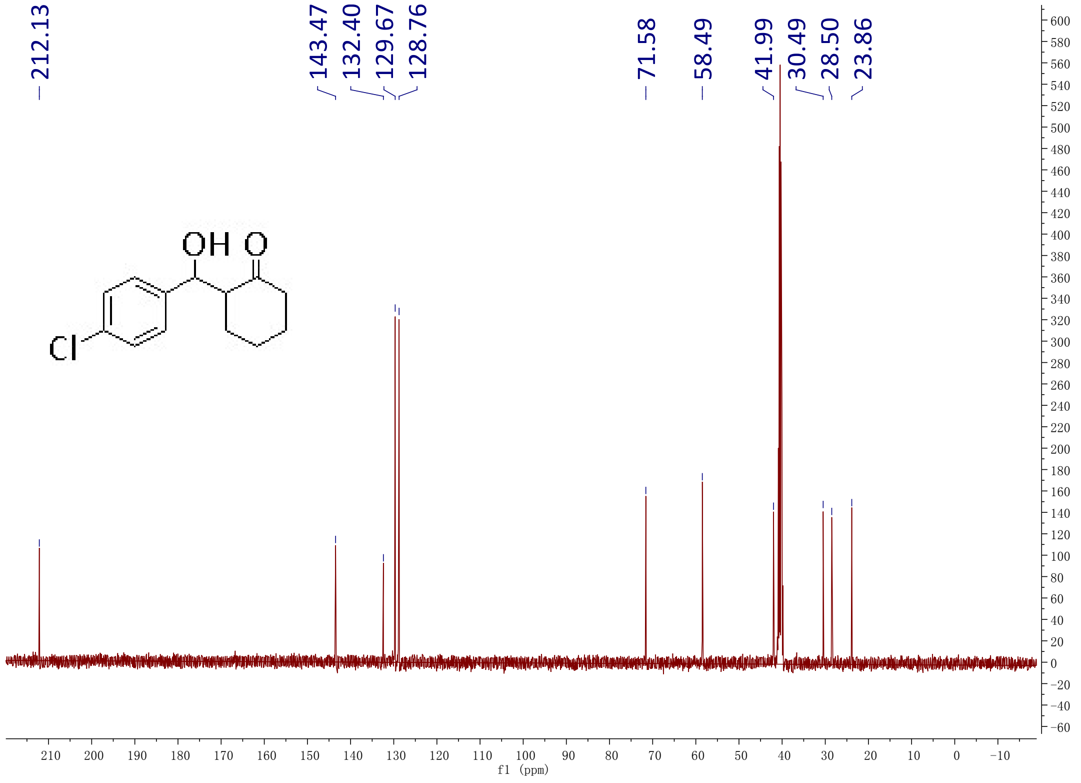

^{1}H NMR spectra of 4j

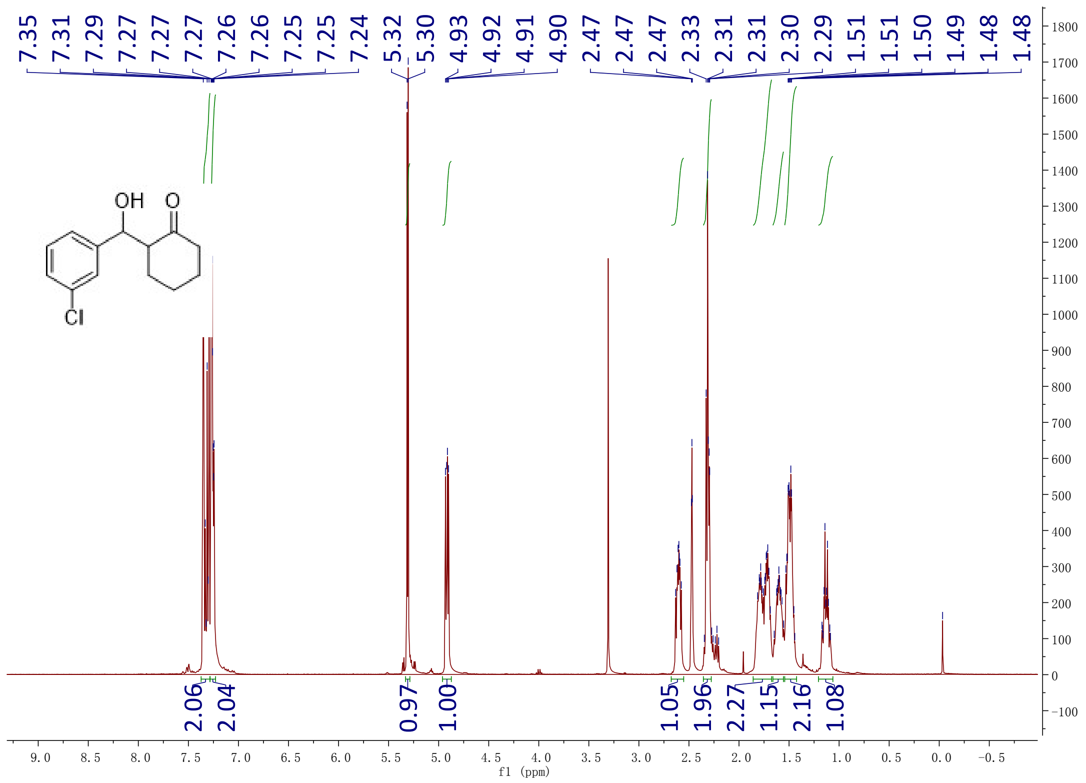

^{13}C NMR spectra of 4j

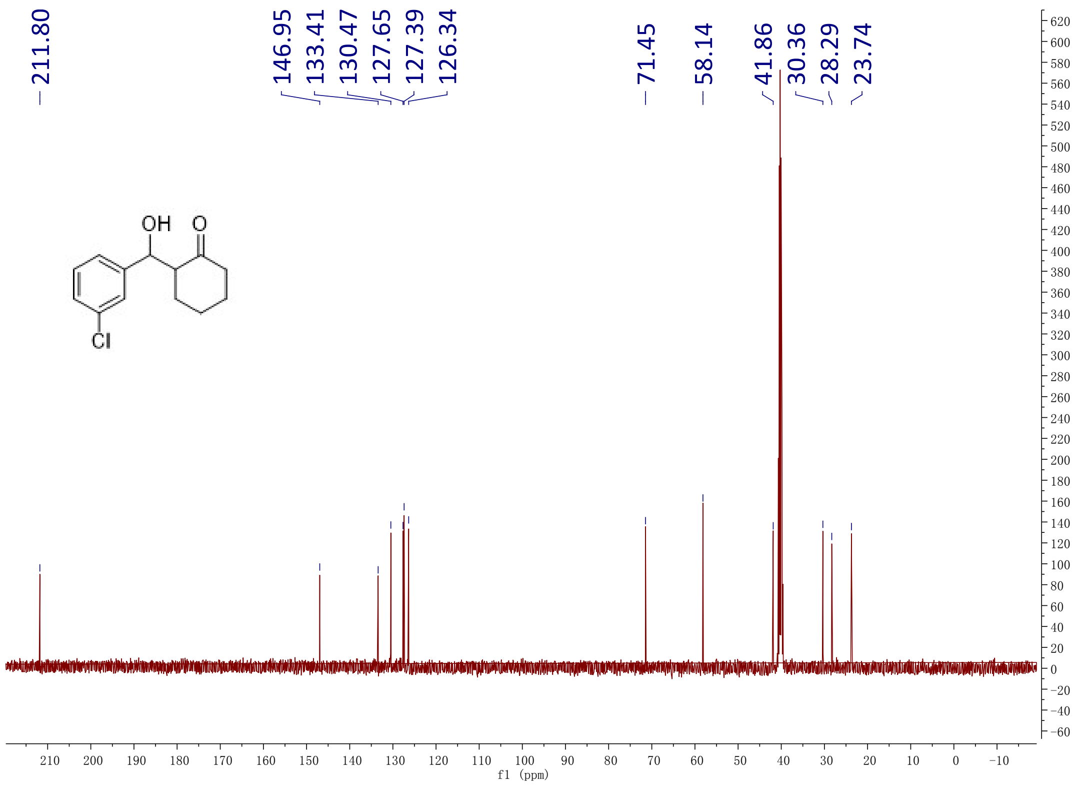

^{1}H NMR spectra of 4k

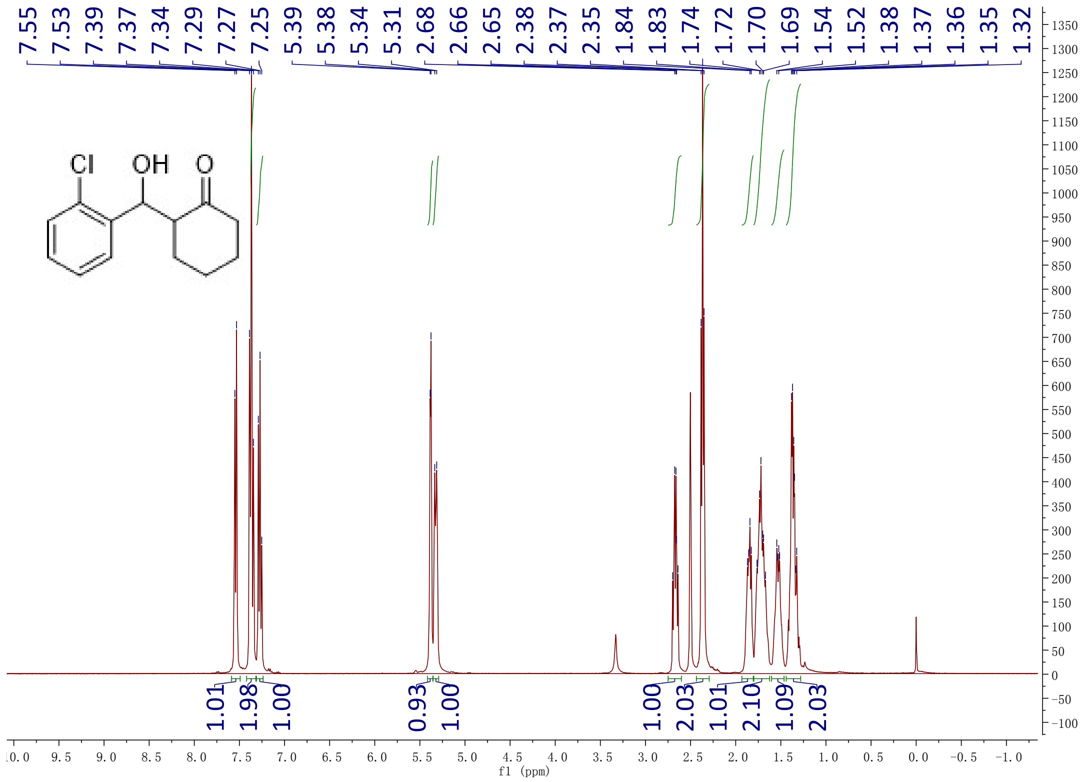

^{13}C NMR spectra of 4k

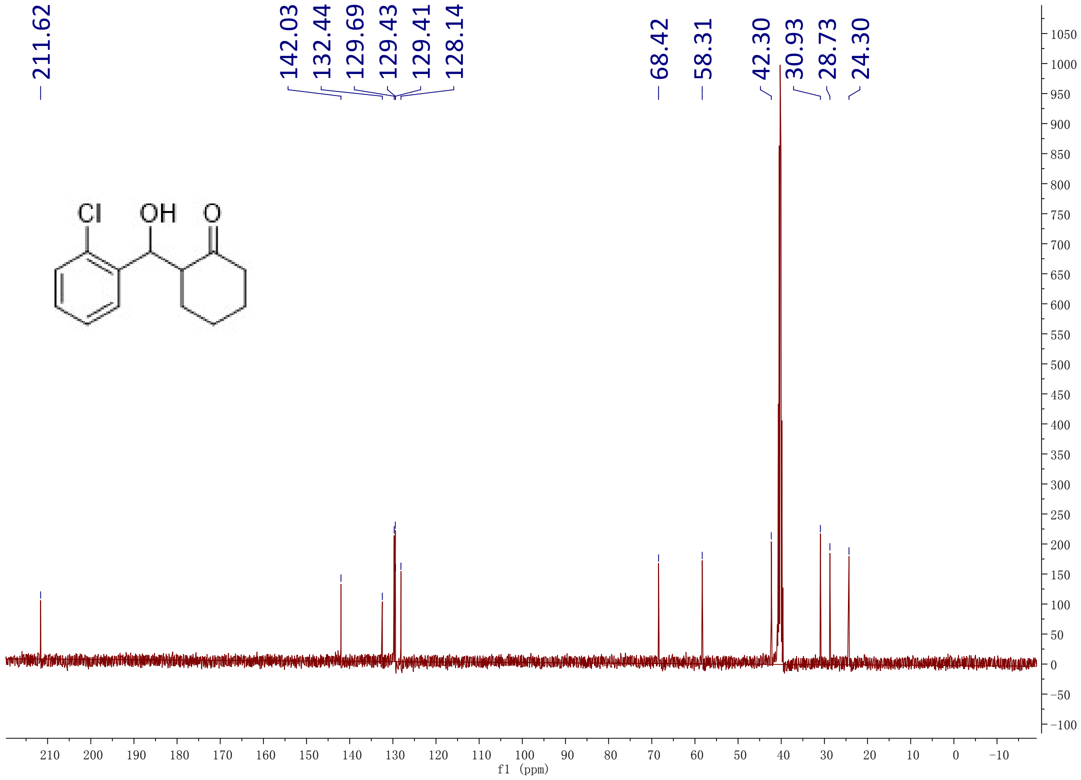

^{1}H NMR spectra of 4l

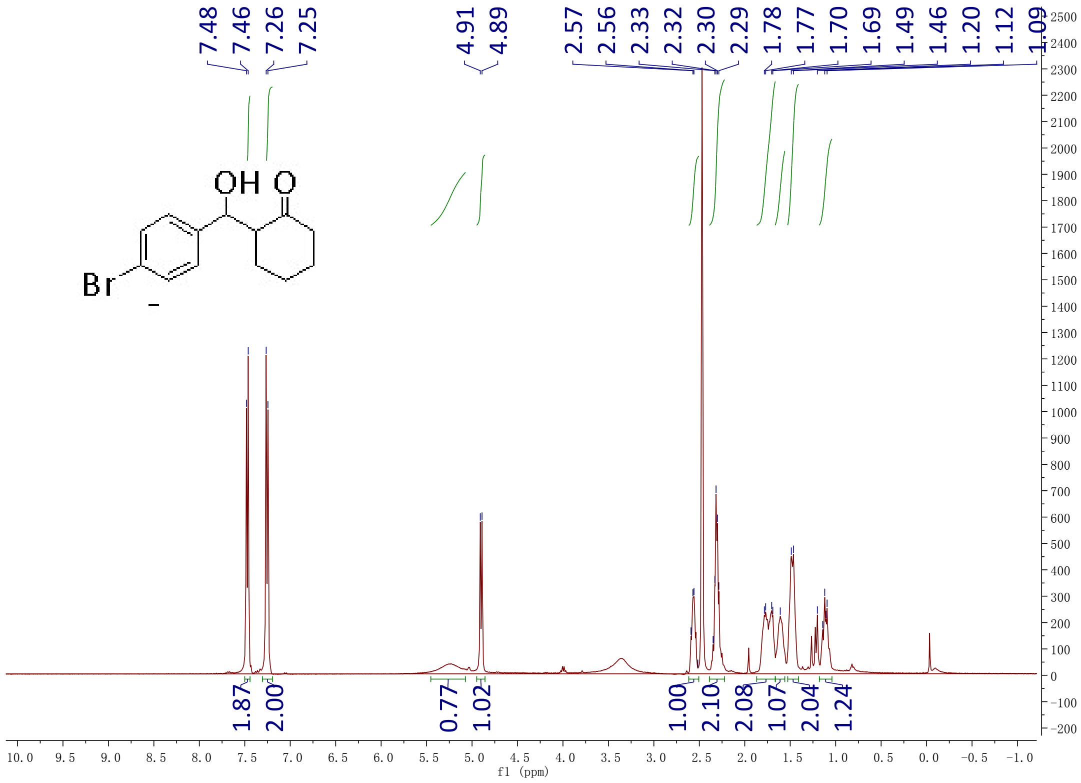

^{13}C NMR spectra of 4l

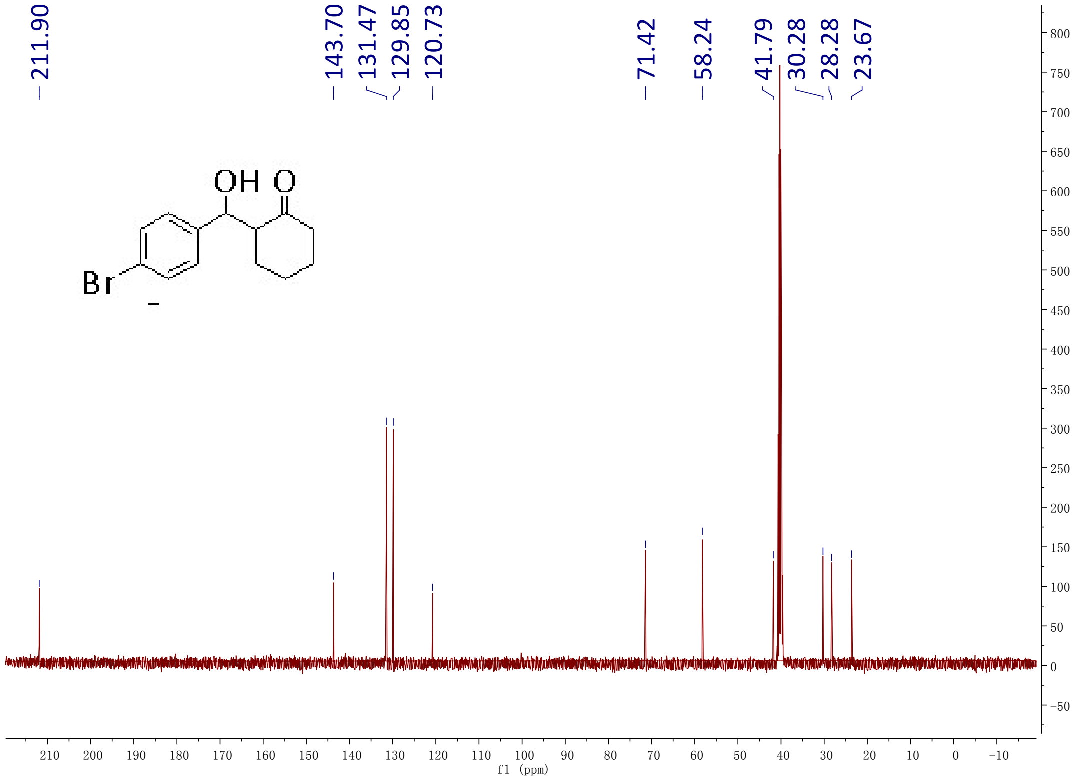

^{1}H NMR spectra of 4m

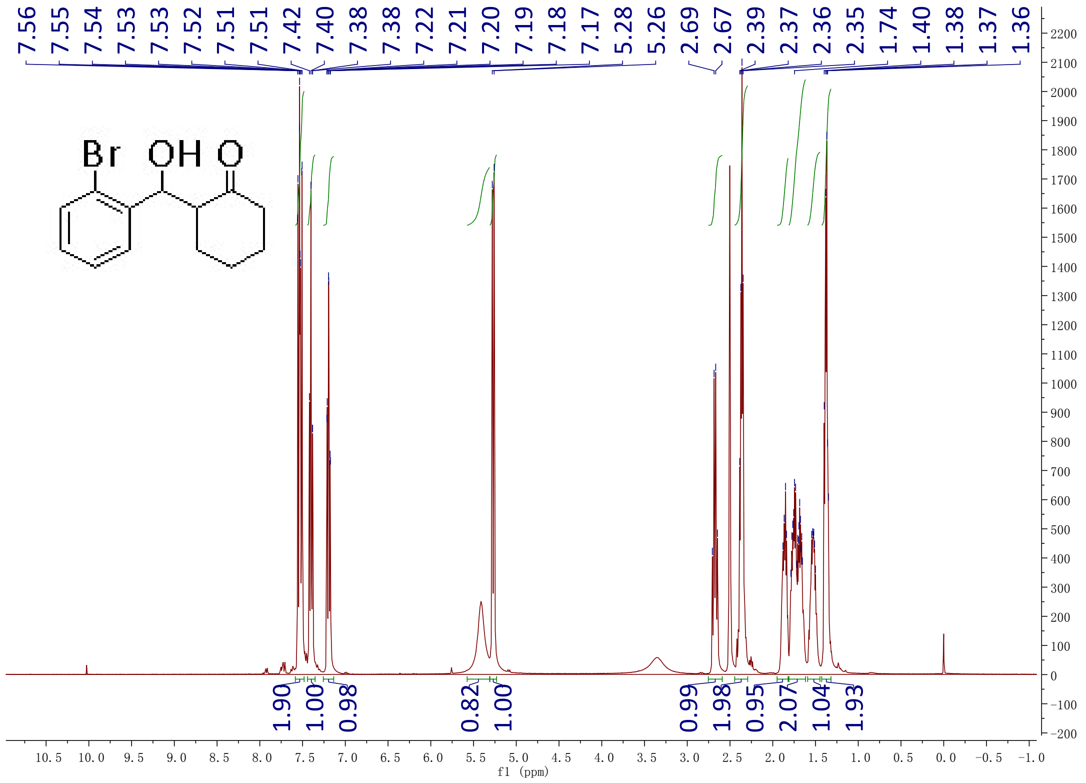

^{13}C NMR spectra of 4m

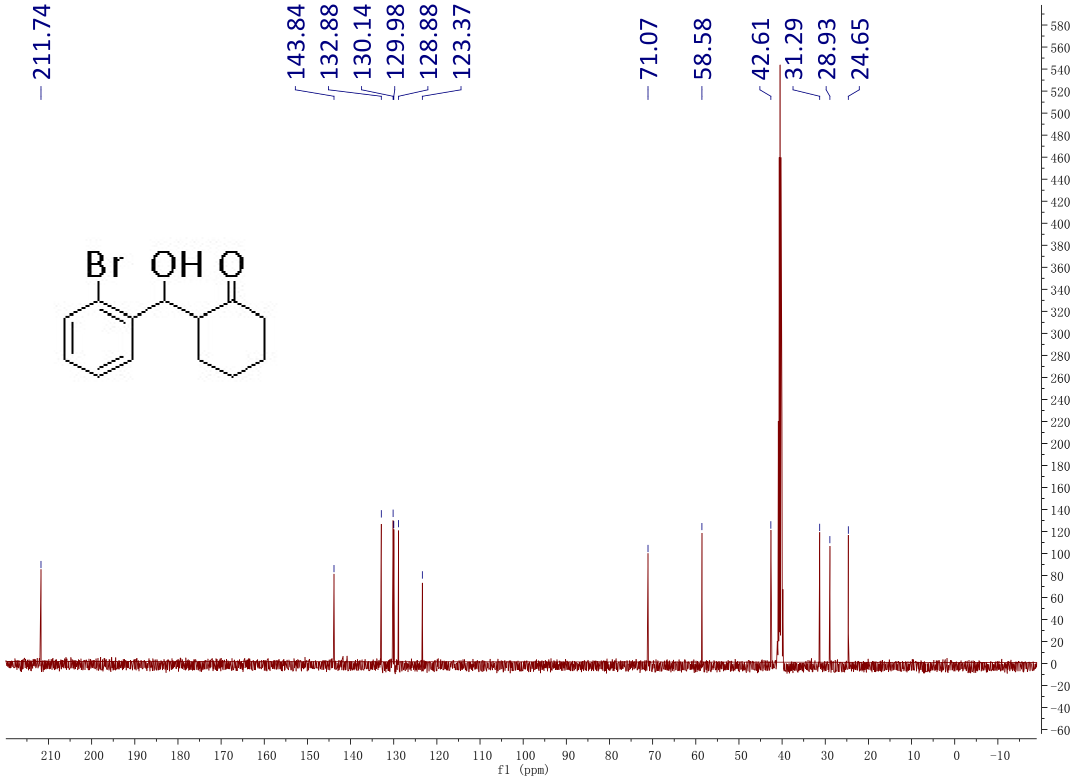

^{1}H NMR spectra of 4n

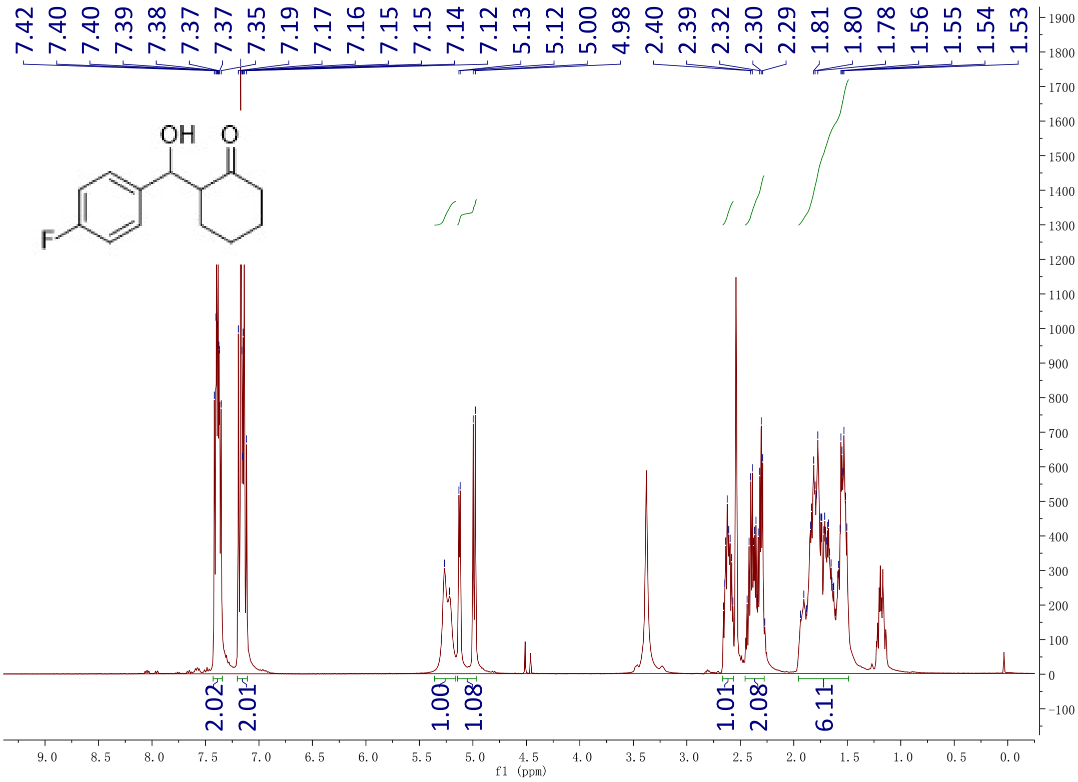

^{13}C NMR spectra of 4n

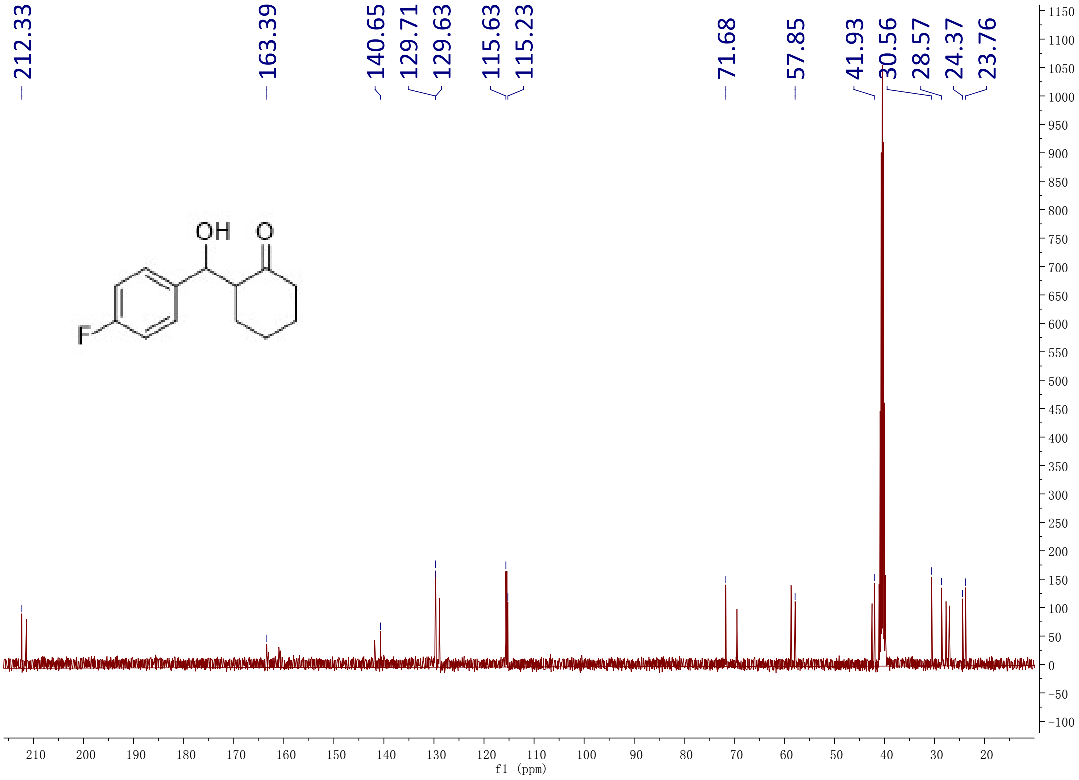

^{1}H NMR spectra of 4o

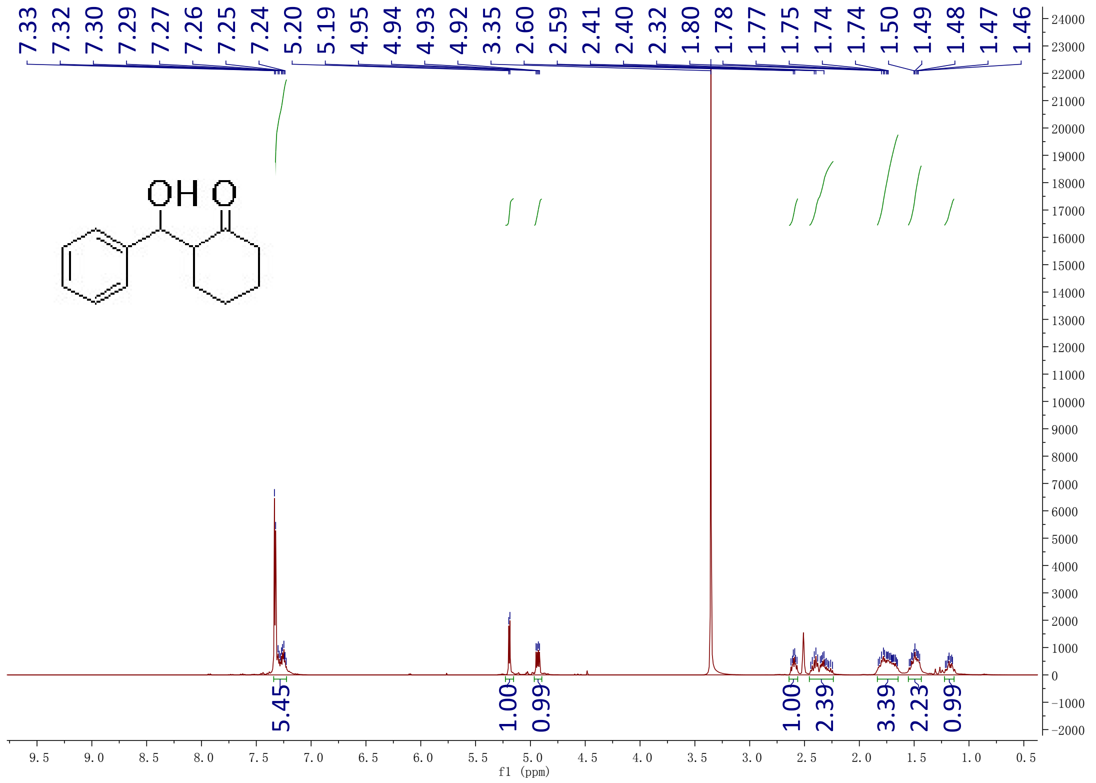

^{13}C NMR spectra of 4o

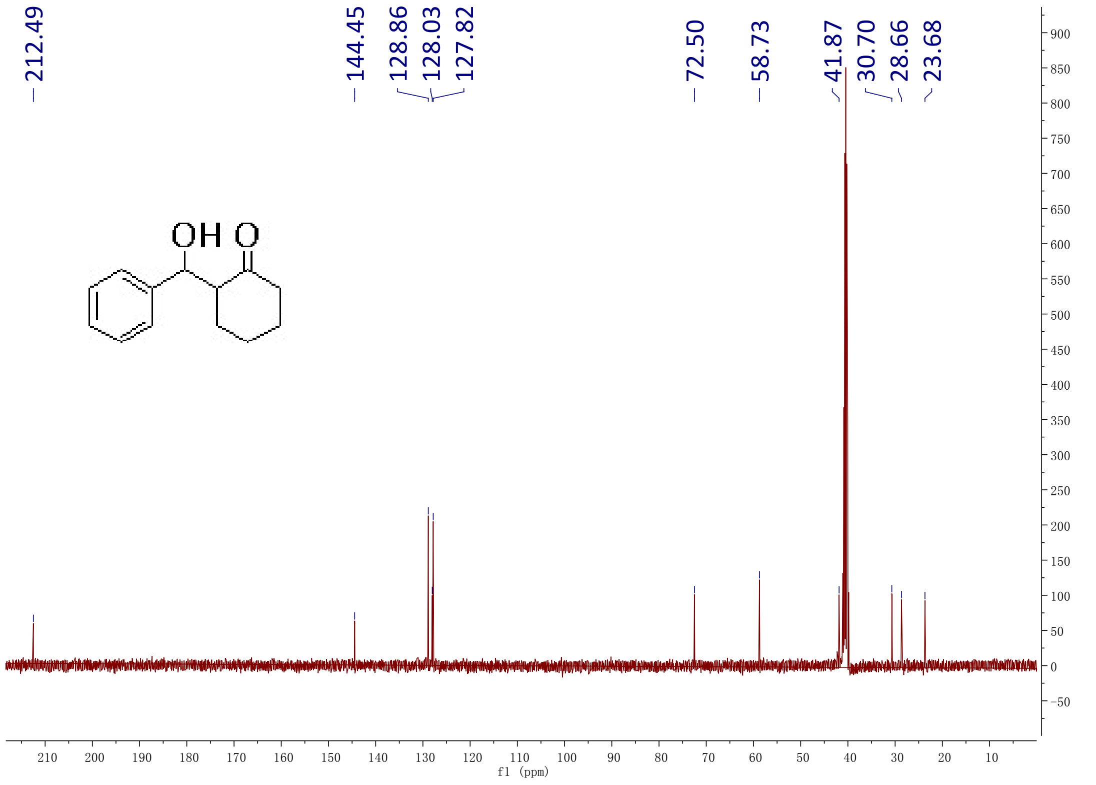

^{1}H NMR spectra of 4p

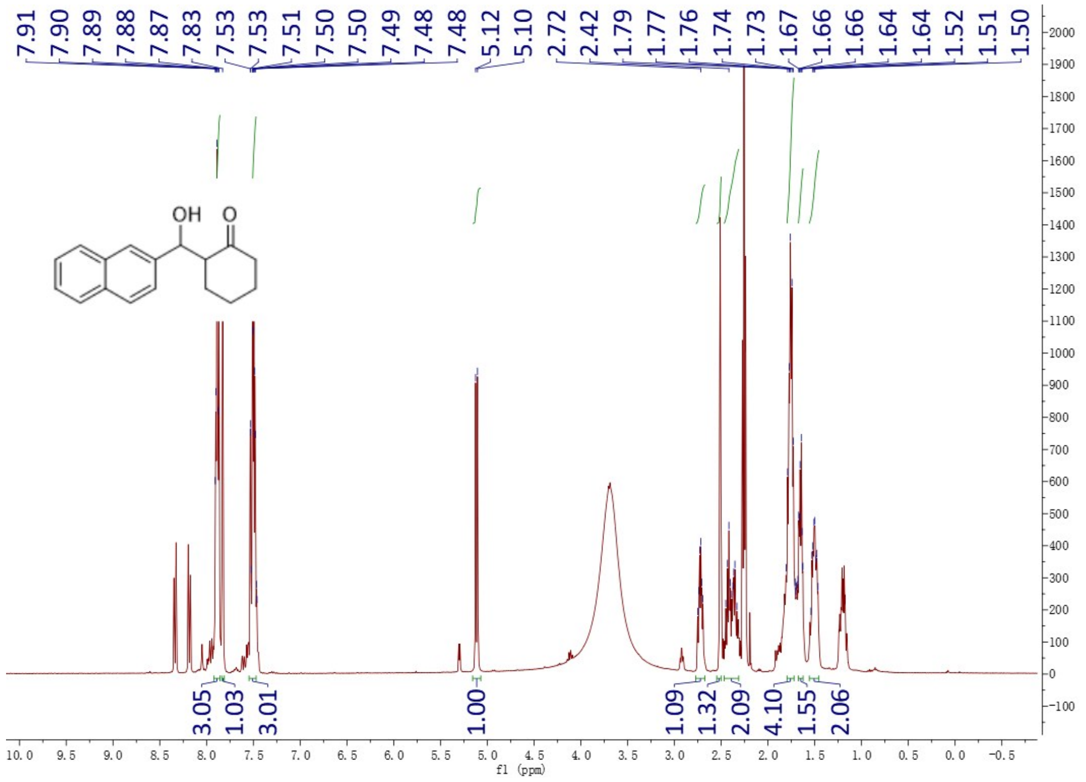

^{13}C NMR spectra of 4p

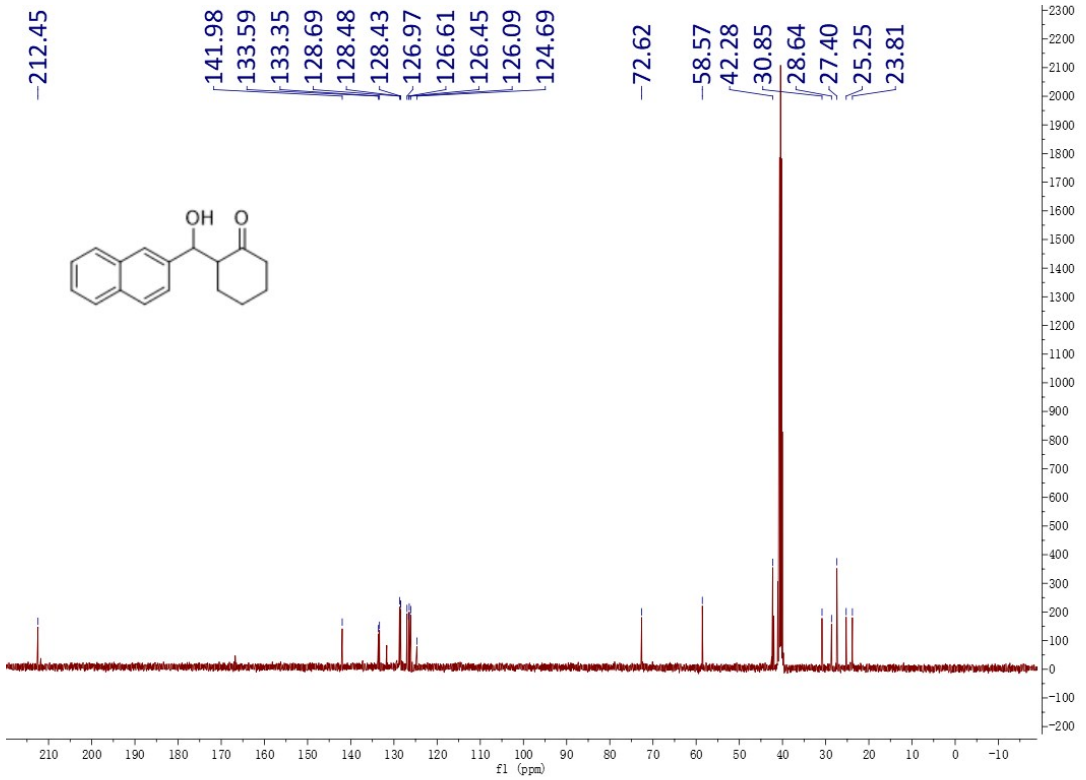

^{1}H NMR spectra of 4t

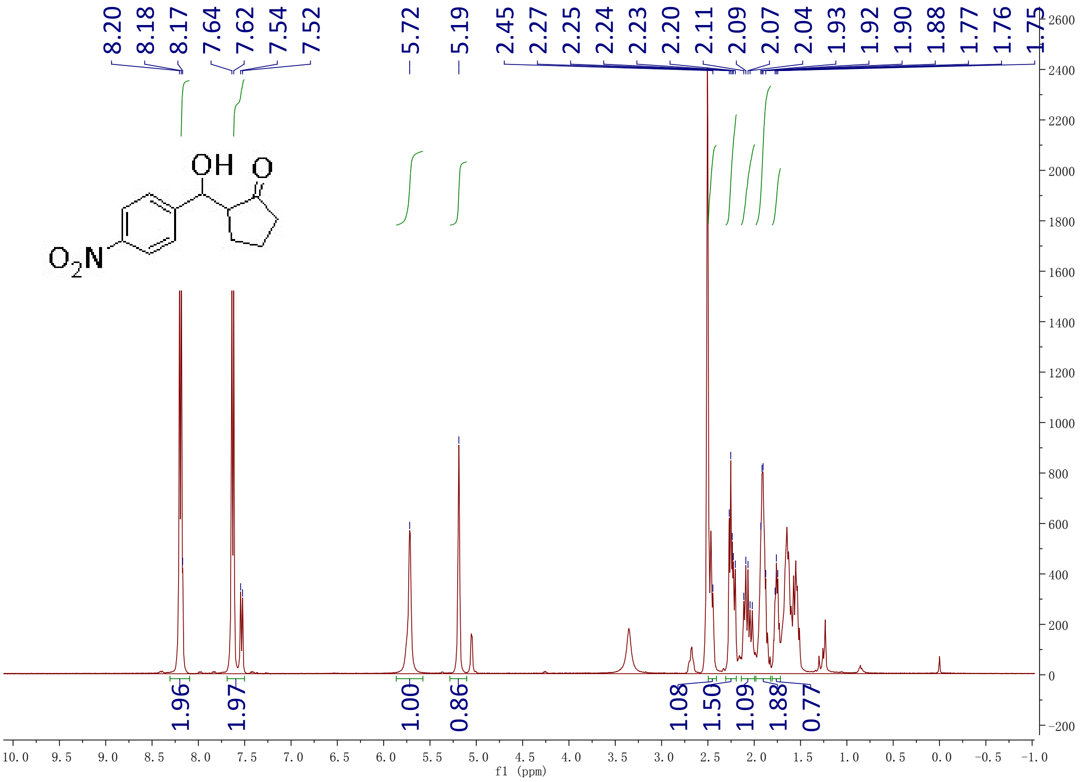

^{13}C NMR spectra of 4t

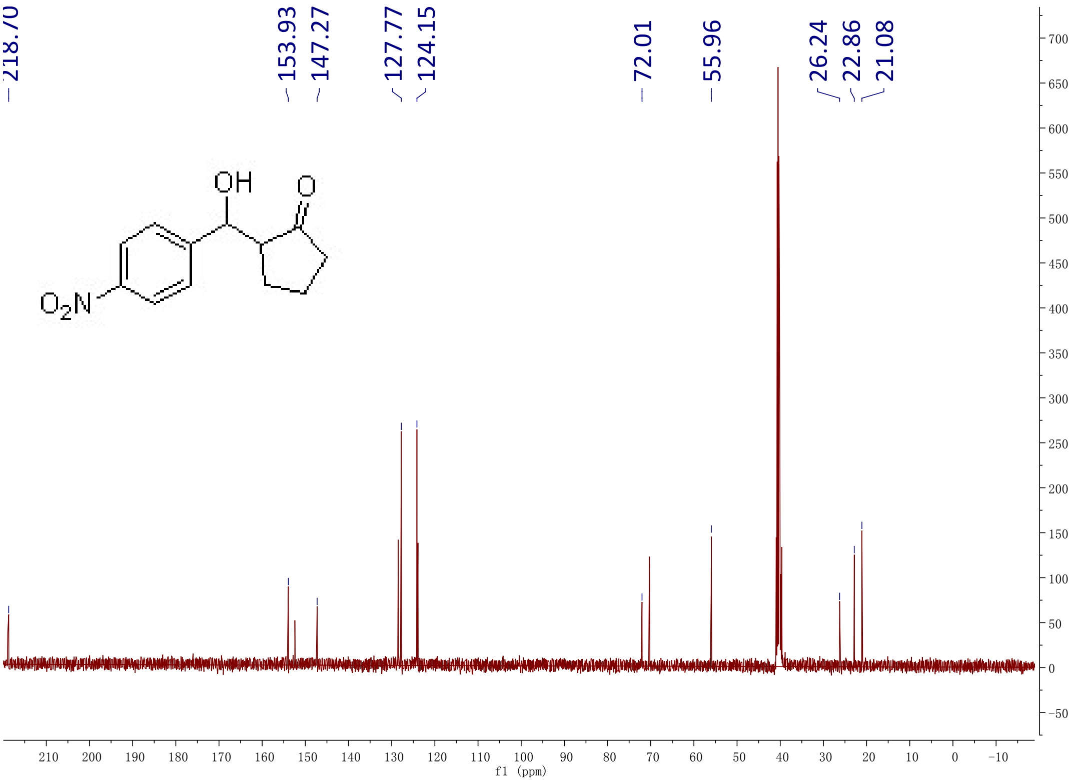

^{1}H NMR spectra of 4u

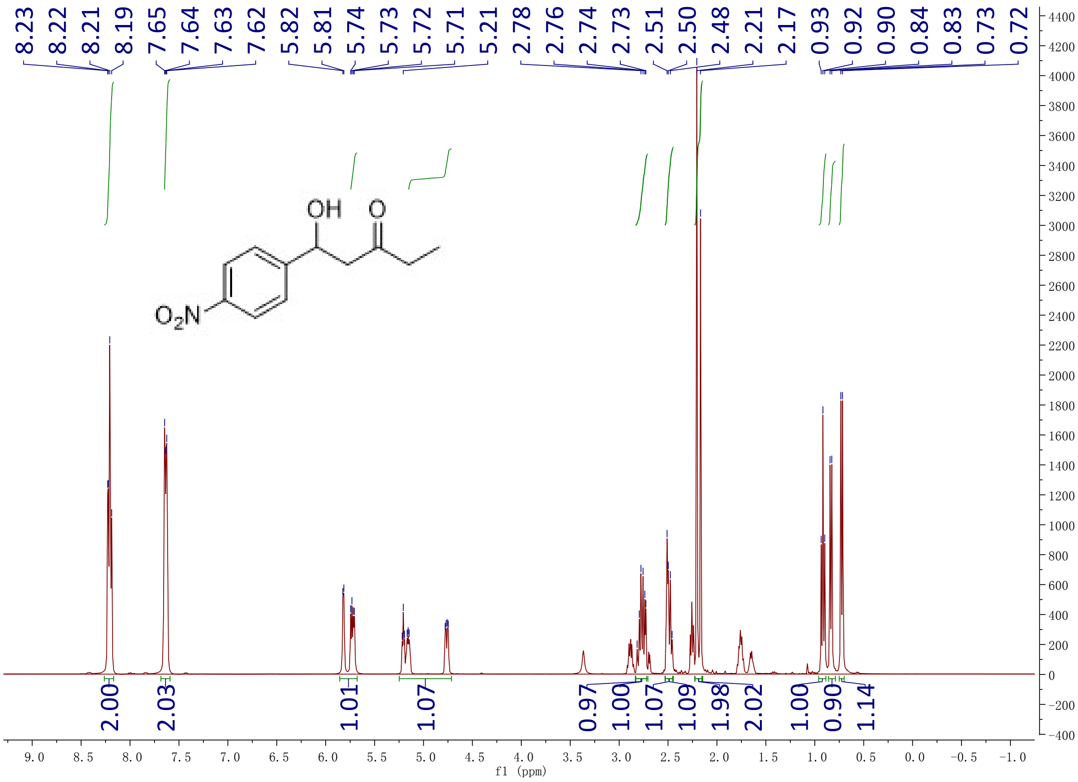

^{13}C NMR spectra of 4u

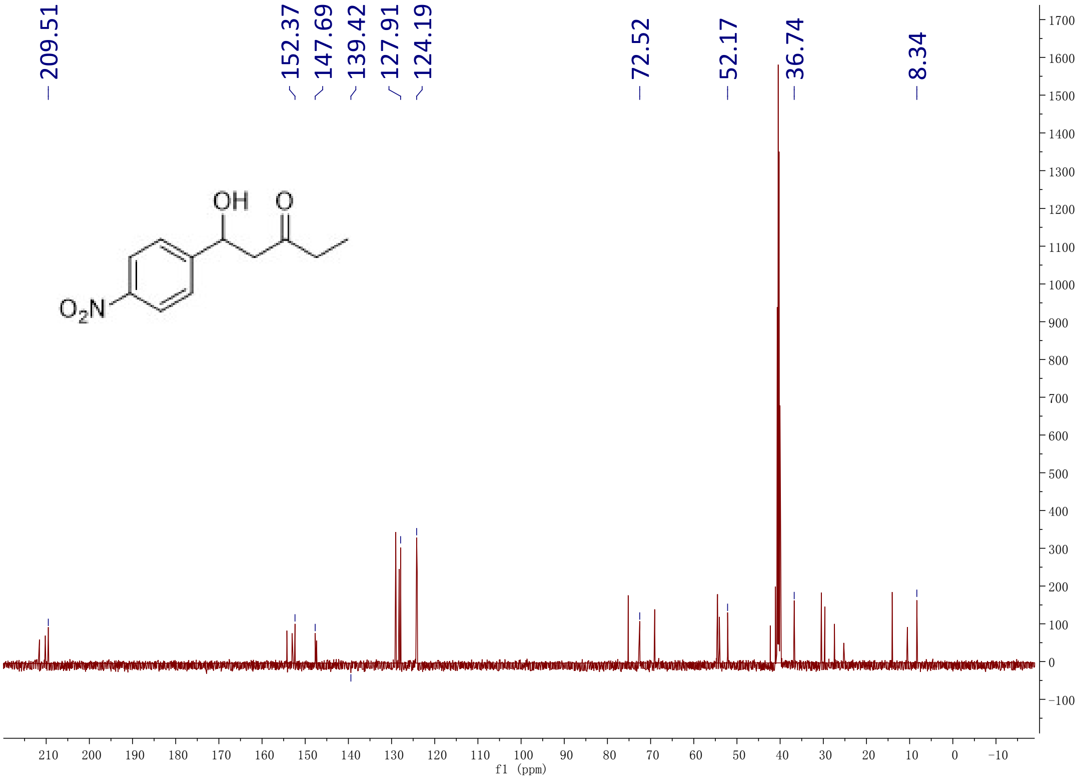

^{1}H NMR spectra of 4n

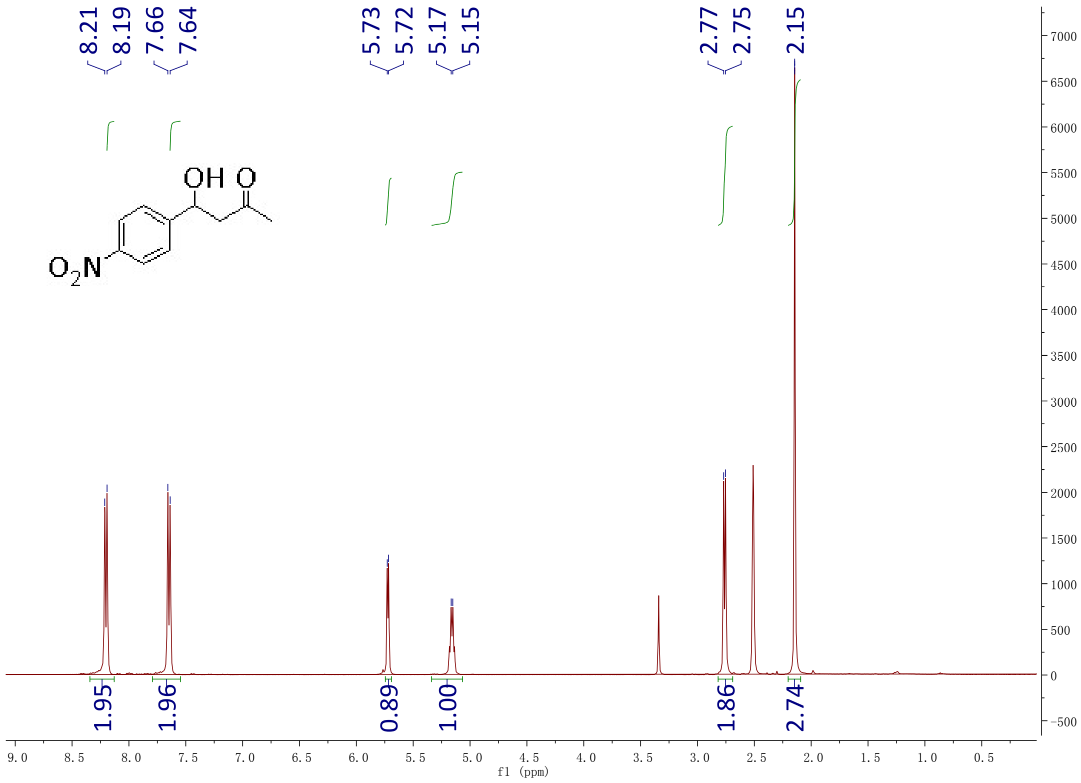

^{13}C NMR spectra of 4n

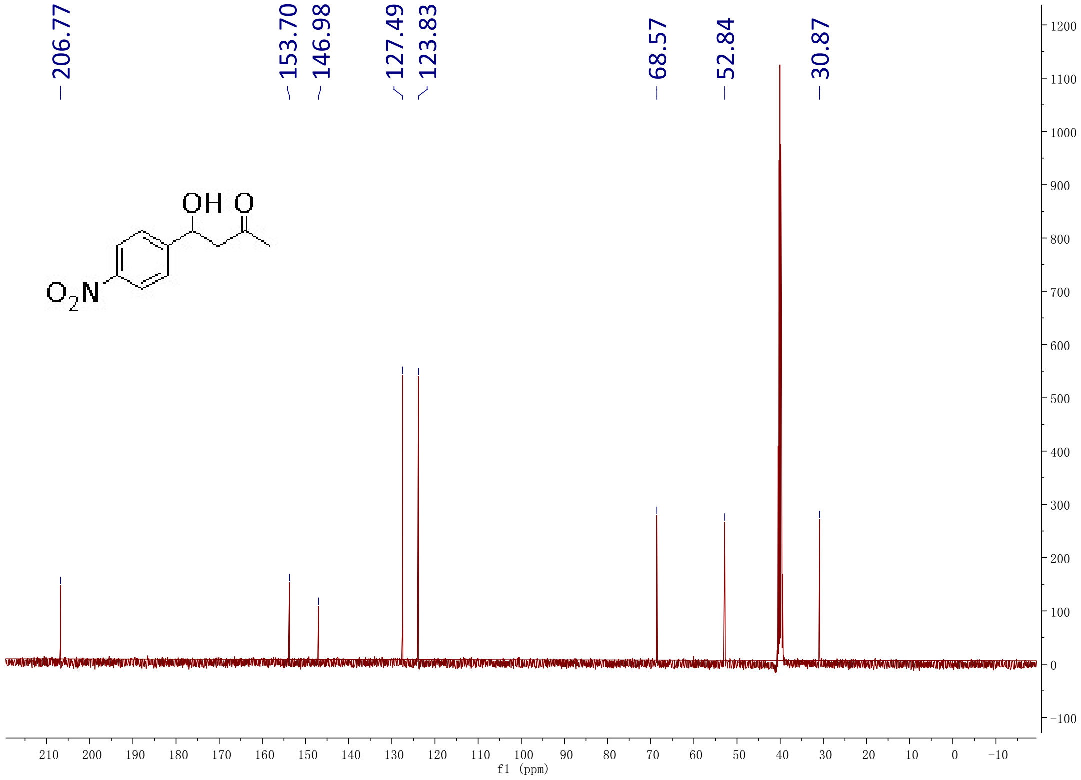

4. The HPLC of the aldol products

The HPLC of racemic 4a

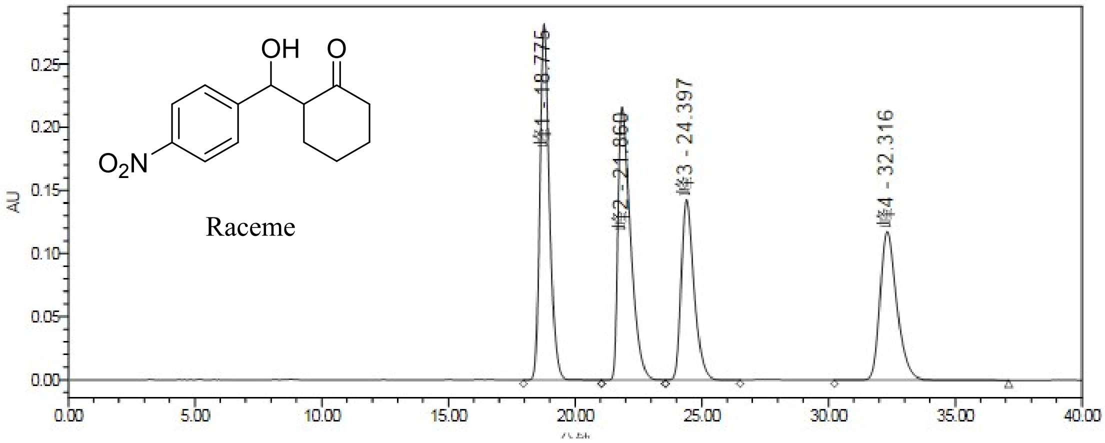

	Channel Description	Peak Name	RT (min)	Area (磺*sec)	% Area	Height (磺)
1	W2489 ChA 254nm	峰1	18.775	7391474	29.28	281501
2	W2489 ChA 254nm	峰2	21.860	7408829	29.35	215817
3	W2489 ChA 254nm	峰3	24.397	5009219	19.84	142406
4	W2489 ChA 254nm	峰4	32.316	5435113	21.53	117301

The HPLC of sampe 4a

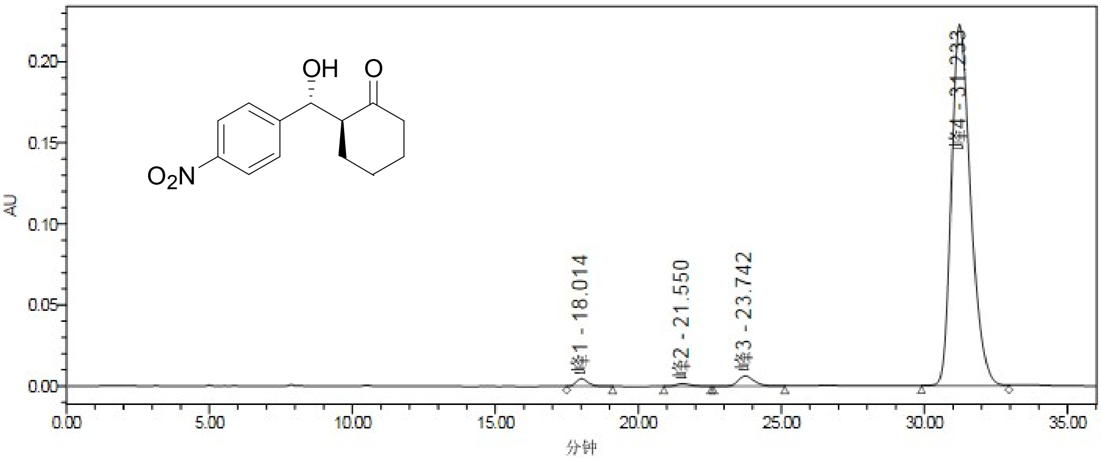

	Channel Description	Peak Name	RT (min)	Area (磺*sec)	% Area	Height (磺)
1	W2489 ChA 254nm	峰1	18.014	132753	1.23	4806
2	W2489 ChA 254nm	峰2	21.550	51135	0.47	1565
3	W2489 ChA 254nm	峰3	23.742	241274	2.23	6441
4	W2489 ChA 254nm	峰4	31.233	10393895	96.07	222682

The HPLC of racemic 4b

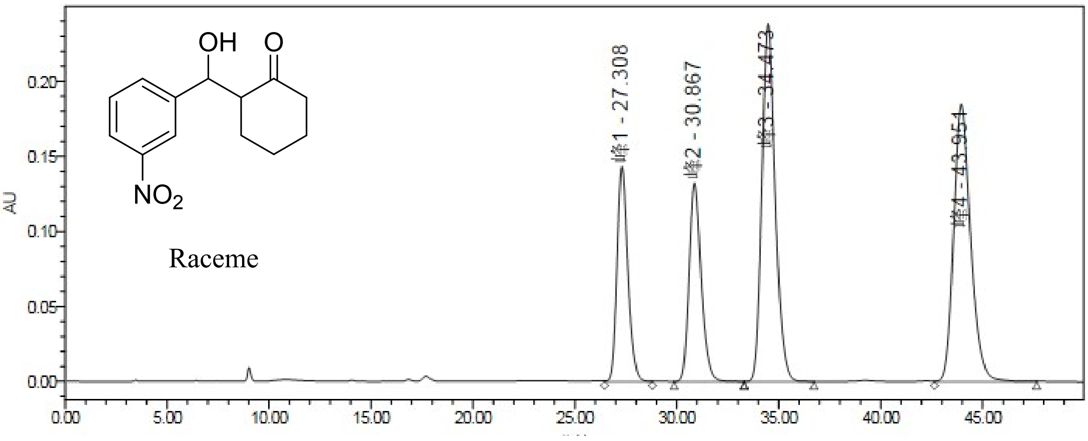

	Channel Description	Peak Name	RT (min)	Area (磺*sec)	% Area	Height (磺)
1	W2489 ChA 254nm	峰1	27.308	5236074	16.25	142904
2	W2489 ChA 254nm	峰2	30.867	5477841	17.00	132293
3	W2489 ChA 254nm	峰3	34.473	10813600	33.56	238012
4	W2489 ChA 254nm	峰4	43.951	10694507	33.19	184662

The HPLC of sample 4b

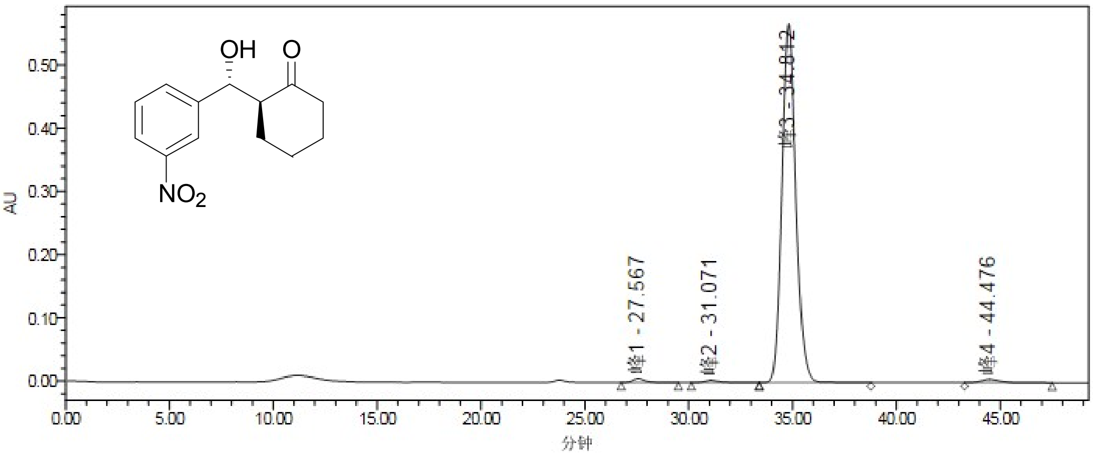

	Channel Description	Peak Name	RT (min)	Area (磺*sec)	% Area	Height (磺)
1	W2489 ChA 254nm	峰1	27.567	231129	0.86	6201
2	W2489 ChA 254nm	峰2	31.071	156742	0.58	3512
3	W2489 ChA 254nm	峰3	34.812	26275853	97.39	566875
4	W2489 ChA 254nm	峰4	44.476	316885	1.17	5139

The HPLC of racemic 4c

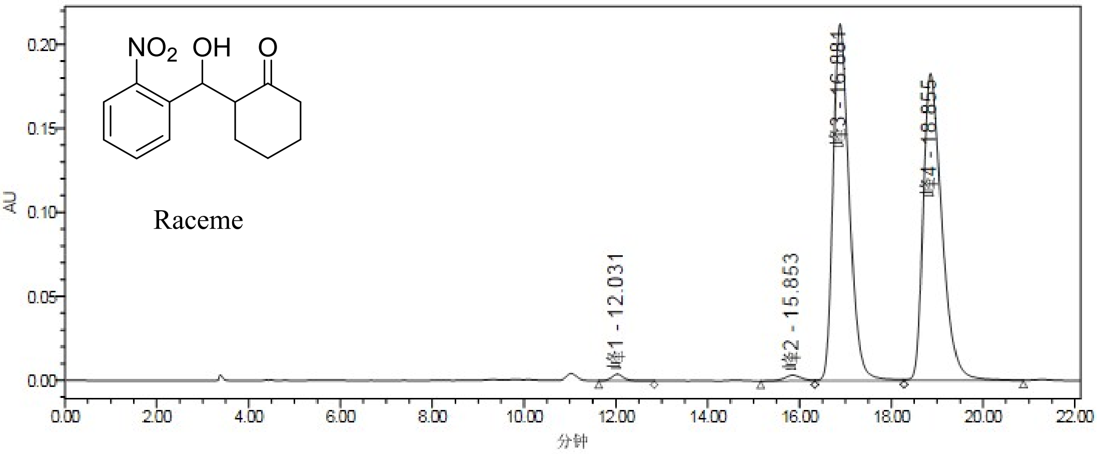

	Channel Description	Peak Name	RT (min)	Area (磺*sec)	% Area	Height (磺)
1	W2489 ChA 254nm	峰1	12.031	68692	0.65	3883
2	W2489 ChA 254nm	峰2	15.853	80024	0.76	3269
3	W2489 ChA 254nm	峰3	16.881	5181592	49.38	212186
4	W2489 ChA 254nm	峰4	18.855	5162779	49.20	182590

The HPLC of sample 4c

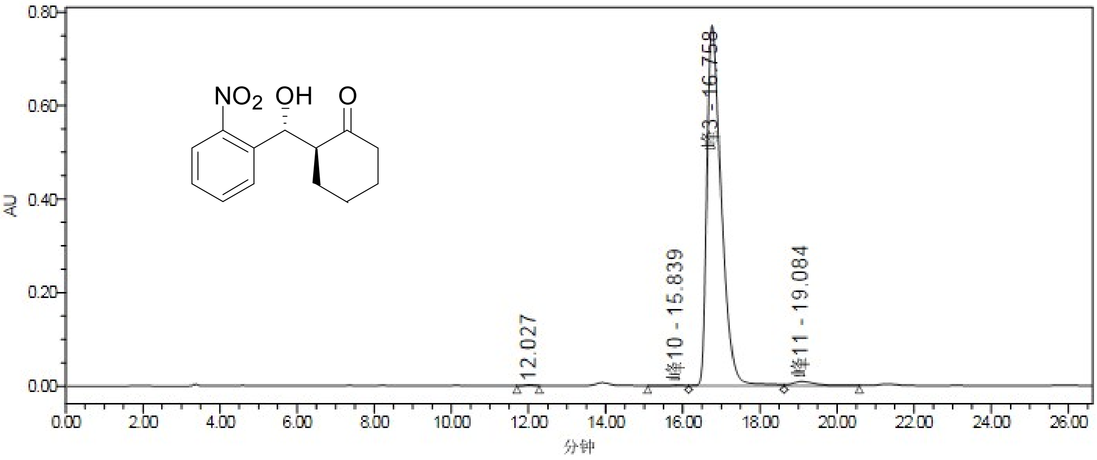

	Channel Description	Peak Name	RT (min)	Area (磺*sec)	% Area	Height (磺)
6	W2489 ChA 254nm		12.027	30765	0.15	1984
9	W2489 ChA 254nm	峰10	15.839	12260	0.06	504
10	W2489 ChA 254nm	峰3	16.758	19953508	98.09	771186
11	W2489 ChA 254nm	峰11	19.084	345057	1.70	8751

The HPLC of racemic 4d

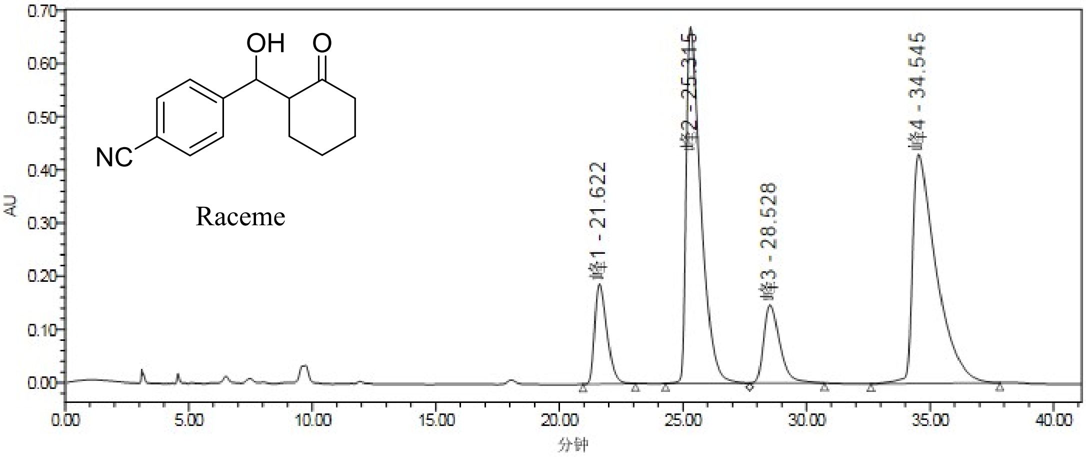

	Channel Description	Peak Name	RT (min)	Area (礦*sec)	% Area	Height (礦)
1	W2489 ChA 220nm	峰1	21.622	6463003	9.28	187203
2	W2489 ChA 220nm	峰2	25.315	27662892	39.72	669023
3	W2489 ChA 220nm	峰3	28.528	6705865	9.63	147013
4	W2489 ChA 220nm	峰4	34.545	28812899	41.37	429934

The HPLC of sample 4d

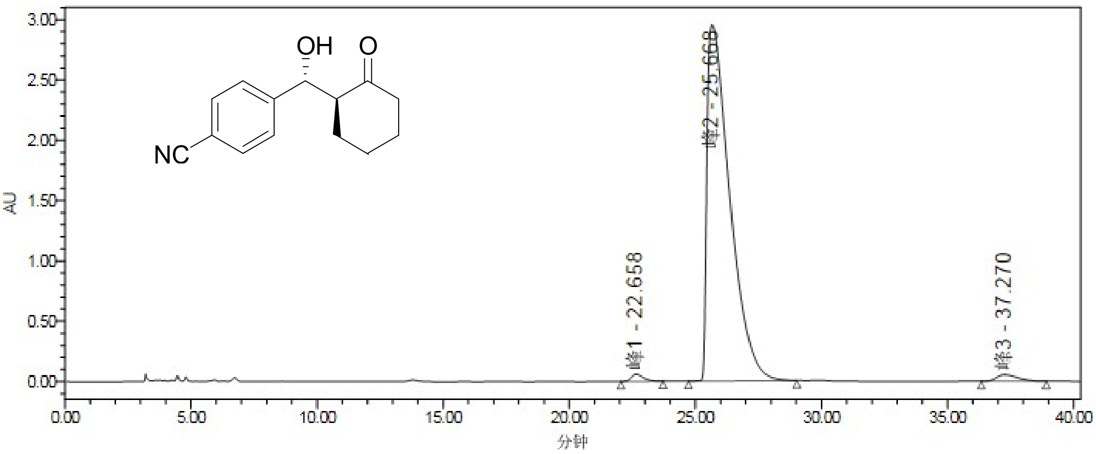

	Channel Description	Peak Name	RT (min)	Area (礦*sec)	% Area	Height (礦)
1	W2489 ChA 220nm	峰1	22.658	1956125	1.03	58424
2	W2489 ChA 220nm	峰2	25.668	184888200	97.33	2948875
3	W2489 ChA 220nm	峰3	37.270	3121587	1.64	53367

The HPLC of racemic 4e

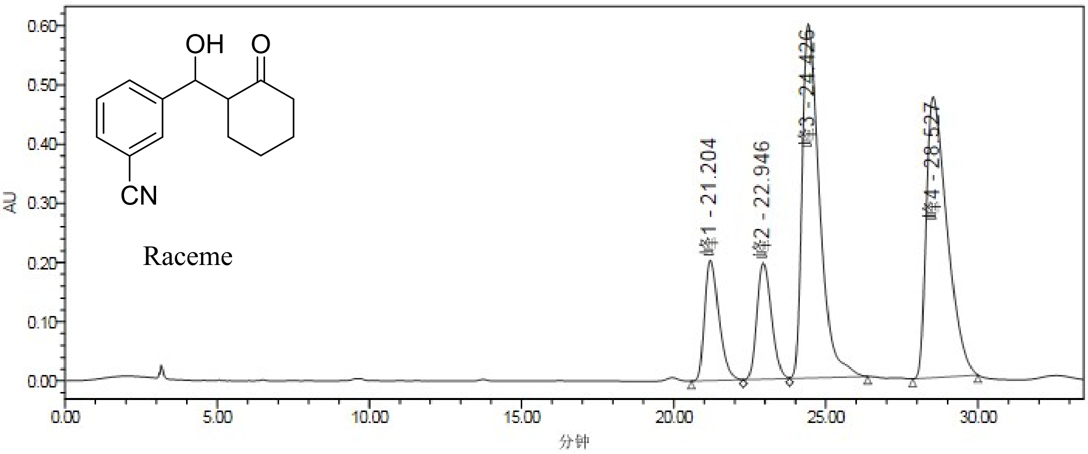

	Channel Description	Peak Name	RT (min)	Area (磺*sec)	% Area	Height (磺)
1	W2489 ChA 220nm	峰1	21.204	6664421	11.17	203018
2	W2489 ChA 220nm	峰2	22.946	6533385	10.95	195940
3	W2489 ChA 220nm	峰3	24.426	23860415	40.00	597606
4	W2489 ChA 220nm	峰4	28.527	22591821	37.87	474213

The HPLC of sample 4e

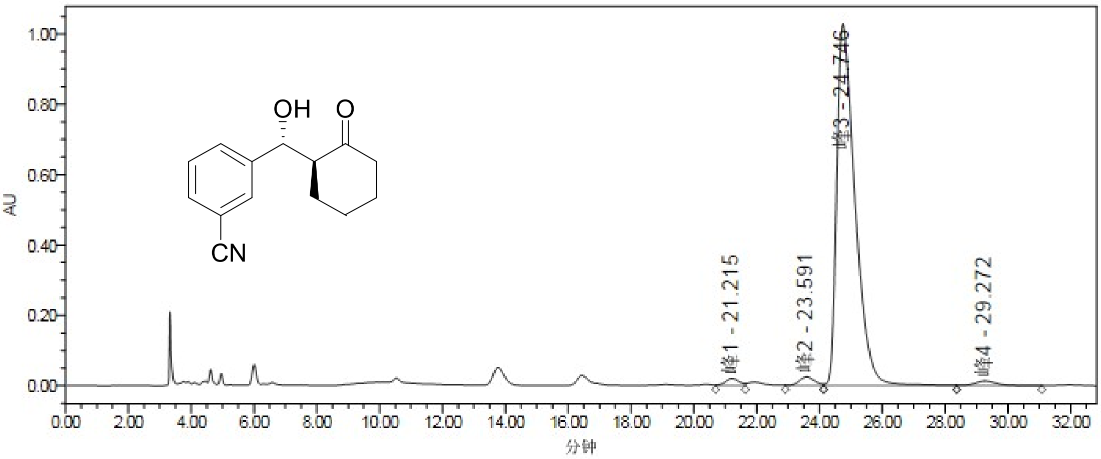

	Channel Description	Peak Name	RT (min)	Area (磺*sec)	% Area	Height (磺)
1	W2489 ChA 220nm	峰1	21.215	612644	1.40	20164
2	W2489 ChA 220nm	峰2	23.591	847830	1.94	24986
3	W2489 ChA 220nm	峰3	24.746	41621860	95.13	1027001
4	W2489 ChA 220nm	峰4	29.272	670386	1.53	13654

The HPLC of sample 4e

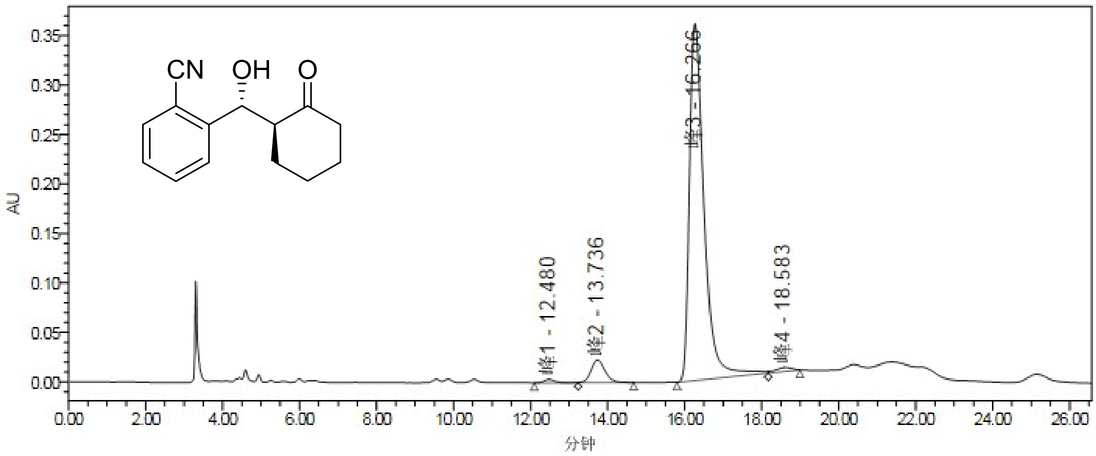

	Channel Description	Peak Name	RT (min)	Area (磺*sec)	% Area	Height (磺)
1	W2489 ChA 220nm	峰1	12.480	67226	0.66	3339
2	W2489 ChA 220nm	峰2	13.736	568614	5.57	22537
3	W2489 ChA 220nm	峰3	16.266	9457810	92.58	359722
4	W2489 ChA 220nm	峰4	18.583	121680	1.19	4091

The HPLC of racemic 4g

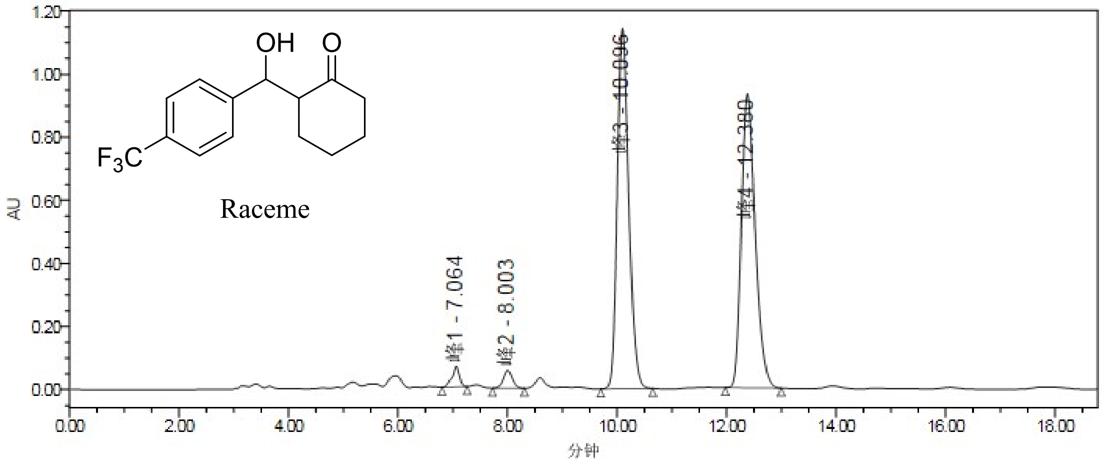

	Channel Description	Peak Name	RT (min)	Area (磺*sec)	% Area	Height (磺)
1	W2489 ChA 220nm	峰1	7.064	615009	1.80	64801
2	W2489 ChA 220nm	峰2	8.003	654842	1.91	56721
3	W2489 ChA 220nm	峰3	10.096	16613140	48.57	1141764
4	W2489 ChA 220nm	峰4	12.380	16324159	47.72	930384

The HPLC of sample 4g

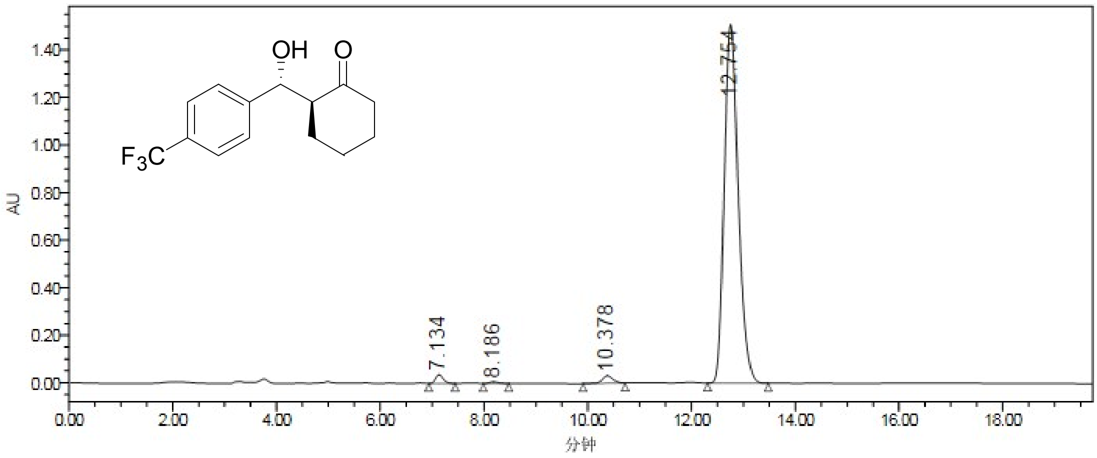

	Channel Description	RT (min)	Area (磺*sec)	% Area	Height (磺)
1	W2489 ChA 220nm	7.134	383118	1.31	37378
2	W2489 ChA 220nm	8.186	83311	0.28	7415
3	W2489 ChA 220nm	10.378	462374	1.58	32846
4	W2489 ChA 220nm	12.754	28332459	96.83	1509254

The HPLC of racemic 4h

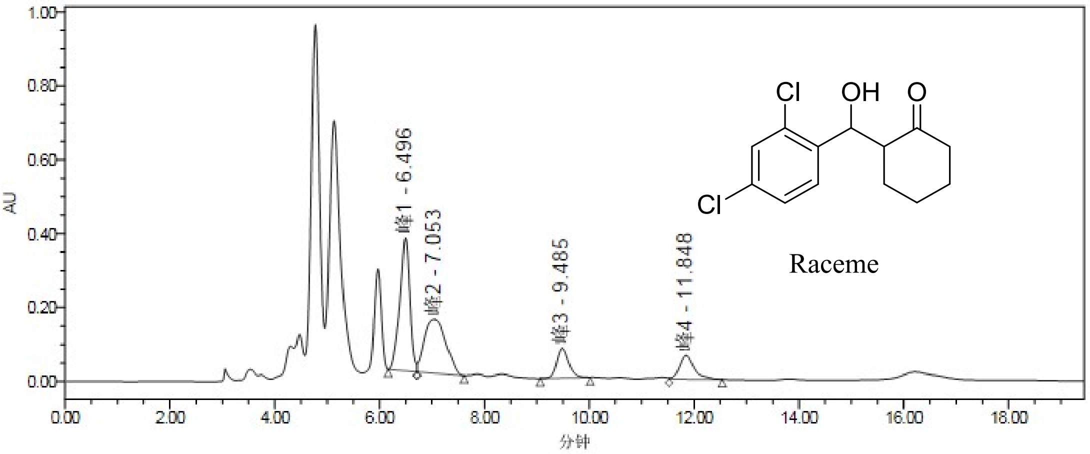

	Channel Description	Peak Name	RT (min)	Area (礦*sec)	% Area	Height (礦)
1	W2489 ChA 220nm	峰1	6.496	4779525	41.58	359733
2	W2489 ChA 220nm	峰2	7.053	4177983	36.35	145699
3	W2489 ChA 220nm	峰3	9.485	1278907	11.13	80216
4	W2489 ChA 220nm	峰4	11.848	1257992	10.94	64502

The HPLC of sample 4h

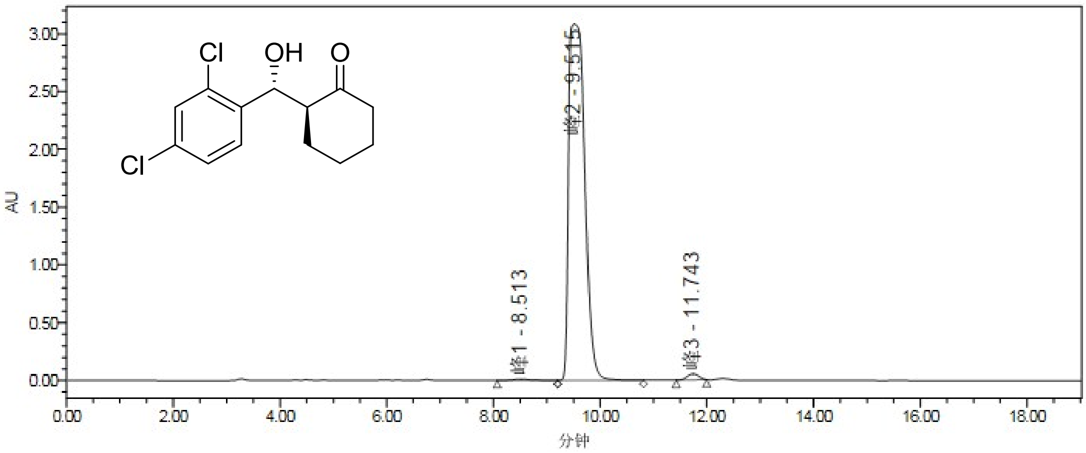

	Channel Description	Peak Name	RT (min)	Area (礦*sec)	% Area	Height (礦)
1	W2489 ChA 220nm	峰1	8.513	227762	0.35	10417
2	W2489 ChA 220nm	峰2	9.515	64224817	98.47	3080837
3	W2489 ChA 220nm	峰3	11.743	768564	1.18	50434

The HPLC of racemic 4i

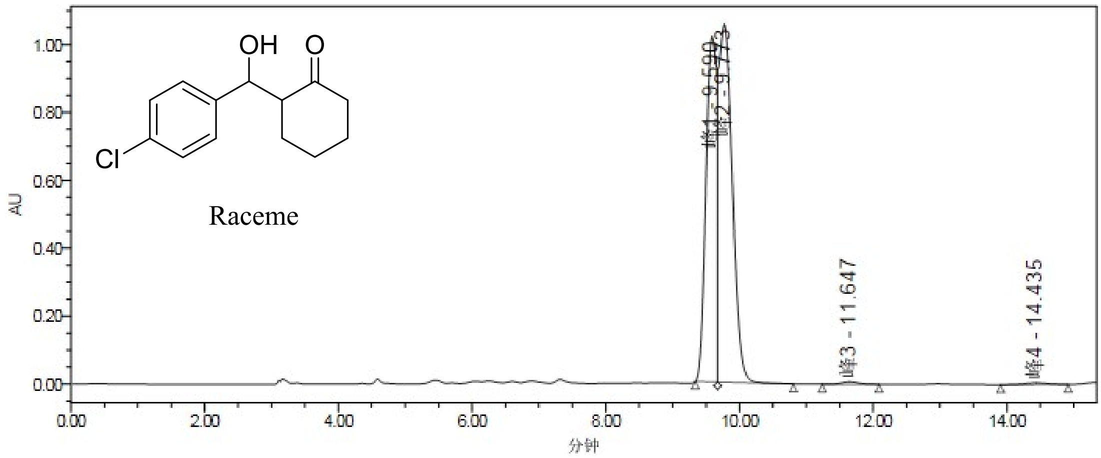

	Channel Description	Peak Name	RT (min)	Area (磺*sec)	% Area	Height (磺)
1	W2489 ChA 220nm	峰1	9.590	11219107	42.48	1016421
2	W2489 ChA 220nm	峰2	9.773	14951237	56.62	1053593
3	W2489 ChA 220nm	峰3	11.647	125988	0.48	7371
4	W2489 ChA 220nm	峰4	14.435	112076	0.42	4900

The HPLC of sample 4i

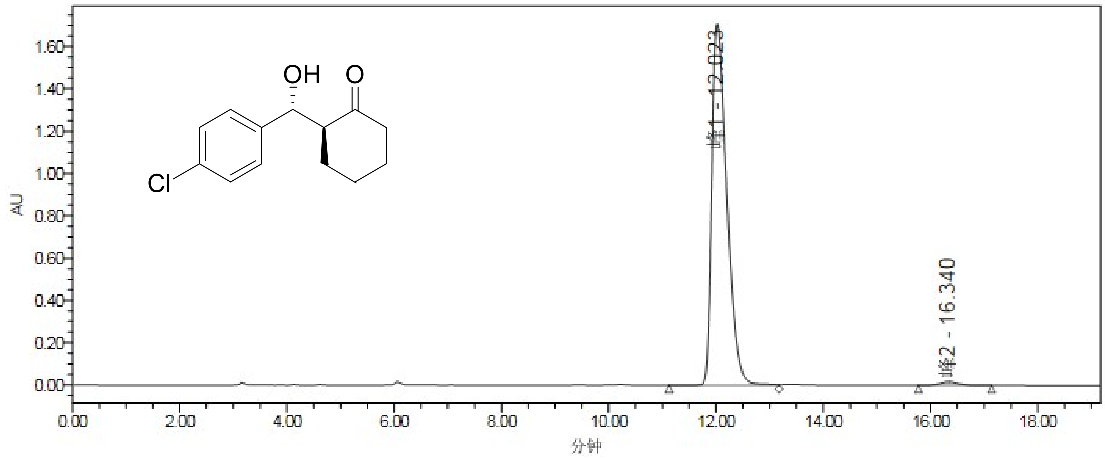

	Channel Description	Peak Name	RT (min)	Area (磺*sec)	% Area	Height (磺)
1	W2489 ChA 220nm	峰1	12.023	33056140	98.88	1706384
2	W2489 ChA 220nm	峰2	16.340	374990	1.12	15642

The HPLC of racemic 4j

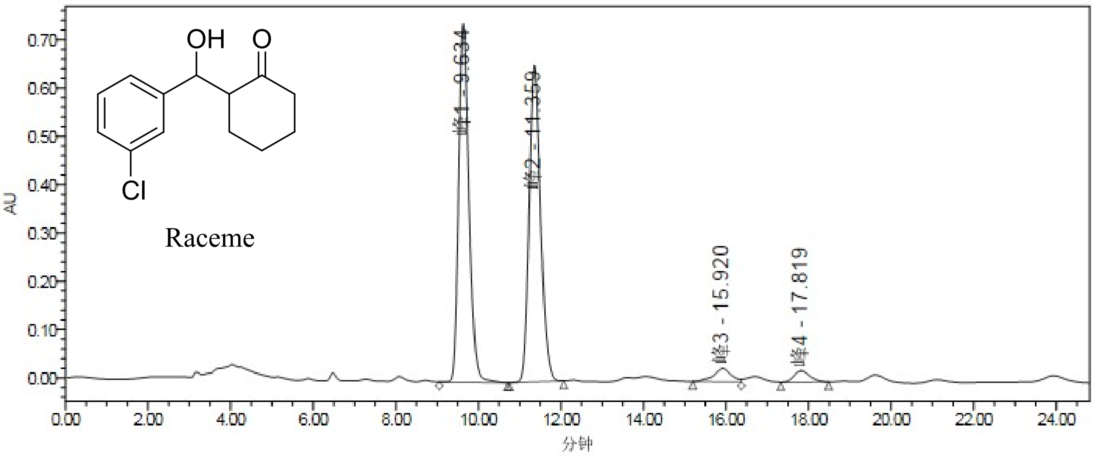

	Channel Description	Peak Name	RT (min)	Area (礦*sec)	% Area	Height (礦)
1	W2489 ChA 220nm	峰1	9.634	12713880	47.52	741168
2	W2489 ChA 220nm	峰2	11.359	12590270	47.05	655042
3	W2489 ChA 220nm	峰3	15.920	821212	3.07	27661
4	W2489 ChA 220nm	峰4	17.819	631903	2.36	23586

The HPLC of sample 4j

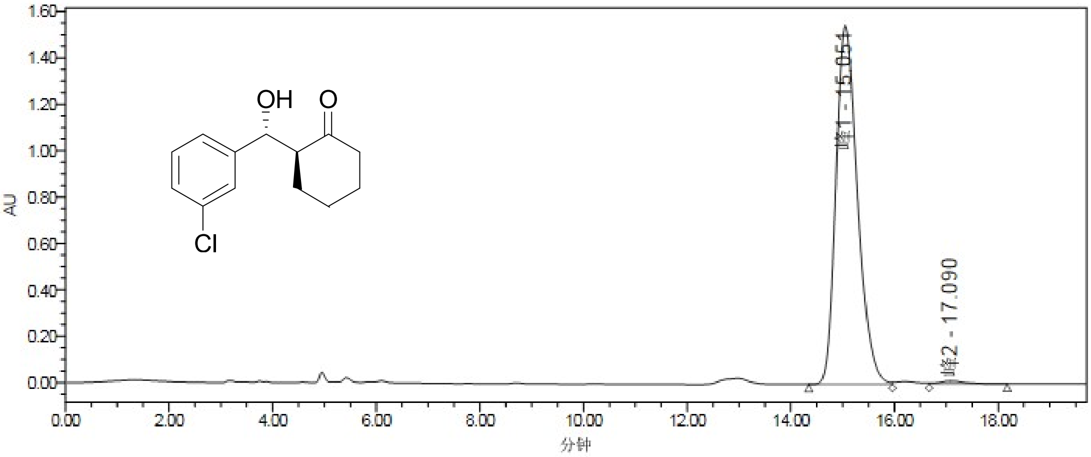

	Channel Description	Peak Name	RT (min)	Area (礦*sec)	% Area	Height (礦)
1	W2489 ChA 220nm	峰1	15.051	44575123	99.00	1544687
2	W2489 ChA 220nm	峰2	17.090	450282	1.00	13146

The HPLC of racemic 4k

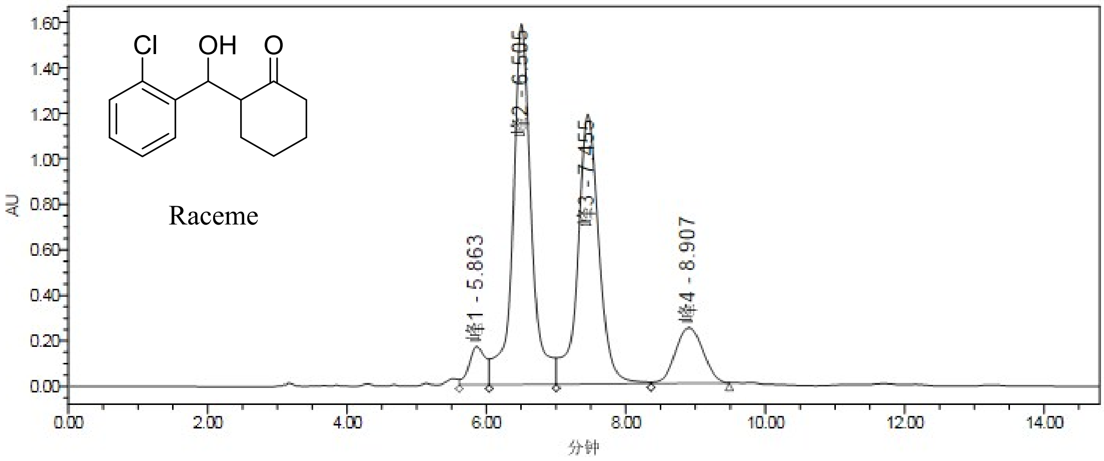

	Channel Description	Peak Name	RT (min)	Area (礦*sec)	% Area	Height (礦)
1	W2489 ChA 220nm	峰1	5.863	2746941	4.31	167613
2	W2489 ChA 220nm	峰2	6.505	29218763	45.85	1583507
3	W2489 ChA 220nm	峰3	7.455	25023586	39.27	1184437
4	W2489 ChA 220nm	峰4	8.907	6731145	10.56	243478

The HPLC of sample 4k

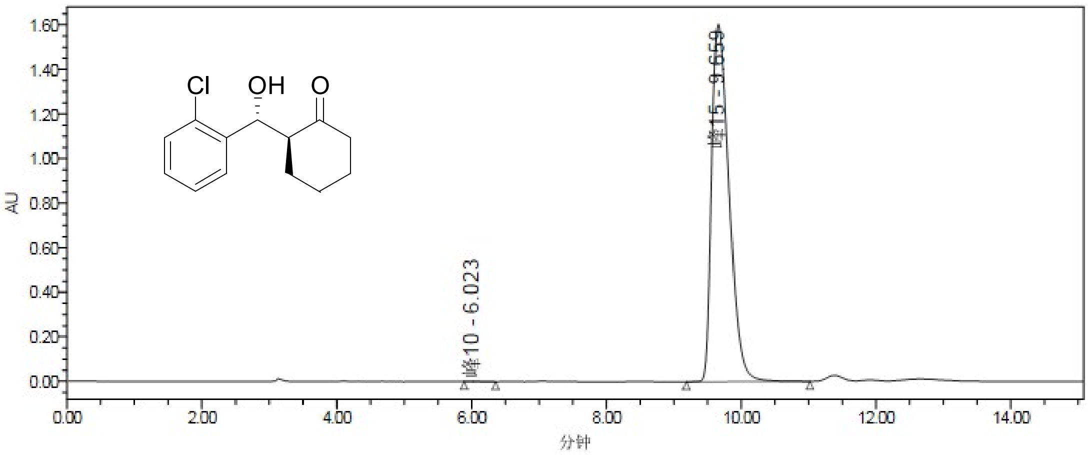

	Channel Description	Peak Name	RT (min)	Area (礦*sec)	% Area	Height (礦)
10	W2489 ChA 220nm	峰10	6.023	10964	0.04	1045
15	W2489 ChA 220nm	峰15	9.659	28855535	99.96	1601883

The HPLC of racemic 4l

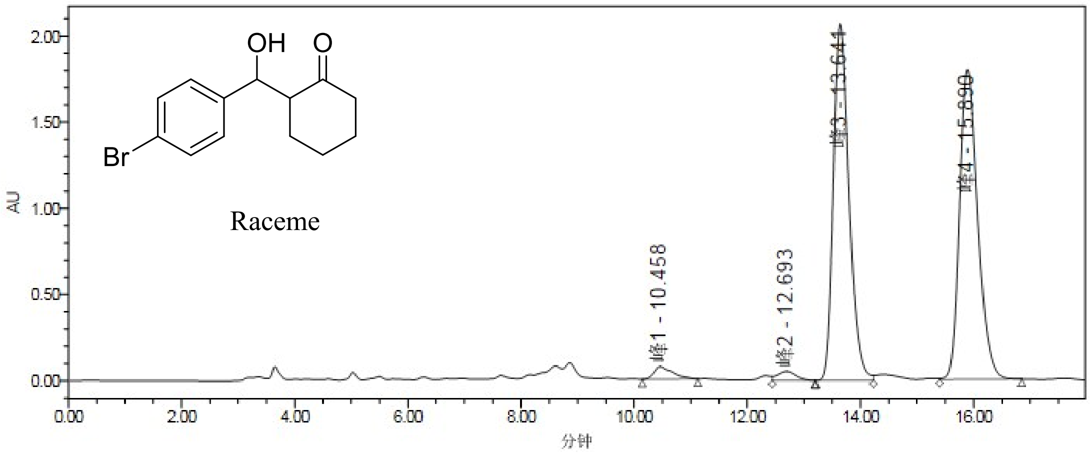

	Channel Description	Peak Name	RT (min)	Area (礦*sec)	% Area	Height (礦)
1	W2489 ChA 220nm	峰1	10.458	1627146	1.98	72745
2	W2489 ChA 220nm	峰2	12.693	1149460	1.40	51113
3	W2489 ChA 220nm	峰3	13.641	39703565	48.21	2065334
4	W2489 ChA 220nm	峰4	15.890	39873382	48.42	1795846

The HPLC of sample 4l

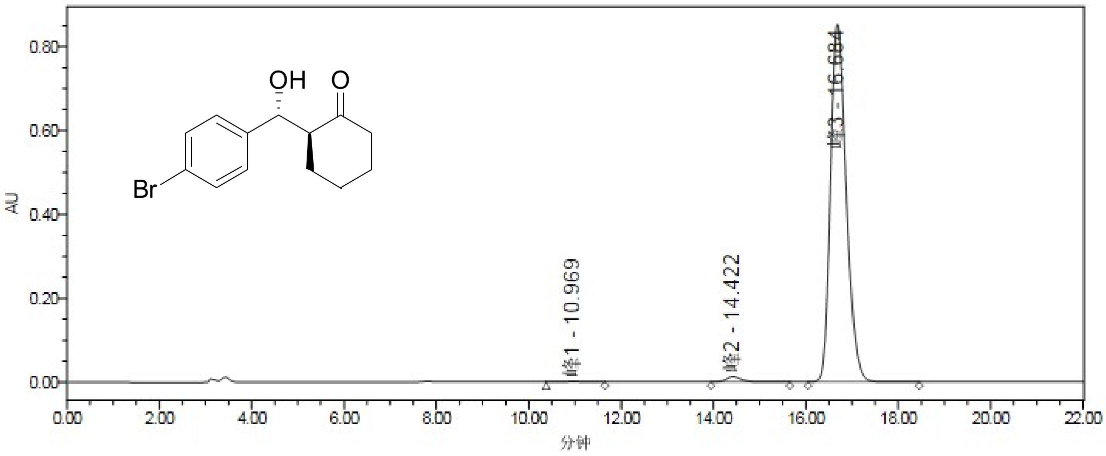

	Channel Description	Peak Name	RT (min)	Area (礦*sec)	% Area	Height (礦)
1	W2489 ChA 220nm	峰1	10.969	16047	0.08	522
2	W2489 ChA 220nm	峰2	14.422	254037	1.25	12262
3	W2489 ChA 220nm	峰3	16.684	20132946	98.68	851484

The HPLC of racemic 4m

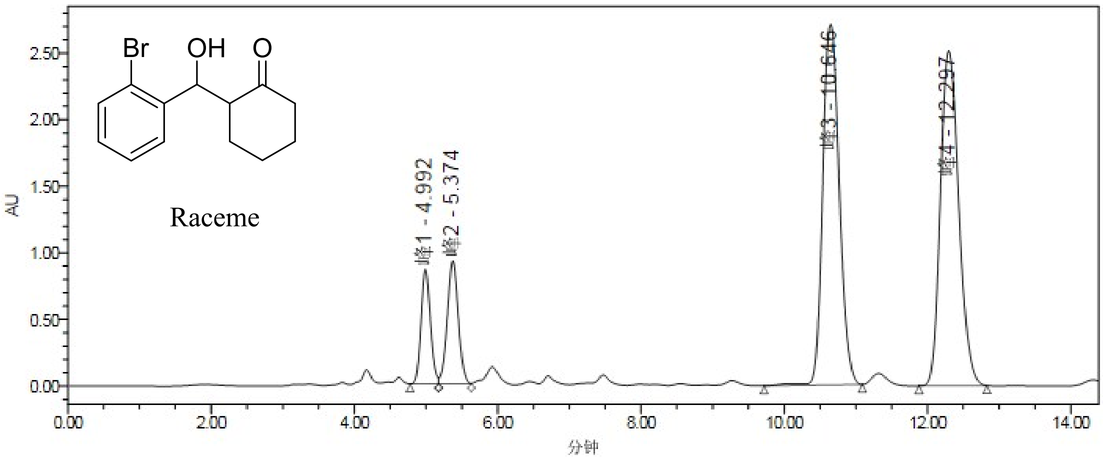

	Channel Description	Peak Name	RT (min)	Area (礦*sec)	% Area	Height (礦)
1	W2489 ChA 220nm	峰1	4.992	7785820	7.36	858460
2	W2489 ChA 220nm	峰2	5.374	9840653	9.30	922593
3	W2489 ChA 220nm	峰3	10.646	43439235	41.07	2706530
4	W2489 ChA 220nm	峰4	12.297	44692186	42.26	2514650

The HPLC of sample 4m

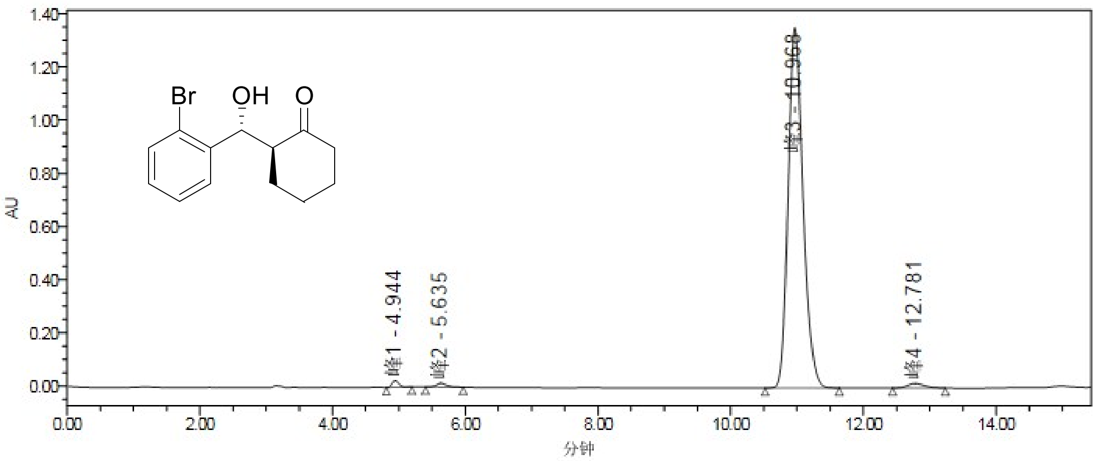

	Channel Description	Peak Name	RT (min)	Area (礦*sec)	% Area	Height (礦)
1	W2489 ChA 220nm	峰1	4.944	170314	0.79	24241
2	W2489 ChA 220nm	峰2	5.635	144616	0.67	14068
3	W2489 ChA 220nm	峰3	10.968	21030136	97.17	1352317
4	W2489 ChA 220nm	峰4	12.781	297569	1.37	16985

The HPLC of racemic 4n

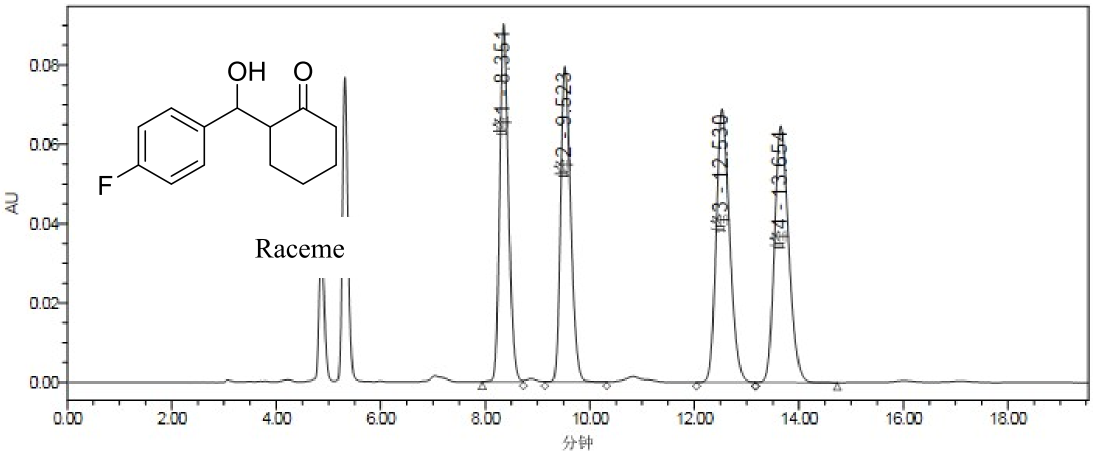

	Channel Description	Peak Name	RT (min)	Area (礦*sec)	% Area	Height (礦)
1	W2489 ChA 254nm	峰1	8.351	1061808	23.20	90167
2	W2489 ChA 254nm	峰2	9.523	1063735	23.25	79514
3	W2489 ChA 254nm	峰3	12.530	1228974	26.86	68855
4	W2489 ChA 254nm	峰4	13.654	1221327	26.69	64673

The HPLC of sample 4n

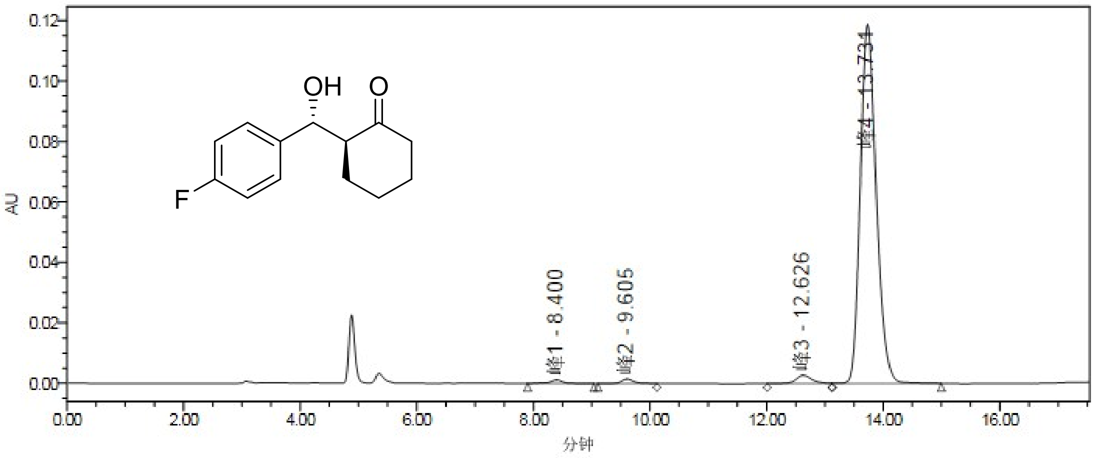

	Channel Description	Peak Name	RT (min)	Area (礦*sec)	% Area	Height (礦)
1	W2489 ChA 254nm	峰1	8.400	18939	0.80	1218
2	W2489 ChA 254nm	峰2	9.605	23387	0.99	1569
3	W2489 ChA 254nm	峰3	12.626	55104	2.34	2785
4	W2489 ChA 254nm	峰4	13.731	2260848	95.87	118734

The HPLC of racemic 4o

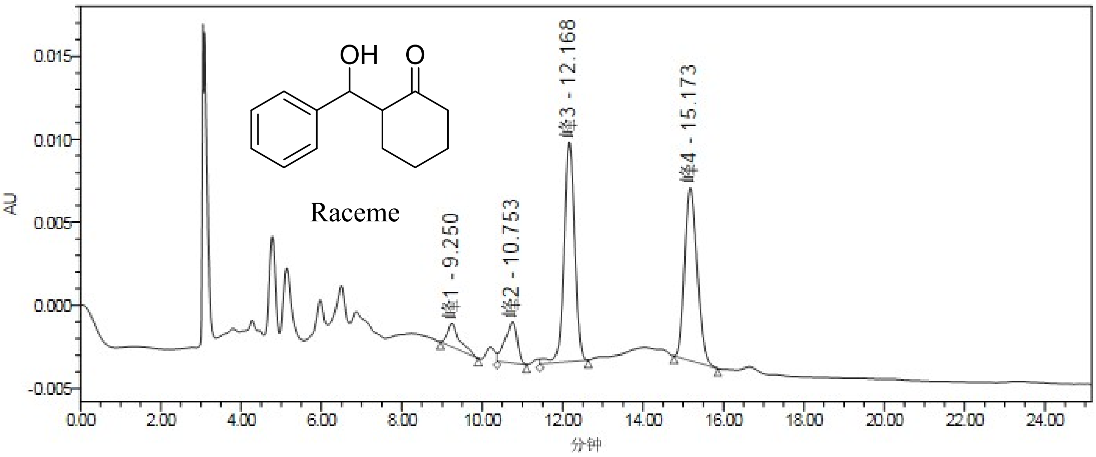

	Channel Description	Peak Name	RT (min)	Area (磺*sec)	% Area	Height (磺)
1	W2489 ChA 220nm	峰1	9.250	29761	5.40	1401
2	W2489 ChA 220nm	峰2	10.753	51764	9.40	2458
3	W2489 ChA 220nm	峰3	12.168	241292	43.81	13200
4	W2489 ChA 220nm	峰4	15.173	227894	41.38	10405

The HPLC of sample 4o

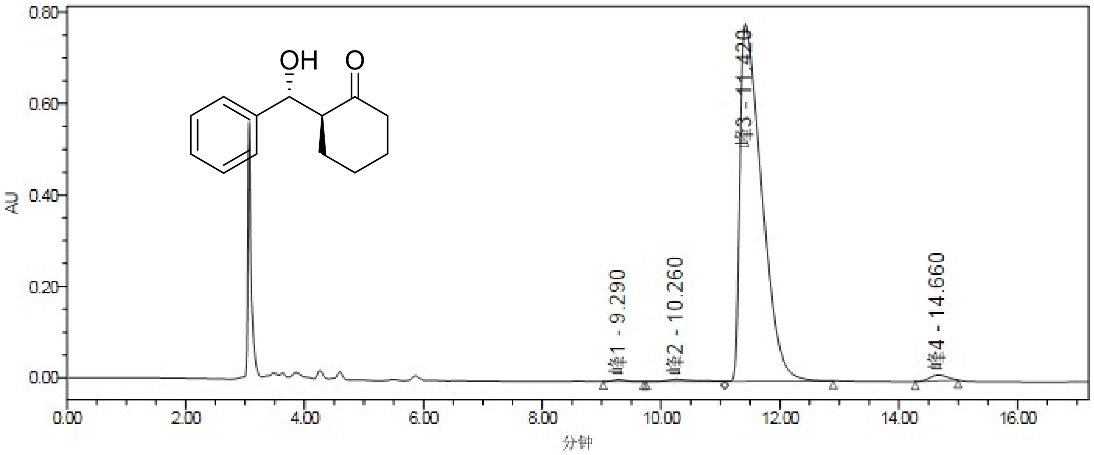

	Channel Description	Peak Name	RT (min)	Area (磺*sec)	% Area	Height (磺)
1	W2489 ChA 220nm	峰1	9.290	63144	0.31	4106
2	W2489 ChA 220nm	峰2	10.260	144965	0.71	4987
3	W2489 ChA 220nm	峰3	11.420	19898870	97.65	781392
4	W2489 ChA 220nm	峰4	14.660	271486	1.33	13037

The HPLC of racemic 4p

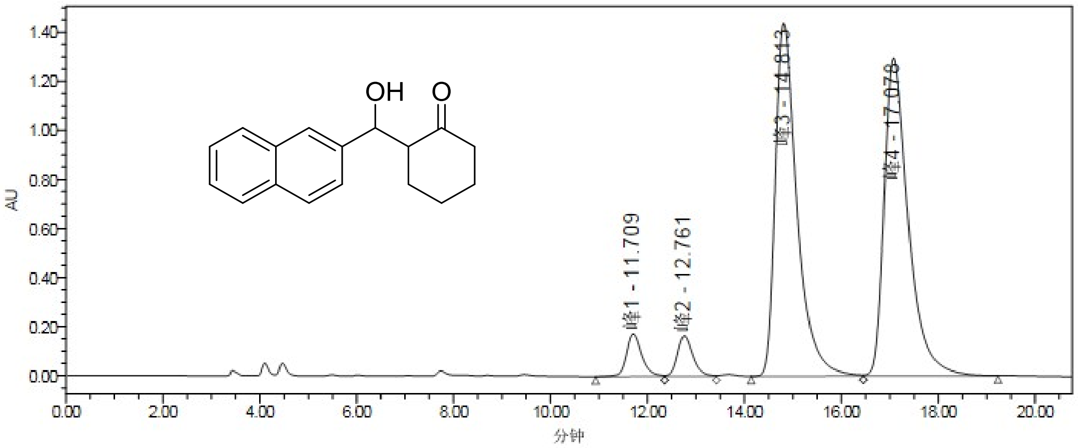

Channel: W2489 ChA; Channel Desc.: W2489 ChA 220nm; Processing Method: 071002

	Channel Description	Peak Name	RT (min)	Area (礦*sec)	% Area	Height (礦)
1	W2489 ChA 220nm	峰1	11.709	3768437	3.89	172229
2	W2489 ChA 220nm	峰2	12.761	3669100	3.79	164459
3	W2489 ChA 220nm	峰3	14.813	44659448	46.13	1435886
4	W2489 ChA 220nm	峰4	17.078	44724692	46.19	1295285

The HPLC of sample 4p

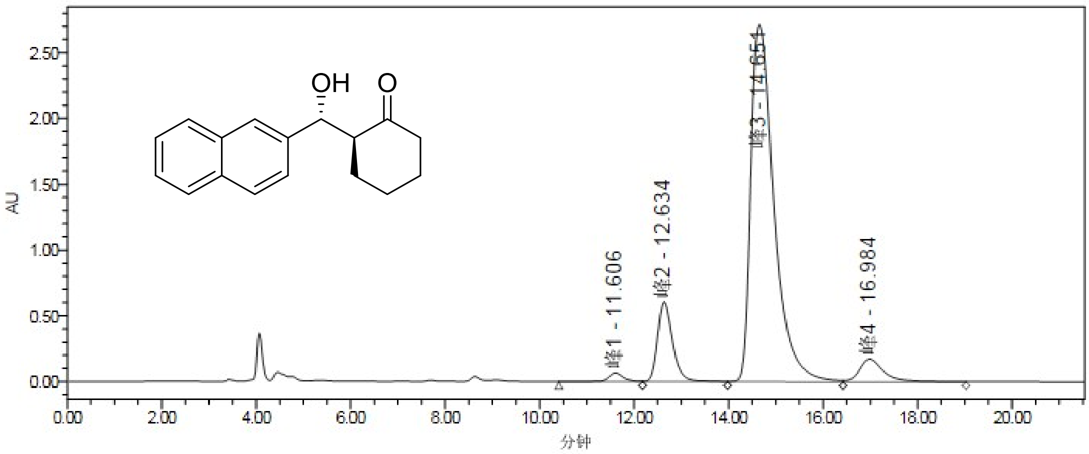

Channel: W2489 ChA; Channel Desc.: W2489 ChA 220nm; Processing Method: 071003

	Channel Description	Peak Name	RT (min)	Area (礦*sec)	% Area	Height (礦)
1	W2489 ChA 220nm	峰1	11.606	1487895	1.31	66747
2	W2489 ChA 220nm	峰2	12.634	13500014	11.86	604188
3	W2489 ChA 220nm	峰3	14.651	92696286	81.45	2711364
4	W2489 ChA 220nm	峰4	16.984	6123788	5.38	171278

The HPLC of racemic 4t

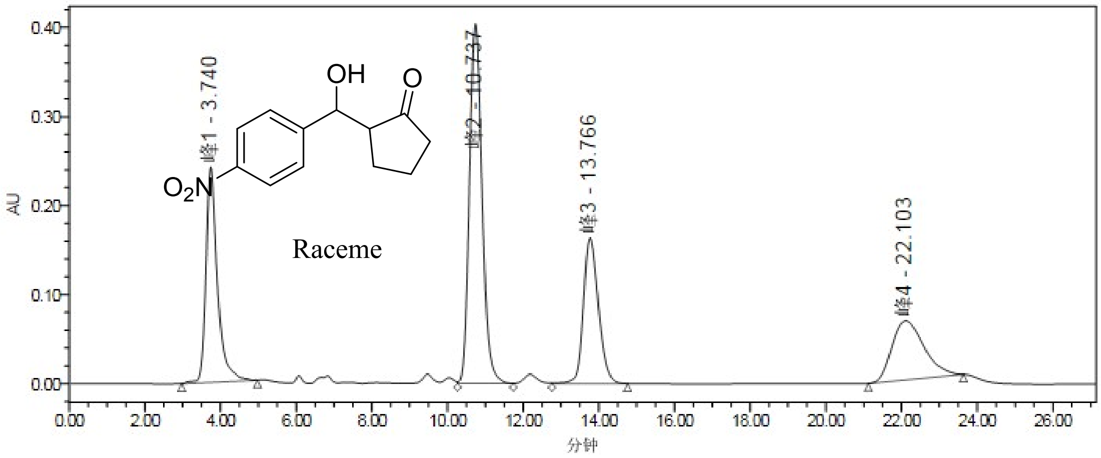

	Channel Description	Peak Name	RT (min)	Area (礦*sec)	% Area	Height (礦)
1	W2489 ChA 254nm	峰1	3.740	5103049	22.28	241971
2	W2489 ChA 254nm	峰2	10.737	9370531	40.92	403611
3	W2489 ChA 254nm	峰3	13.766	4515754	19.72	163357
4	W2489 ChA 254nm	峰4	22.103	3911386	17.08	66257

The HPLC of sample 4t

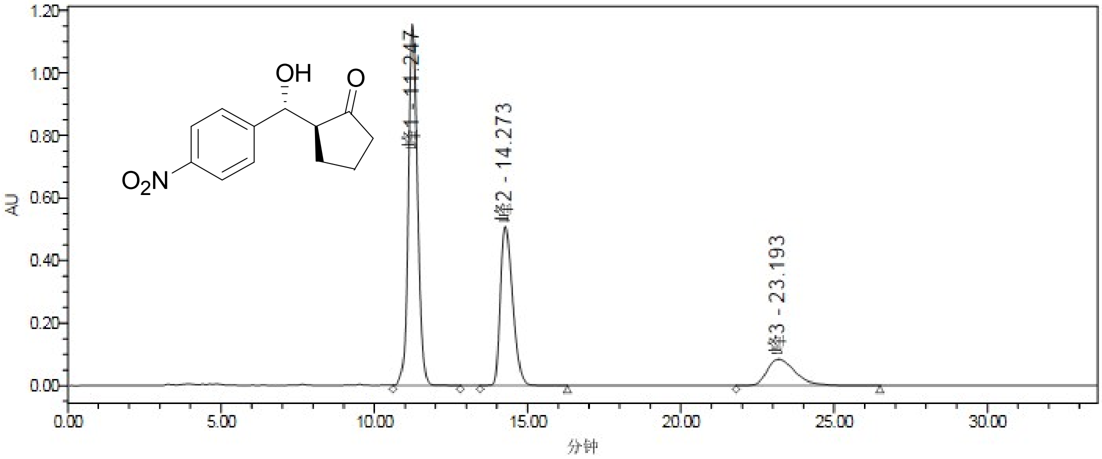

	Channel Description	Peak Name	RT (min)	Area (礦*sec)	% Area	Height (礦)
1	W2489 ChA 254nm	峰1	11.247	23945199	55.18	1154057
2	W2489 ChA 254nm	峰2	14.273	14129024	32.56	507244
3	W2489 ChA 254nm	峰3	23.193	5321504	12.26	84419

The HPLC of racemic 4u

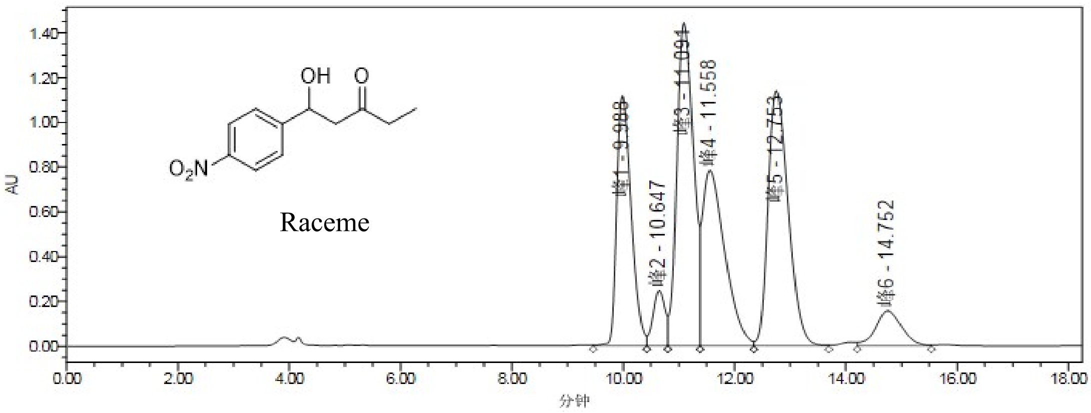

	Channel Description	Peak Name	RT (min)	Area (礦*sec)	% Area	Height (礦)
1	W2489 ChA 254nm	峰1	9.988	20109697	18.36	1114116
2	W2489 ChA 254nm	峰2	10.647	3658587	3.34	245985
3	W2489 ChA 254nm	峰3	11.091	29816550	27.23	1440436
4	W2489 ChA 254nm	峰4	11.558	22066358	20.15	783402
5	W2489 ChA 254nm	峰5	12.753	28717563	26.22	1138533
6	W2489 ChA 254nm	峰6	14.752	5147721	4.70	156407

The HPLC of sample 4u

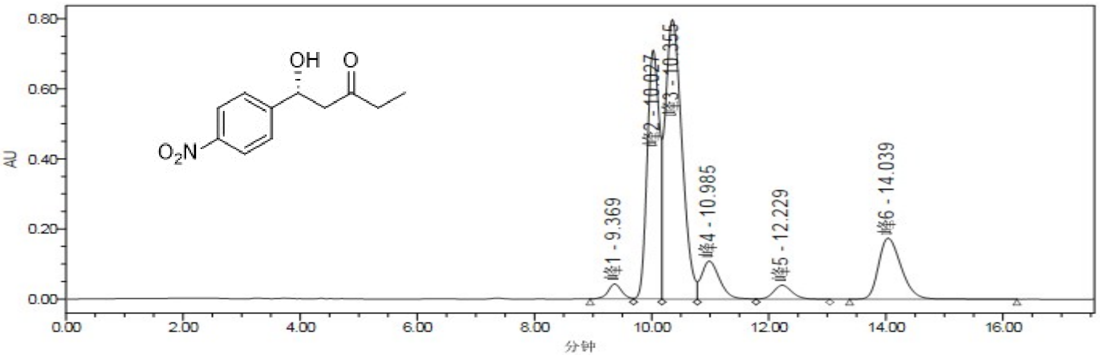

	Channel Description	Peak Name	RT (min)	Area (礦*sec)	% Area	Height (礦)
1	W2489 ChA 254nm	峰1	9.369	691161	1.99	42788
2	W2489 ChA 254nm	峰2	10.027	10247219	29.48	709897
3	W2489 ChA 254nm	峰3	10.355	15952728	45.90	797095
4	W2489 ChA 254nm	峰4	10.985	2327133	6.70	108143
5	W2489 ChA 254nm	峰5	12.229	865147	2.49	39690
6	W2489 ChA 254nm	峰6	14.039	4672779	13.44	173807

The HPLC of sample 4v

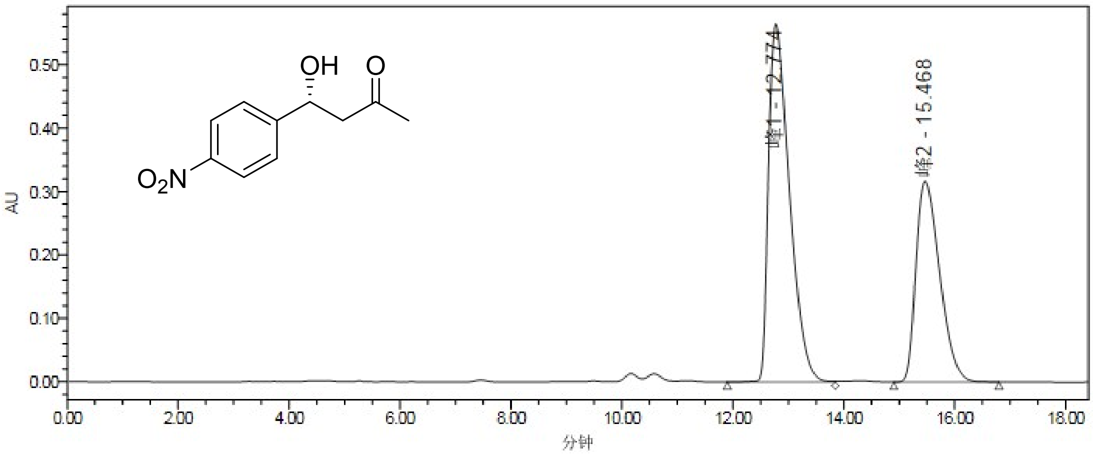

	Channel Description	Peak Name	RT (min)	Area (磺*sec)	% Area	Height (磺)
1	W2489 ChA 254nm	峰1	12.774	14732285	60.99	564396
2	W2489 ChA 254nm	峰2	15.468	9422943	39.01	316392

5. References

[1] S. P. Zhang,X. K. Fu and X. D. Fu, *Tetrahedron Letters*, 2009, **50**, 1173.

[2] H. Tian, J. L. Gao, H. Xu, L. Y. Zheng, W. B. Huan,Q. W. Liu and S. Q. Zhang, *Tetrahedron: Asymmetry*, 2011, **22**, 1074.

[3] Y. Li, Q. C. Yang,X. Y. Xu,Y. Zhou, J. F. Bai, F. Y. Wang and Wang, L. X. *Can. J. Chem*. 2011, **89**, 1312.

[4] B. H. Xie, W. Li, Y. Liu,H. H. Li, Z. Guan and Y. H. He, *Tetrahedron*, 2012, **68**, 3160.

[5] S. Li, C. L. Wu, X. Q. Long, X. K. Fu, G. D. Chen and Z. J. Liu, *Catalysis Science & Technology,* 2012, **2**, 1068.

[6] R. Wang, Z. L. Wei, J. Guo,Y. S. Feng, E. J. Xu,H. F. Duan,Y. J. Lin,Q. B. Yang,J. S. Du and Y. X. Li, Chem. Res. Chin. Univ. 2018, **34**, 180.
